# Updated seismotectonic framework of Abu Dabbab Egypt based on focal mechanisms and stress inversion

**DOI:** 10.1038/s41598-026-36922-3

**Published:** 2026-02-14

**Authors:** Mona Abdelazim, Salah Elhadidy Youssef, Hanan Gaber, Gad-Elkareem A. Mohamed, M. Sami Soliman, Mohamed H. Yassien, Mona Hamada, Shimaa H. Elkhouly

**Affiliations:** 1 Egyptian National Data Center (ENDC), National Research Institute of Astronomy and Geophysics (NRIAG), Elmarsad Street, Helwan, Cairo, 11421 Egypt; 2 Seismology Department, National Research Institute of Astronomy and Geophysics (NRIAG), Elmarsad Street, Helwan, Cairo, 11421 Egypt

**Keywords:** Seismicity, Focal mechanism, Stress tensor, Abu Dabab, Egypt, Natural hazards, Solid Earth sciences

## Abstract

The Abu Dabbab seismic zone is located along Egypt’s Red Sea margin, stands out as one of the most active seismic regions in the Eastern Desert. Characterized by frequent micro earthquakes, swarm like activity, and notable historical events. To enhance understanding of its tectonic framework, 408 earthquakes (Ml 0.7–3.0) recorded in 2004 were analyzed using digital waveform data from a temporary local seismic network consisting of ten vertical short period seismometers. Focal mechanisms were determined from P-wave first-motion polarities and classified using ternary plots. The analysis revealed a diverse range of faulting styles normal, strike slip, reverse, and oblique with clear depth dependent patterns. Shallow events (0–5 km) were dominated by normal and strike slip faulting, intermediate depths (5–10 km) showed increased reverse and oblique components, while deeper events (> 10 km) were primarily normal faulting. Stress tensor inversion across three depth intervals indicated a multiphase stress regime: shallow depths exhibited alternating faulting styles due to localized stress variations; intermediate depths revealed a heterogeneous stress field with mixed faulting regimes; and deeper levels showed a dominant normal faulting regime, consistent with the extensional tectonics of the Red Sea Rift. Overall, the stress field is shaped by NE–SW compression and SE–NW extension, with deformation concentrated along NW–SE and NE–SW trending faults. These findings underscore the combined influence of regional rift-related extension and local factors such as magmatic intrusions and crustal heterogeneity in driving seismicity at Abu Dabbab. This study yields important insights into depth dependent stress patterns and active faulting, enhancing seismic hazard assessments and highlighting the region’s potential as a sustainable geothermal energy source within a tectonically dynamic environment.

**Supplementary Information:**

The online version contains supplementary material available at 10.1038/s41598-026-36922-3.

## Introduction

Abu Dabbab is situated along Egypt’s Red Sea coast, approximately 24 km inland and 30 km north of Marsa Alam (25.15°–25.35°N, 34.35°–34.65°E) it is located within the tectonically active Eastern Desert, it forms part of the Red Sea Rift System a segment of the Afro Arabian Rift shaped by the divergent motion of the African and Arabian plates. This region is notable for recurrent micro earthquake swarms, such as those recorded in 1984 and between 2004 and 2005, which occurred without a distinct mainshock. The name “Abu Dabbab” itself carries seismic significance, translating to “earthquake sounds” (from Dabbab meaning knocking, and Abu meaning father) a phenomenon long described by local Bedouin communities who reported hearing these mysterious subterranean sounds. Strategically positioned at the intersection of major tectonic and structural trends, Abu Dabbab is recognized as one of Egypt’s most seismically active zones.

The region has long intrigued researchers due to its persistent and anomalous seismic activity. Early studies by^1–3^ documented major events such as the 1955 (mb = 6) and 1984 (M = 5.1) earthquakes and proposed that the seismicity might be related to a plutonic intrusion within the Precambrian crust, rather than solely regional tectonic forces. This view was supported by the tight clustering of hypocenters and sustained swarm-like activity. Additional studies have linked the region’s seismic behavior to igneous intrusions and potential magma sources, citing its unusually high heat flow (~ 92 mW/m^2^), nearly double the national average^4,5^ and seismic tomography revealing P and S wave anomalies indicative of magma intrusion beneath the area^6^ while observations of co seismic surface deformation near earthquake source zones point to a heterogeneous crustal structure^7,8^. These findings suggest a complex interplay between tectonic stress and magmatic processes in driving Abu Dabbab’s seismic dynamics. Despite these findings, debate continues, with some researchers suggesting that both tectonic stress and magmatic processes contribute to the area’s seismic behavior^9,10^. Abu Dabbab’s tectonic setting is intimately connected to its persistent seismic activity, as documented by numerous researchers^11,12^. In addition to the major earthquakes of 1955 and 1984, the region has experienced recurring seismic swarms, frequently accompanied by audible rumbling sounds reported by local communities^5^. Notable swarms were recorded in 1976, 1984, and 1993^6,9,11^.

The faulting mechanisms in Abu Dabbab have evolved over time, reflecting the region’s complex tectonic dynamics. The 1955 earthquake analysis by^13^ revealed a strike slip faulting mechanism with a normal dip slip component, with nodal planes trending NNW–NW and ENE–ESE. In contrast, the 1984 event exhibited strike slip faulting with a normal component^10^. During the August 2004 seismic swarm,^9^ documented a shift in faulting style: while the background seismicity was dominated by normal faulting with strike slip elements, the swarm itself was characterized by reverse faulting with strike slip components. Consistent fault orientations NW, NE, ENE–WNW, and NNE–NNW were interpreted as indicative of left lateral strike slip faulting.^10^ further identified thrust faulting mechanisms during the 2004 swarm, contrasting with earlier events such as the 1984 earthquake, which showed strike slip faulting with a normal component. These findings suggest right lateral slip along NE–SW faults and left lateral slip along NW–SE faults, with T-axis trends ranging from NNE–SSW to NNW–SSE. This pattern reflects the reactivation of structures associated with the Najd Fault System. Subsequent studies confirmed the coexistence of normal, reverse, and strike-slip faulting styles in the region^14–16^. More recently,^17^ reported NE–SW compression and SE–NW extension, consistent with broader regional strain patterns.

The Abu Dabbab region hosts extensive geological formations with ultra basic to basic intrusions, including flows, dikes, and massive bodies. It is defined by three major wadis Abu Dabbab, Mubarak, and Dabr. Wadi Mubarak, in the north, contains thick sequences of low grade volcanic sedimentary rocks of back arc and arc affinities, intruded by both older and younger granitic bodies^18,19^.The broader Eastern Desert of Egypt, particularly along the Red Sea margin, holds significant geothermal energy potential, supporting Egypt’s strategy to expand sustainable energy resources in tectonically active areas.

In regions with geological and tectonic settings comparable to Abu Dabbabsuch as the Gediz Graben in western Anatoliaresearchers have employed diverse geophysical techniques to investigate crustal processes. These areas are characterized by active extensional tectonics, pronounced lateral variations in sedimentary cover thickness, and significant magmatic activity^20,21^ advanced this work by integrating continuous, high resolution seismic monitoring with complementary geophysical surveys, including magneto telluric and state of the art tomography. This combined methodology enables precise imaging of intrusion geometries, monitoring of stress evolution, and direct evaluation of geothermal systems. Such integration is essential for unraveling the dynamics of active rift margins and plays a critical role in reducing exploration risks for renewable energy projects in tectonically complex environments.

Although seismicity in Abu Dabbab has been widely studied, detailed stress tensor inversion analyses are limited. Earlier work revealed a clockwise rotation of the T‑axis and a complex strain regime, highlighting the interplay of compressional and extensional forces.

This study advances the field by analyzing focal mechanism solutions for 408 micro earthquakes (Ml 0.7–3.0) recorded in 2004, enabling a comprehensive, depth dependent stress tensor inversion. Results show deformation patterns consistent with regional tectonic stress orientations, with seismicity driven by stress concentrations near the tip of a propagating intrusion.The findings improve understanding of depth dependent stress regimes and active faulting, strengthen seismic hazard assessments, and emphasize Abu Dabbab’s potential as a sustainable geothermal energy resource in a tectonically dynamic setting.

## Data source and methodology

The present study utilizes raw waveform data recorded in 2004 by ten temporary seismic stations deployed in the Abu Dabbab region by the Egyptian National Seismological Network (ENSN). Earthquakes were extracted from the raw data and located using the HYPOINVERSE software, based on the crustal velocity model of^22^. To better understand the tectonic framework of the study area, the analysis was conducted separately for each month. In 2004, an intense earthquake swarm occurred, producing more than 4,000 recorded events within just a few months. From this sequence, 408 earthquakes were selected for detailed examination in order to identify any apparent changes in focal mechanisms and stress tensor during the swarm period.

Fault plane solutions were determined for 408 earthquakes with local magnitudes ranging from 0.7 to 3.0 using the classical first motion polarity method of P waves by PMAN software^23^ to obtain solutions depending on the azimuth, incidence angle, and polarities of P-phase only. Furthermore,^22^ demonstrates the importance of high precision earthquake relocation and cluster analysis in deciphering seismogenic processes.

QP was calculated for all focal mechanism solutions where QP is the quality code that describes the range of uncertainties in the strike, dip, and rake of a focal mechanism solution. To approach this purpose we should obtain acceptable solutions for each event thus we used the FOCMEC software^24^, which performs a grid search over all possible solutions based on user selected parameters, including polarity errors and the allowable disagreement between observed and calculated solutions. In this study, solutions were estimated using a 5° grid search. QP and azimuth gap for each event are illustrated in supplementary material.

In this study, we applied the stress tensor inversion technique of^25^ to characterize the stress field in the Abu Dabbab region. Stress tensor inversion methods, originally developed by^26–29^ and later refined by^29,30^, assume that: The regional tectonic stress is homogeneous, Earthquakes occur on pre-existing faults with varying orientations, and Slip on a fault plane occurs in the direction of maximum shear stress (τ), according to the Wallacem Bott hypothesis^31,32^.^32^ defined four independent parameters describing the reduced stress tensor: the principal stresses σ_1_, σ_2_, σ_3_, and the stress ratio R, which governs the orientation of shear stress on fault planes. We used TENSOR software^25^ to estimate these parameters. Focal mechanisms incompatible with the predominant stress field are filtered out. Horizontal stress orientations (SHmax and Sh_min_) are computed following^33^. Recent advances, such as the framework proposed by^34,35^, emphasize the integration of multi depth and multi-fault kinematic data for stress tensor inversion, particularly for hidden seismogenic faults. Their methodology highlights the necessity of detailed fault plane solutions and stress ratio assessments in complex tectonic settings, which aligns closely with our depth-dependent stress regime analysis in Abu Dabbab.

The quality of the stress inversion results is evaluated using the World Stress Map (WSM) ranking system^25,36^:

- A-quality: N ≥ 15 events, α ≤ 12°, SHmax/SHmin ± 15°
- B-quality: 8 < N < 15, 12° < α ≤ 20°, SHmax/SHmin ± 15–20°
- C-quality: 6 ≤ N < 8 or α > 20°, SHmax/SHmin ± 20–25°
- D-quality: Only 4–5 events per box, SHmax/SHmin ± 25–40°

This approach allows robust, depth dependent characterization of the stress field based on the compiled focal mechanism solutions from the Abu Dabbab region.

## Seismicity in Abu Dabbab seismic zone

The Abu Dabbab region in Egypt’s Eastern Desert has long intrigued researchers due to its persistent and anomalous seismic activity.^1^, using WWSSN data to analyze Red Sea seismicity since 1953, reported a major earthquake on 12 November 1955 (mb = 6), followed by a moderate event on 2 July 1984 (M = 5.1).^2,3^ attributed this seismicity to a plutonic intrusion within the Precambrian crust, rather than to regional tectonic forces a view supported by the tight clustering of hypocenters and the sustained activity over time. Several studies have linked seismic activity in Abu Dabbab to igneous intrusions and underlying magma sources, citing the region’s exceptionally high heat flow (approximately 92 mW/m^2^, nearly twice the national average)^4,5^. Despite this thermal evidence, the origin of the seismicity remains a topic of ongoing debate. Some researchers argue that both tectonic stress and magmatic processes jointly contribute to the area’s complex seismic behavior^9,10^.

**Fig. 1:**
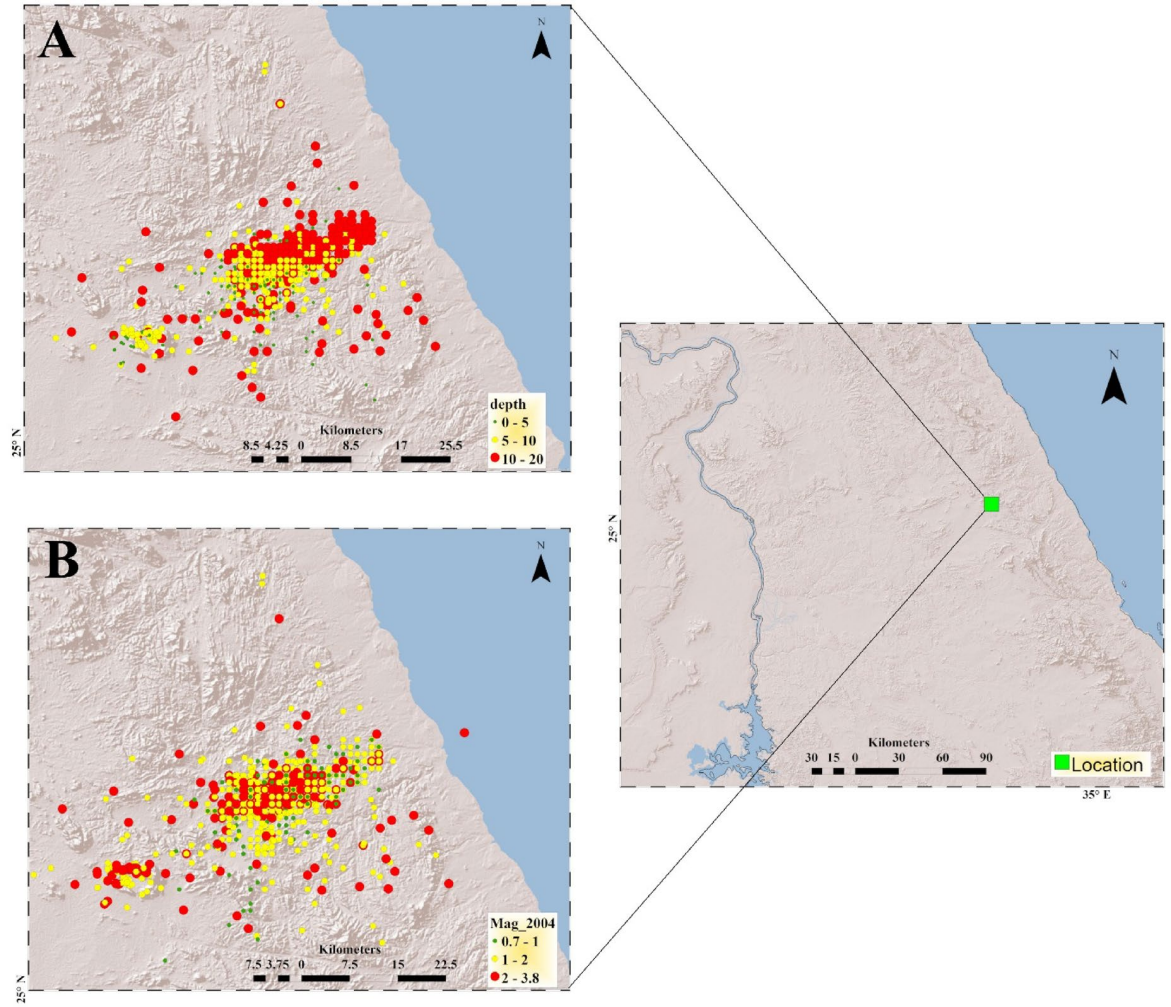
Location map where the green square represent the area of study. (A) Seismicity map categorized by depth ranges while(B) Seismicity map categorized by magnitude ranges.

**Fig. 2:**
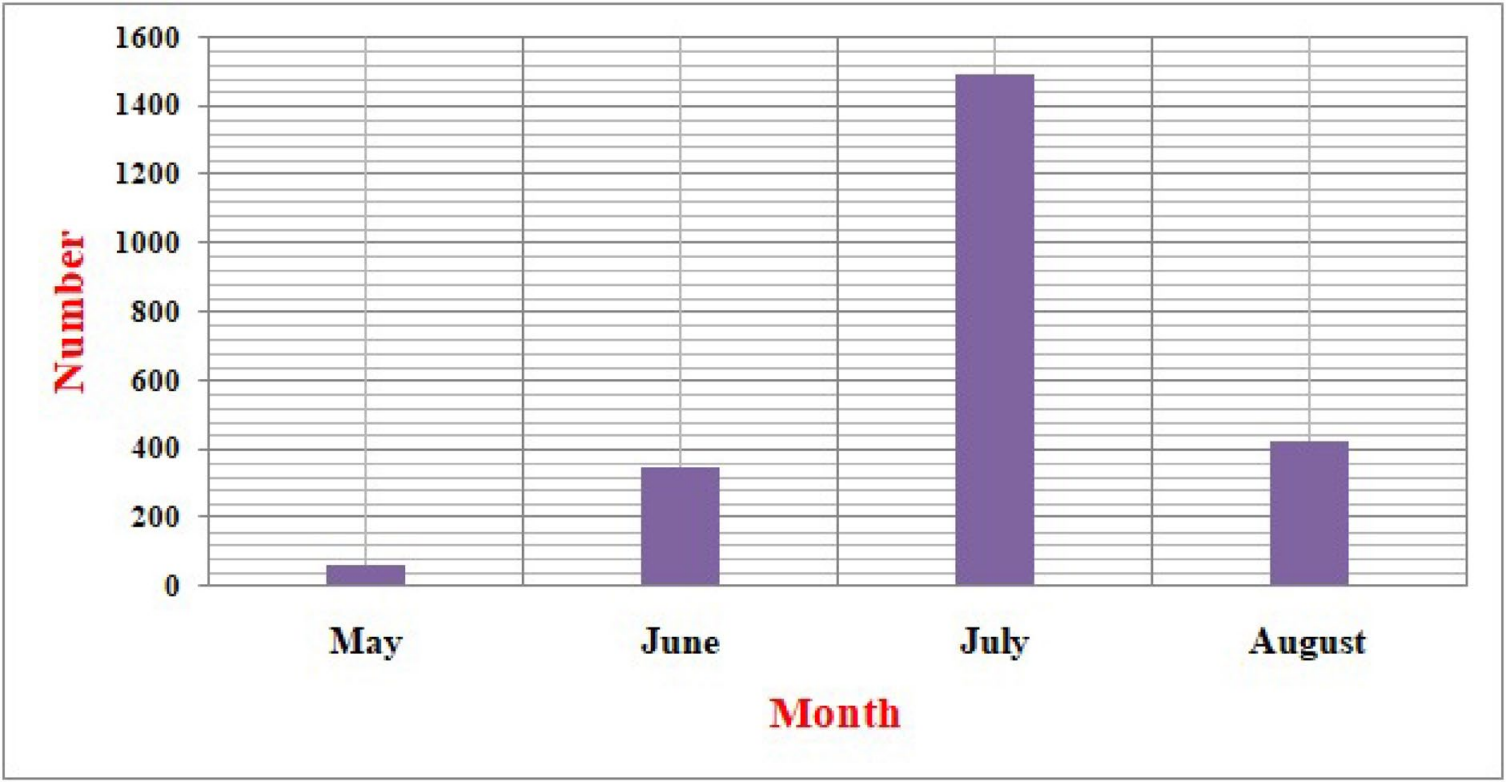
Histogram show the relocated earthquakes in Abu Dabbab area during 2004.

**Fig. 3:**
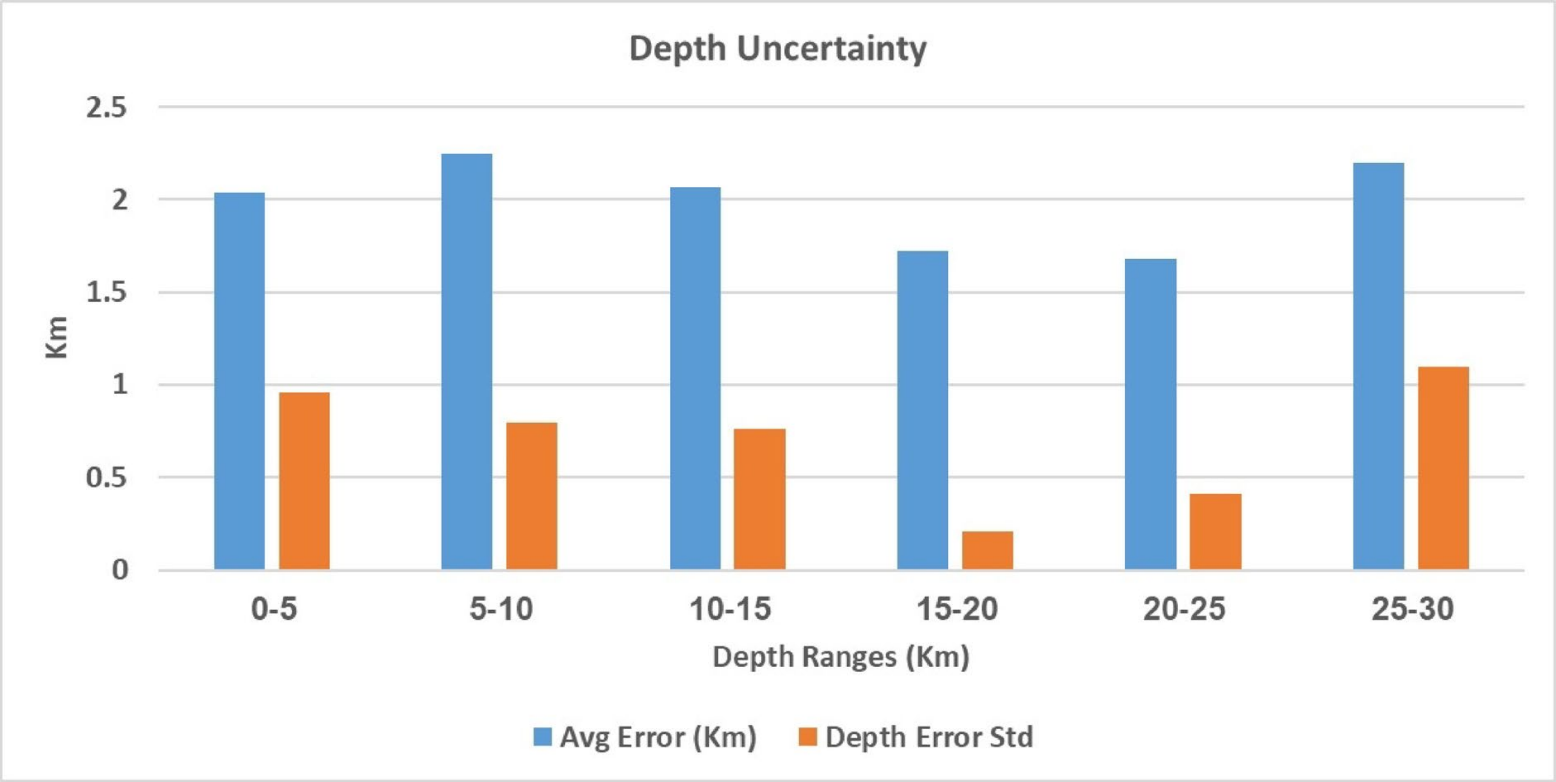
Histogram illustrating the uncertainty clarifying error across all depths considered.

Seismicity maps produced in this study (Fig. 1A, B), based on the relocation of 2,333 earthquakes recorded in 2004 (histogram in Fig. 2), reveal a clear NE–SW alignment of seismic events. This trend is nearly perpendicular to the Red Sea axis and coincides with a major fault system (Najd Fault System). The majority of relocated earthquakes are concentrated in the southern part of the region, occurring at shallow depths between 0 and 20 km, with magnitudes ranging from 0.7 to 3.8. These events were detected by ten temporary seismic stations deployed during the study. The relocation results demonstrate high accuracy, with RMS residuals ranging from 0.01 to 0.4, horizontal errors between 0.02 and 2 km, and vertical depth errors between 0.05 and 1.0 km as shown in Fig. 3. The predominance of swarm like seismicity highlights the unique geological characteristics of Abu Dabbab, setting it apart from other seismically active regions in Egypt.

## Focal mechanism solution in Abu Dabbab seismic zone

In this study, fault plane solutions were determined for 408 earthquakes in the Abu Dabbab region, with local magnitudes (Ml) ranging from 0.7 to 3.0. Digital waveform data were acquired from a temporary local seismic network installed in the area. Focal mechanisms were constructed using P-wave first motion polarities, with events carefully relocated to enhance the accuracy of azimuth, incidence angle, and polarity readings. This refinement enabled more reliable use of key waveform parameters essential for fault-plane analysis. The classical polarity-based method was applied using PMAN software^23^ to derive the fault plane solutions.

**Fig. 4:**
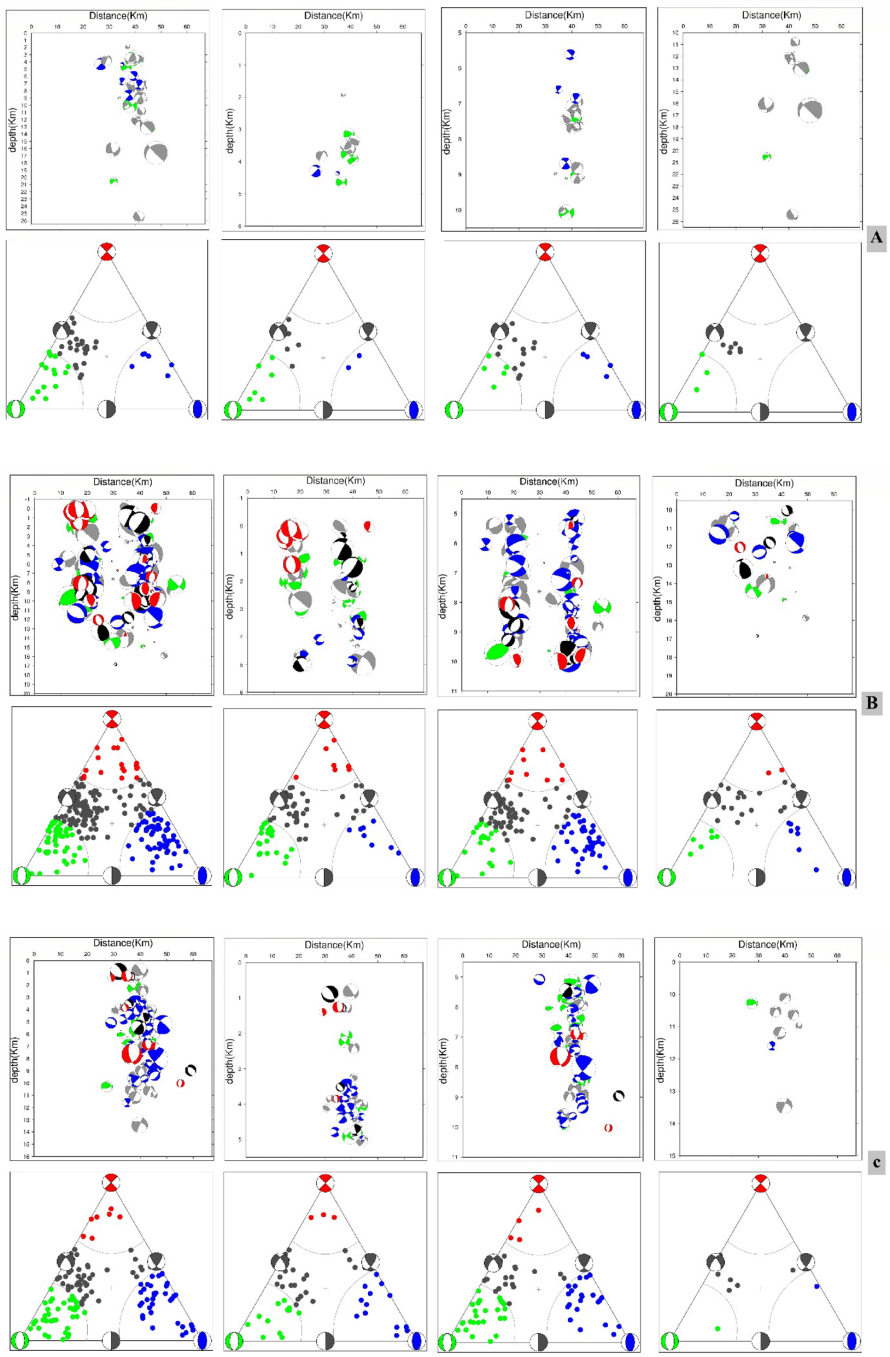
Focal mechanism solutions for the Abu Dabbab area. Solutions are displayed separately by month, and as cross-sectionsfor three main depth intervals: (A) 0–5 km, (B) 5–10 km, and (C) 10–20 km.

To visualize faulting styles, we applied^37^, which plot focal mechanisms within a triangular framework defined by three end member fault types: pure strike-slip, normal, and thrust faulting^37–39^. This approach provides a clear and intuitive framework for comparing the relative distribution of faulting styles within the dataset. Since earthquake activity was concentrated between May and July, focal mechanism solutions were developed independently for each month to capture temporal variations. Cross sectional analysis revealed that seismic events were distributed across three main depth intervals: 0–5 km, 5–10 km, and 10–20 km, offering insights into the vertical segmentation of faulting activity (Fig. 4A, B, C).

**Fig. 5:**
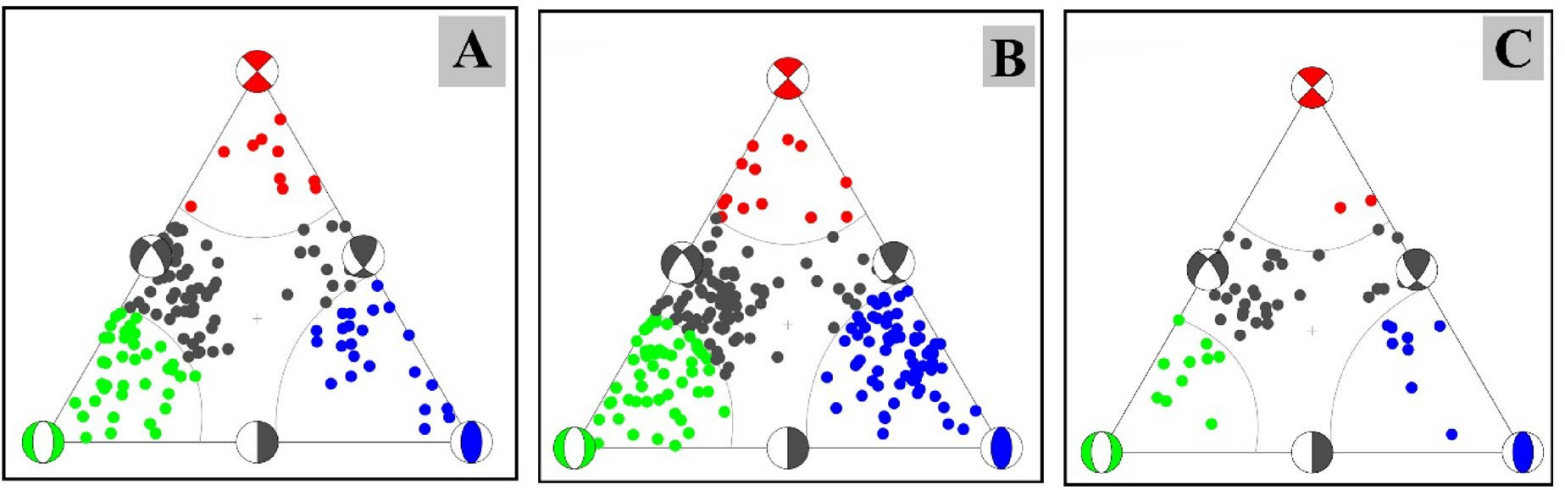
Ternary diagram illustrating the distribution of focal mechanism types for earthquakes in the Abu Dabbab area, classified bydepth. The diagram compares (A) shallow (0–5 km), (B) intermediate (5–10 km), and (C) deeper (10–20 km) events.

**Fig. 6:**
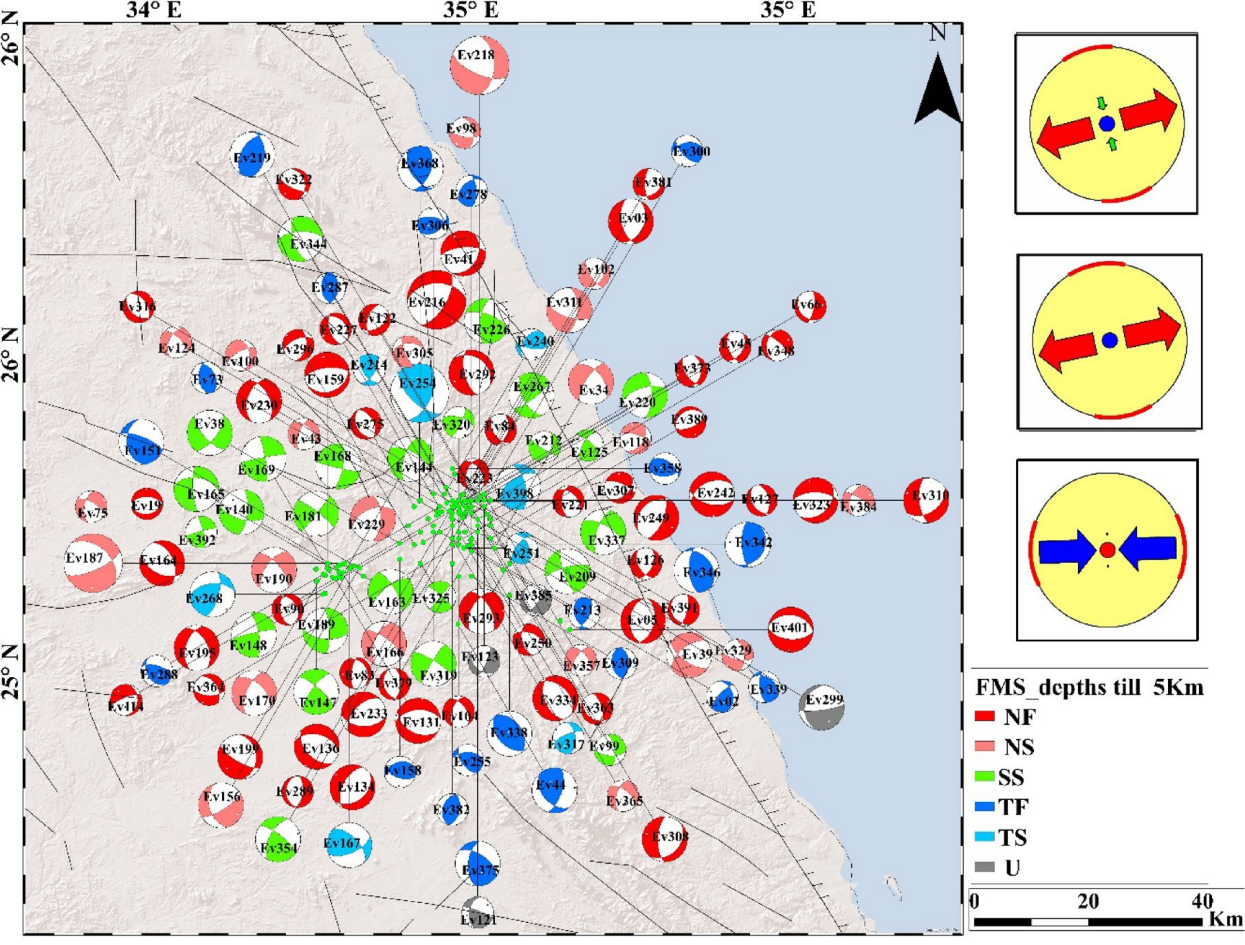
Geographical distribution of selected earthquakes in the Abu Dabbab area with focal mechanism (beach-ball)representations at shallow depths (0–5 km). The right panel illustrates the dominant stress regime at this depth.

**Fig. 7:**
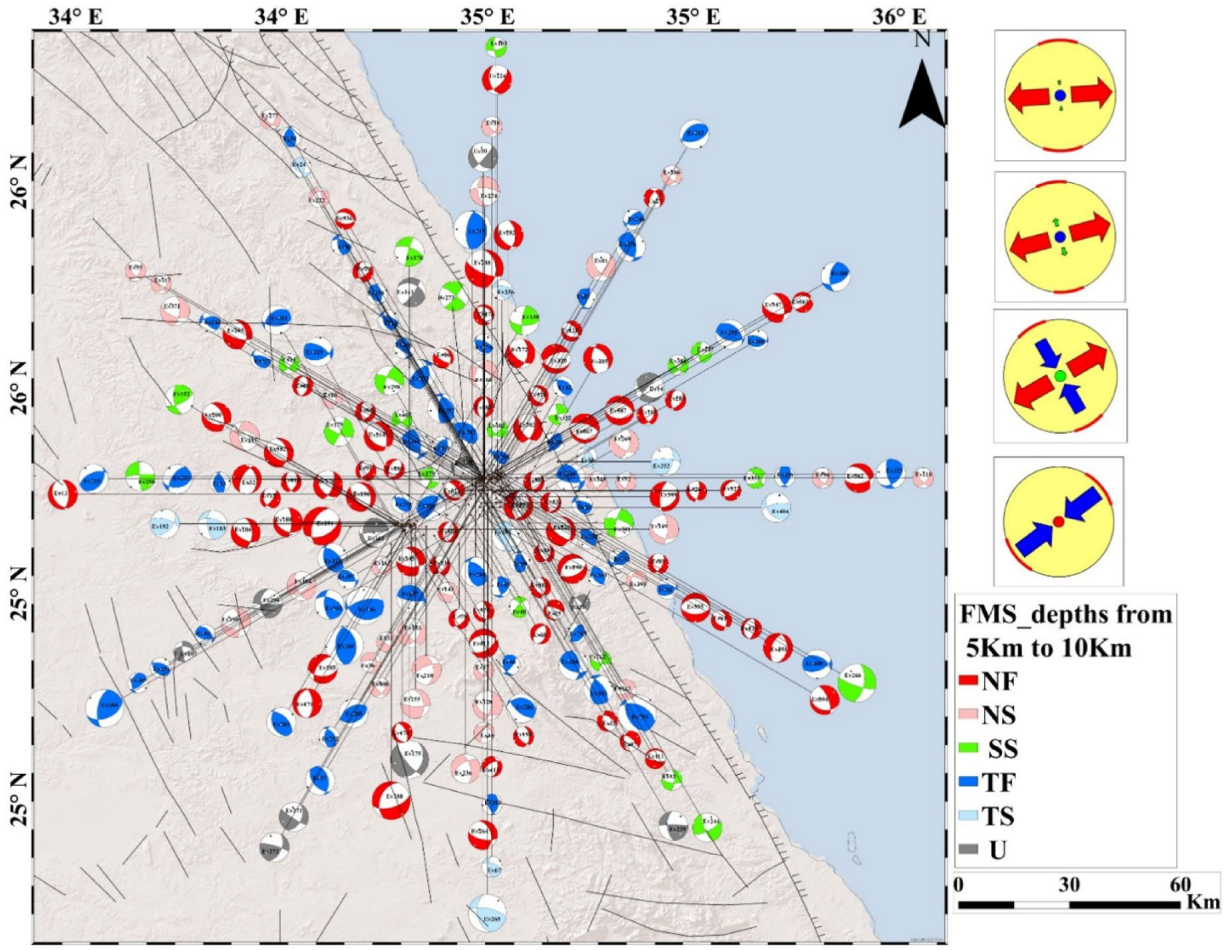
Geographical distribution of selected earthquakes in the Abu Dabbab area with focal mechanism (beach-ball)representations at intermediate depths (5–10 km). The right panel illustrates the dominant stress regime at this depth.

**Fig. 8:**
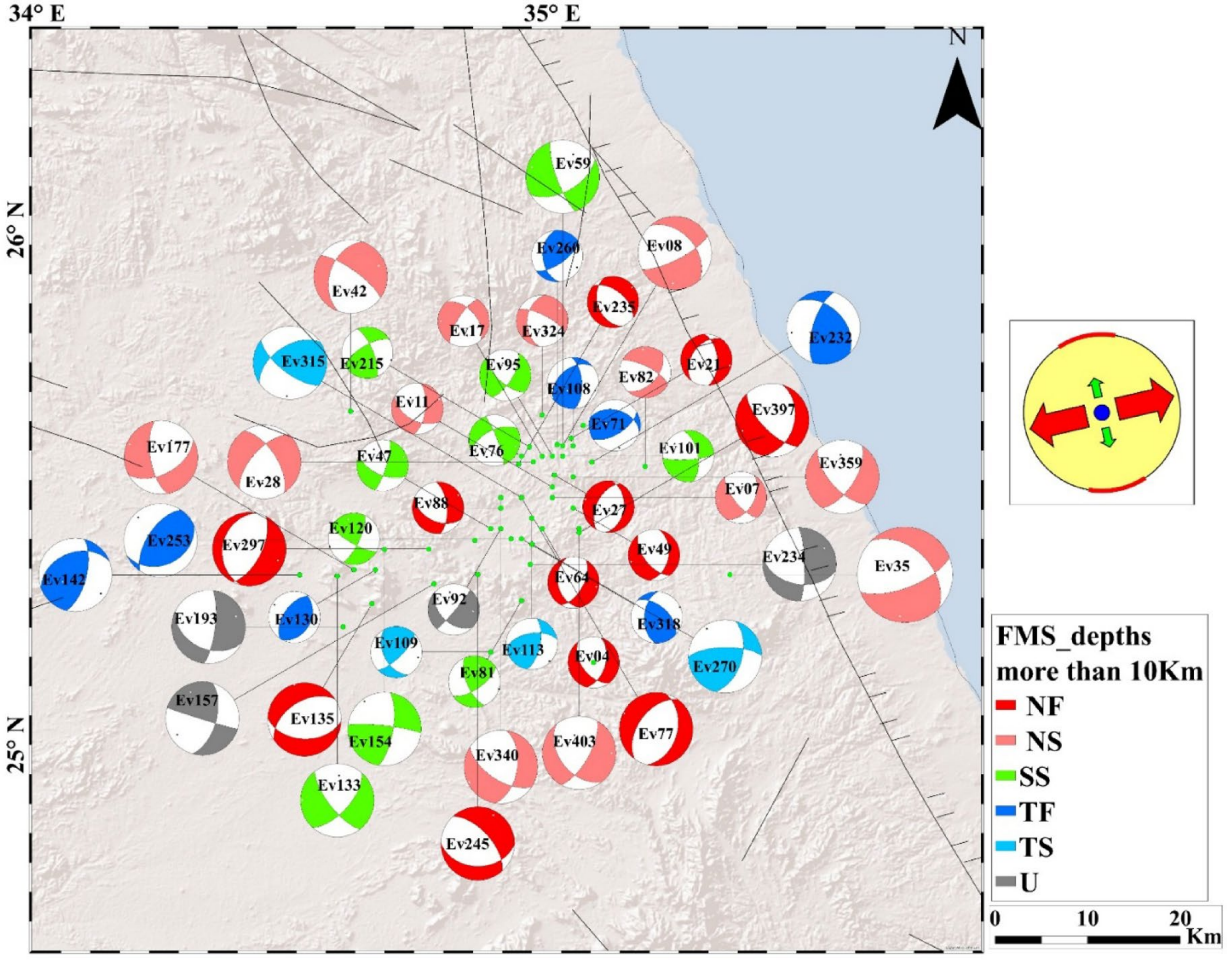
Geographical distribution of selected earthquakes in the Abu Dabbab area with focal mechanism (beach-ball)representations at deeper depths (more than 10 km). The right panel illustrates the dominant stress regime at this depth.

The analysis of 408 focal mechanism solutions reveals a wide range of faulting styles in the Abu Dabbab region, including normal, strike slip, reverse, and oblique faulting. These variations are effectively illustrated using ternary plots (Fig. 5A, B, C). Example of hypocentral parameters for selected events are presented in Table 1, with corresponding focal mechanism solutions in Table 2. The complete hypocentral dataset is provided in the supplementary material. Figures 6, 7, and 8 illustrate the spatial distribution of seismic events across the Abu Dabbab region, accompanied by focal mechanism “beach ball” diagrams and detailed beach balls with stations distribution are provided in the supplementary material.

**Table 1:** Example of hypocentral parameters for selected earthquakes used in constructing focal mechanism solution in Abu Dabbab area.

Event_no	year	mon	day	hour	min	sec	long	lat	depth	Ml
Ev01	2004	5	21	0	5	0	34.47	25.27	7.28	1
Ev02	2004	5	21	0	13	0	34.45	25.22	4.36	0.4
Ev03	2004	5	21	2	9	0	34.49	25.26	3.94	1
Ev04	2004	5	21	2	28	0	34.54	25.08	22	0.7
Ev05	2004	5	21	12	33	0	34.64	25.08	3.15	1
Ev06	2004	5	21	16	28	0	34.5	25.24	12.07	0.8
Ev07	2004	5	21	17	15	0	34.51	25.29	12.76	1.3
Ev08	2004	5	21	18	43	0	34.47	25.26	5.62	0.9
Ev09	2004	5	21	20	13	0	34.48	25.27	6.73	0.7
Ev10	2004	5	21	20	54	0	34.5	25.25	12.31	0.7

**Table 2:** Example of focal mechanism parameters for selected earthquakes in Abu Dabbab area.

Event_no	strike1	dip1	rake1	strike2	dip2	rake2	P_axis		T_axis	
							Az	Pl	Az	Pl
Ev01	143	62	− 149	37	63	− 32	359	41	90	1
Ev02	344	33	36	222	72	118	292	22	167	55
Ev03	23	40	− 61	167	55	− 113	25	70	273	8
Ev04	16	52	− 51	144	52	− 128	350	61	260	0
Ev05	13	49	− 84	183	41	− 97	330	84	99	4
Ev06	31	48	− 29	141	69	− 134	5	47	262	13
Ev07	63	75	− 133	318	45	− 21	292	43	176	18
Ev08	331	49	61	191	49	119	261	0	171	68
Ev09	22	46	− 61	163	51	− 116	10	69	272	3
Ev10	7	64	− 44	120	52	− 146	328	49	66	7

### Results of focal mechanism solutions of earthquakes in Abu Dabbab

Comparative analysis across depth intervals reveals distinct variations in faulting styles, reflecting the region’s complex and layered tectonic structure. In the shallow depth range (0–5 km), most earthquakes exhibit normal, normal oblique, and strike slip mechanisms, with reverse faulting playing only a minor role. At intermediate depths (5–10 km), faulting styles shift noticeably, with reverse and oblique mechanisms becoming more dominant and a decline in strike slip activity. Beyond 10 km, faulting styles are more evenly distributed among all categories, indicating a broader range of tectonic processes at greater depths. Overall, the Abu Dabbab region is shaped by NW–SE and NE–SW trending fault systems, with strike slip components exhibiting depth dependent variability. The constructed focal mechanism solutions serve as the foundation for a comprehensive stress tensor inversion, offering valuable insights into the region’s active deformation and prevailing stress regimes. These results are consistent with the broader tectonic stress orientation and suggest that the observed seismicity is driven by stress concentrations near the tip of a propagating magmatic intrusion^15,40,41^.

## Stress tensor inversion in Abu Dabbab region

To assess the stress field in the Abu Dabbab region, we applied stress tensor inversion to a dataset of 408 focal mechanism solutions. The inversion was performed using the method developed by^24^ implemented through the TENSOR software. Stress inversion techniques are widely recognized for their effectiveness in estimating regional stress fields from earthquake focal mechanisms^26–29^. As mentioned before, the reduced stress tensor is defined by four key parameters: the orientations of the principal stress axes (σ₁, σ₂, σ₃) and the stress ratio (R), which determines the geometry of fault slip^32^. In this study, the dataset was divided into three depth intervals, consistent with the classification used in the focal mechanism analysis, to explore depth dependent variations in the regional stress field.

### Results of stress tensor inversion in Abu Dabbab

#### At shallow depth (0–5 km)

**Fig. 9:**
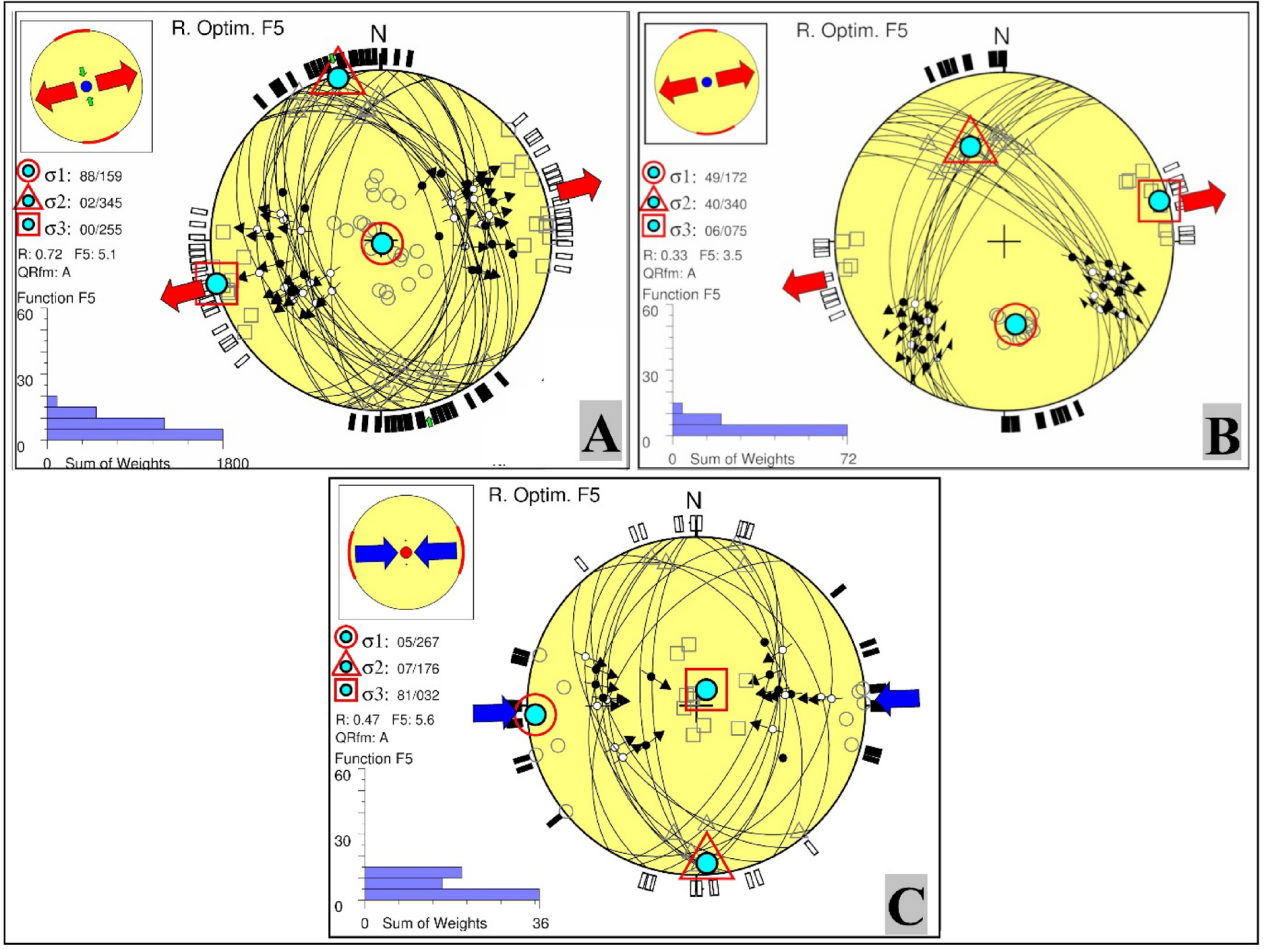
Stress tensor inversion results derived from earthquake focal mechanisms in the Abu Dabbab area at shallow depths (0–5km). (A) Normal faulting regime. (B) Strike-slip regime. (C) Thrust (reverse) regime.

- Based on the stress tensor inversion model developed for the Abu Dabbab region, three distinct stress regimes were identified (Fig. 9):
- Normal Faulting Regime (Fig. 9A): Characterized by a nearly vertical σ_1_ (plunge 88°) and a horizontal σ_3_ (plunge 0°), with a stress ratio R′ equal to R. This configuration indicates an extensional stress field, with the minimum horizontal stress (Sh_min_) oriented N75°E. The solution quality is high (Grade A), supported by a low misfit angle (α = 10.3) and an average misfit function value of F5 = 5.1 (Table 3).
- Oblique (Normal Strike-Slip) Regime (Fig. 9B): Features moderately dipping σ_1_ and σ_2_ (plunges of 49° and 40°, respectively), with a horizontal σ_3_ (plunge 6°), and a stress ratio of R′ = 1.0. This configuration indicates an extensional stress field with a strike slip component, where the minimum horizontal stress (Sh_min_) trends N78°E. The solution is of high quality (Grade A), supported by a low misfit angle (α = 8.4) and an average misfit function value of F5 = 3.5 (Table 3).
- Thrust Faulting Regime (Fig. 9C): Characterized by a horizontal maximum stress axis (σ_1_) and a nearly vertical minimum stress axis (σ_3_), indicating a compressional stress field. The stress ratio is R′ = 2.5, and the solution is of high quality (Grade A), with a misfit angle of 10.1 and an average misfit function of 5.6 (Table 3).

**Table 3:** The results of stress tensor inversion in Abu Dabbab area.

σ_1_		σ_2_		σ_3_		R	α	R`	F5	quality	SH_max_	Sh_min_	Stress regime
Az	Pl	Az	Pl	Az	Pl								
At shallow depth (0–5 km)													
( group_A)													
159	88	345	02	255	00	0.72	10.3	0.72	5.1	A	165	N75°E	NF
( group_B)													
172	49	340	40	75	06	0.33	8.4	1.0	3.5	A	168	N78°E	NS
(group_C)													
267	05	176	07	32	81	0.47	10.1	2.5	5.6	A	88	178°	TF

σ_1_		σ_2_		σ_3_		R	α	R`	F5	quality	SH_max_	Sh_min_	Stress regime
Az	Pl	Az	Pl	Az	Pl								
Intermediate depths (5–10 km)													
(group_A)													
350	78	176	12	86	01	0.45	11.4	0.45	5.9	A	176	86°	NF
(group_B)													
348	53	162	37	254	03	0.4	9.5	1	4.5	A	165	N75°E	NS
(group_C)													
330	06	163	84	61	1	0.75	7.9	1.5	3.2	B	150	60°	SS
(group_D)													
53	00	323	17	143	73	0.5	10.6	2.5	4.8	A	53	143°	TF

σ_1_		σ_2_		σ_3_		R	α	R`	F5	quality	SH_max_	Sh_min_	Stress regime
Az	Pl	Az	Pl	Az	Pl								
At deeper depths (more than 10 km)													
346	55	168	35	78	01	0.33	9	01	3.8	A	167	N77°E	NS

#### At intermediate depth (5–10 km)

**Fig. 10:**
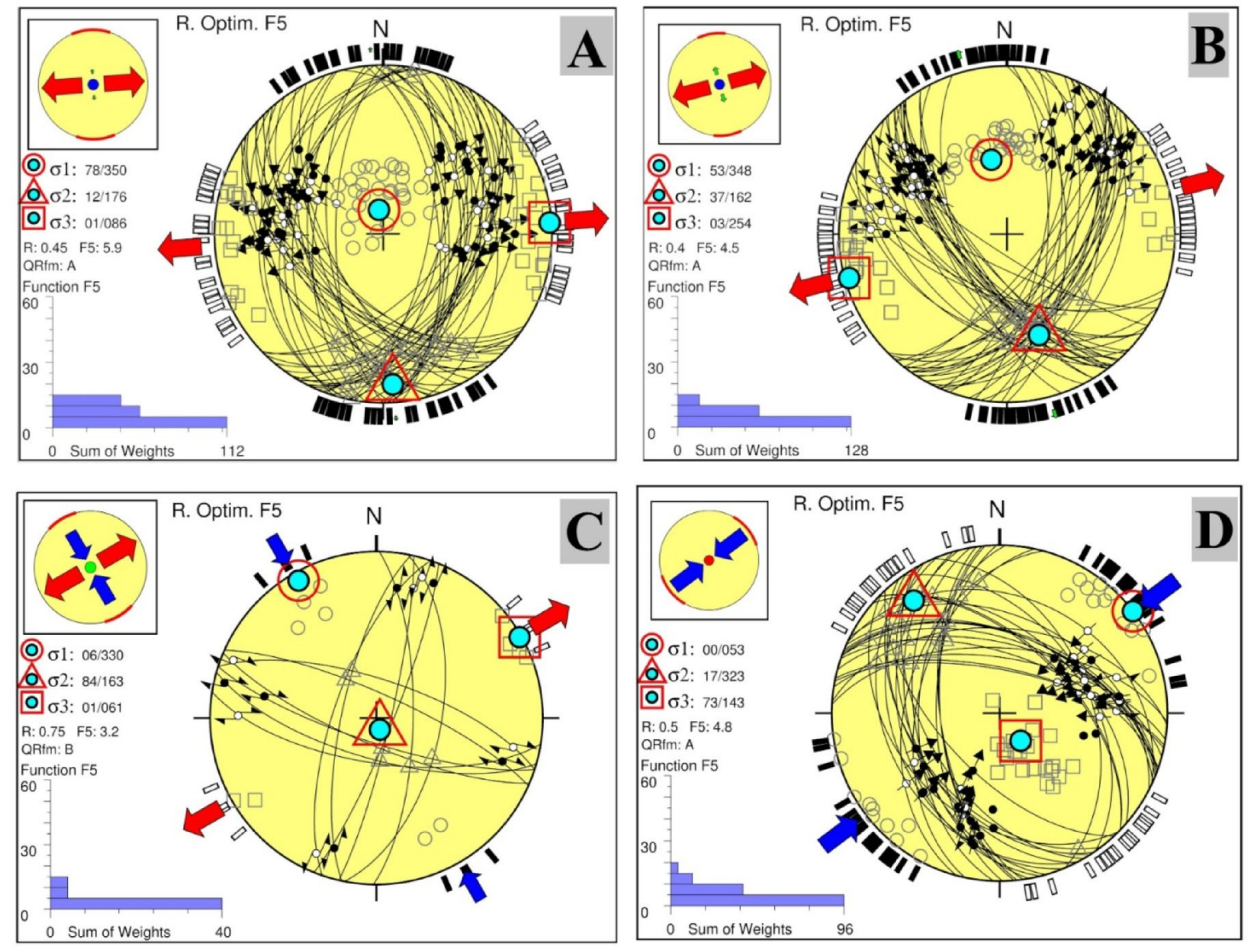
Stress tensor inversion results derived from earthquake focal mechanisms in the Abu Dabbab area at intermediate depths(5-10 km). (A) Normal faulting regime. (B) Normal-Strike regime. (C) Thrust (reverse) regime.

Stress tensor inversion for the region revealed four distinct stress types, as shown in Fig. 10:

- Normal Faulting (Fig. 10A): Characterized by a sub-vertical maximum principal stress axis (σ_1_, plunge 78°) and a horizontal minimum principal stress axis (σ_3_, plunge 1°), with a stress ratio of R′ = R. This configuration indicates an extensional stress field, with the minimum horizontal stress (Sh_min_) trending N86°E. The solution is of high quality (Grade A), supported by a misfit angle (α = 11.4) and an average misfit function value of F5 = 5.9 (Table 3).
- Oblique (Normal Strike-Slip) Regime (Fig. 10B): Features moderately dipping σ_1_ and σ_2_ (plunges of 53° and 37°, respectively), with a horizontal σ_3_ (plunge 3°), and a stress ratio of R′ = 1. This configuration indicates an extensional stress field with a strike slip component, where the minimum horizontal stress (Sh_min_) trends N75°E. The solution is of high quality (Grade A), supported by a low misfit angle (α = 9.7) and an average misfit function value of F5 = 4.5 (Table 3).
- Strike-Slip Regime (Fig. 10C): In this regime, the maximum (σ_1_) and minimum (σ_3_) principal stresses are both sub-horizontal (with plunges of 06° and 01°, respectively), while the intermediate principal stress (σ_2_) is nearly vertical (Table 3, Fig. 9C). The related stress tensor is of B quality, featuring a low misfit angle (α) of 7.9° and an average misfit function (F5) of 3.2. This regime is characterized by an extensional direction of N60°E and a stress ratio (R′) of 1.5.
- Thrust Faulting Regime (Fig. 10D): Characterized by a horizontal maximum principal stress axis (σ_1_, plunge 0°), a nearly vertical minimum stress axis (σ_3_, plunge 73°), and a sub-horizontal intermediate axis (σ_2_). This configuration indicates a compressional stress field, with a stress ratio of R′ = 2.50. The solution is rated high quality (Grade A), supported by a misfit angle of 10.6 and an average misfit function value of F5 = 4.8 (Table 3).

#### At depth greater than 10 km

**Fig. 11:**
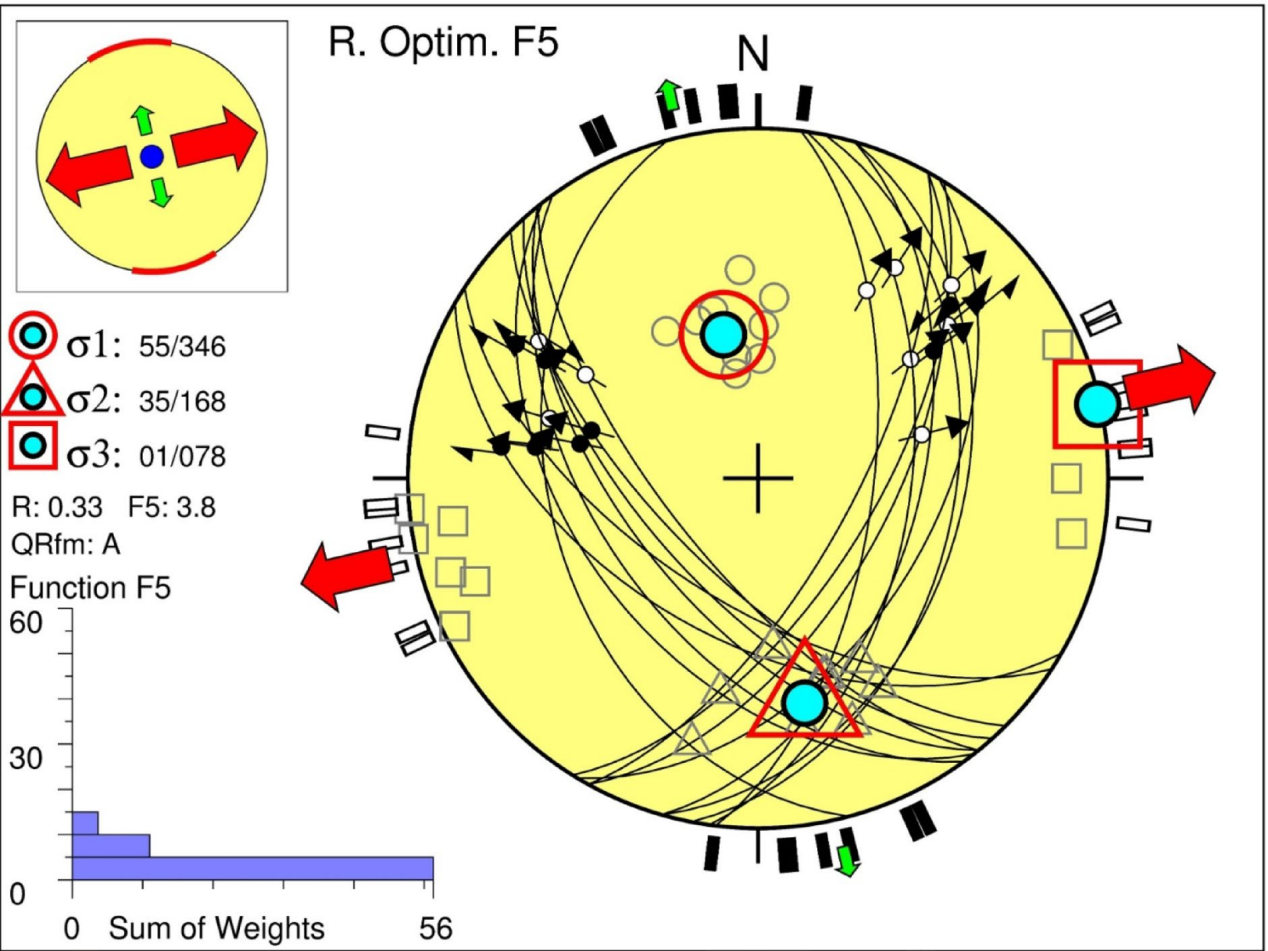
Stress tensor inversion results derived from earthquake focal mechanisms in the Abu Dabbab area at deeper depth (morethan 10 km).

For seismic events deeper than 10 km, the optimal stress tensor inversion indicates an oblique tectonic regime characterized by normal and strike slip faulting components (Fig. 11). This regime features a sub vertical σ₁ (plunge 55°) and a horizontal σ₃ (plunge 1°), with a stress ratio R′ = 1. The resulting oblique normal faulting is oriented with a minimum horizontal stress (Sh_min_) direction of N77°E. This high quality, Grade A solution is constrained by a low misfit angle (α = 9°) and an average misfit function value (F5) of 3.8 (Table 3).

The results demonstrate a complex stress history in the Abu Dabbab region, with evidence for normal, strike slip, thrust, and oblique faulting regimes across different depths. Normal faulting dominates at shallow and deep levels, while intermediate depths reveal a more varied stress field, reflecting transitional tectonic processes. This pattern can be interpreted in the context of dike intrusion induced stresses: compression in the walls of a dike and tension above a propagating dike tip, with hypocenters potentially migrating ahead of the advancing dike front.

## Discussion

This research delivers a depth resolved analysis of stress and deformation within the Abu Dabbab seismic zone. By integrating focal mechanism solutions with stress tensor inversion across multiple crustal levels, the study advances beyond simple tectonic classification. The findings reveal a vertically layered seismogenic system governed by the combined influence of regional tectonic forces and localized magmatic processes.

The key finding of this study is the distinct vertical partitioning of faulting styles and stress regimes, which together define a coherent mechanical model of crustal deformation beneath Abu Dabbab. In the shallow crust (0–5 km), deformation is dominated by normal and strike slip faulting under normal and oblique normal stress regimes. This pattern reflects the regional Red Sea extensional tectonics, driven by SE–NW extension. However, the recurring presence of a localized thrust stress regime at these depths indicates a notable disturbance within the regional stress field.

**Fig. 12:**
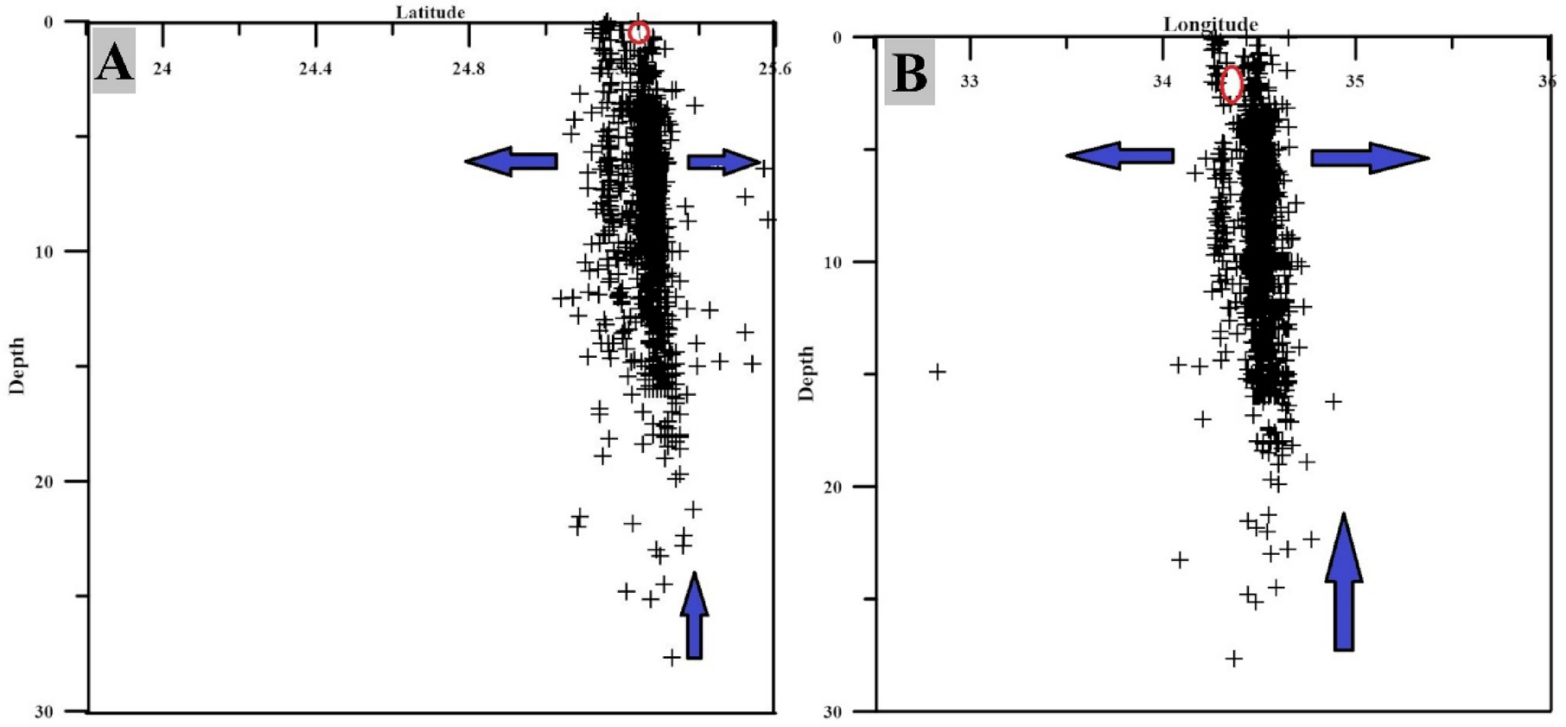
Cross section illustrating the distribution of the earthquakes (seismicity) in the area of Abu Dabbab with depth. A: latitudeversus depth profile. B: longitude versus depth profile.

The shallow compressional pocket is best explained by stress rotation or clamping, resulting from the mechanical interaction between the extending crust and a shallow, rigid intrusive body, or alternatively from the complex geometry of intersecting and reactivated Najd fault systems. At depths of 5–10 km, the crust becomes a pronounced stress transition zone marked by strong heterogeneity. Within this interval, all four modeled stress regimes normal, oblique, strike slip, and thrust are present, indicating that the crust is subject to competing and spatially variable forces rather than a uniform stress field. This depth range is interpreted as the key interaction zone where regional extensional stresses from the Red Sea rift intersect with localized perturbations driven by an actively pressurizing magmatic intrusion. The lateral and vertical migration of magma can impose compressional stresses along intrusion flanks and generate transient compression ahead of its advancing tip (Fig. 12), while regional extension continues to dominate the surrounding crust. The coexistence of reverse and normal faulting within this interval a feature widely reported in magmatically active rift settings serves as a diagnostic marker of dynamic tectonic magmatic interplay. At depths greater than 10 km, the stress field becomes more homogeneous, with deformation primarily governed by oblique normal faulting.

This pattern indicates that the influence of local magmatic perturbations and near surface crustal heterogeneity diminishes with depth, leaving deformation at greater levels primarily governed by a stable regional extensional stress regime. Such deep seated extension is consistent with large scale crustal necking beneath the Red Sea margin and likely represents the fundamental tectonic driver into which the magmatic system is emplaced.

Our depth dependent analysis strongly supports a hybrid model that resolves the longstanding debate over the source of Abu Dabbab’s seismicity. Regional NE–SW compression and SE–NW extension establish the tectonic framework, orienting the principal stress axes and favoring slip along two dominant fault sets: NW–SE trending faults (normal to oblique slip) and NE–SW trending faults (strike slip).

This explains the consistent fault orientations and the broader regional kinematic framework. However, the anomalous features such as intense swarm like clustering, elevated heat flow (~ 92 mW/m^2^), the coexistence of contradictory stress regimes in close proximity, and reports of seismic sounds cannot be attributed to regional tectonics alone. Our model proposes that an actively pressurizing magmatic intrusion is the key local driver of Abu Dabbab’s seismicity. Acting as a stress concentrator, it amplifies and rotates stresses, producing the heterogeneous regimes observed. The seismicity pattern particularly the migration and swarm behavior noted in earlier studies is interpreted as the response to dike propagation: hypocenters advance with the dike tip under tensile stresses, while compressive events occur along its flanks. In this framework, regional extension establishes the fractured crust and fundamental stress orientation, while the magmatic intrusion supplies the dynamic energy and localized stress complexity that sustain swarm activity. This interpretation is supported by seismic tomography studies showing velocity anomalies^6^ and is consistent with global analogues of seismicity in magmatically active rift systems. Recognizing a multiphase, depth dependent stress regime has significant implications for seismic hazard assessment in the Abu Dabbab region. Faults are not limited to a single kinematic style; instead, the same NE–SW or NW–SE trending structures may shift between normal, strike-slip, or reverse motion depending on transient local stress perturbations and depth. This variability increases uncertainty in defining seismic source mechanisms and complicates the development of fault based hazard models.

The presence of localized compressional stress regimes indicates that the crust can accumulate and release strain through reverse faulting, meaning hazard assessments must account for the possibility of moderate magnitude earthquakes (M ~ 5–6) driven by non extensional mechanisms. Such events may generate ground motion characteristics distinct from those expected in typical normal faulting earthquakes within extensional settings. Furthermore, the spatial clustering of heterogeneous seismicity and swarm activity in the southern sector particularly within the 5–10 km depth range marks this zone as an area of elevated seismic potential that should be prioritized for targeted monitoring and hazard mitigation efforts. The tectonic magmatic framework developed in this study significantly strengthens and de-risks the geothermal potential of the Abu Dabbab region. Seismicity is interpreted as direct evidence of an active mid crustal magmatic heat source, providing a robust explanation for the exceptionally high surface heat flow reported in the area^4,5^. The predominance of normal and oblique faulting within both shallow and deep extensional regimes further indicates that the crust is extensively fractured by ongoing extension. This interconnected fault and fracture network enhances permeability, enabling efficient fluid circulation and heat extraction. Moreover, the persistence of seismic activity over several decades points to a long lived system of heat and fluid transport, a defining characteristic of sustainable geothermal resources.

Collectively, these findings establish Abu Dabbab as a scientifically validated, high priority target for geothermal exploration and development. The study reframes the area from being simply seismically active to an evidence based focal point within Egypt’s renewable energy strategy, highlighting its strong potential for sustainable geothermal resource utilization.

## Conclusions

The study of 408 micro earthquakes in Abu Dabbab, integrating focal mechanism analysis with depth dependent stress tensor inversion, reveals a vertically stratified crust composed of three mechanically distinct layers. Findings from Abu Dabbab highlight structural complexity and stress heterogeneity within its seismotectonic and geothermal framework.

The shallow crust (0–5 km) is mainly shaped by Red Sea related extension, with localized compressional effects arising from rigid intrusions and the complex geometry of reactivated Najd faults.

Intermediate crust (5–10 km): A heterogeneous transition zone where normal, strike-slip, and reverse faulting coexist, reflecting the interplay of regional extension and magmatic intrusions.

The deep crust (> 10 km) is dominated by a relatively uniform oblique normal extensional regime aligned with the broader regional tectonic field, as the influence of near surface heterogeneities and magmatic perturbations diminishes with depth, resulting in a more stable stress environment.

Seismicity in Abu Dabbab is shaped by both regional tectonics and local magmatism. The Red Sea rift imposes NE–SW compression and SE–NW extension, reactivating Najd faults and influencing stress orientations. A mid crustal magmatic intrusion further concentrates stress, driving transient stress rotations, fluid pressure increases, and deformation that sustain swarm activity.

In volcanic and geothermal environments, brittle failure can involve the sudden opening of tensile cracks, producing non double couple radiation patterns^18,19^. This dual control mechanism explains the clustered distribution of seismicity and its depth migration, while also accounting for the anomalously high heat flow and the coexistence of contrasting stress regimes in close proximity.

In the study area, a multiphasic stress environment complicates seismic hazard assessment. Faults trending NE–SW or NW–SE may shift between normal, strike slip, or reverse slip depending on depth and local stress perturbations. This variability increases uncertainty in seismic source characterization, making hazard evaluation more complex.

In Abu Dabbab, the southern zone at intermediate depths shows concentrated, heterogeneous seismicity, marking it as an area of elevated seismic potential that should be prioritized for hazard monitoring. At the same time, the region demonstrates strong geothermal potential, underscoring its dual importance for both risk assessment and energy exploration.

In Abu Dabbab, persistent seismicity indicates an active mid crustal magmatic heat source, while widespread normal and oblique faulting reveals a fractured crust that enables fluid circulation. Together, these conditions create a favorable setting for geothermal resource development. It also combines sustained heat, pervasive permeability, and long lived fluid transport, making it an exceptionally promising site for geothermal exploration and sustainable energy development.

Analysis of 408 microearthquakes in the Abu Dabbab area reveals a complex stress field characterized by normal, strike-slip, and reverse faulting across different depth ranges. The coexistence of compressional and extensional regimes reflects the interaction between NE–SW compression and SE–NW extension associated with the broader Red Sea rift system, in agreement with previous studies. Recurrent swarms of shallow to intermediate depth seismicity delineate zones of stress concentration along active faults, providing critical insights for assessing future earthquake potential and understanding localized stress perturbations that may trigger seismic events.

The predominance of normal and oblique slip faulting in Abu Dabbab enhances crustal permeability, facilitating fluid circulation and heat transport, which are key factors for geothermal exploration. This study underscores the value of high precision earthquake relocation, focal mechanism analysis, and stress tensor inversion in rift environments. Integrating seismic observations with geophysical and geochemical data further constrains the roles of magmatic intrusions and crustal heterogeneity in controlling stress evolution and seismicity, thereby improving our understanding of the tectono magmatic processes governing Abu Dabbab’s seismic behavior.

## Implications

The analyses of 407 microearthquakes in the Abu Dabbab region provide important insights into the seismotectonic and geodynamic behavior of this part of the Eastern Desert. The identification of multiple faulting mechanisms normal, strike-slip, and reverse across different depth ranges highlights the complexity of the local stress field and the coexistence of compressional and extensional regimes. These findings are consistent with broader Red Sea rift related tectonics and reflect the interplay between NE–SW compression and SE–NW extension observed in previous studies.

From a seismic hazard perspective, the shallow and intermediate depth seismicity, particularly the recurrent swarms, points to areas of potential stress concentration along active faults. Such information is critical for assessing the likelihood of future earthquake activity and understanding localized stress perturbations that could trigger seismic events.

The observed faulting patterns also have implications for geothermal energy exploration. The predominance of normal and oblique slip faults, coupled with evidence of extensional structures, suggests enhanced crustal permeability that could facilitate fluid circulation and heat transport. Finally, the study underscores the importance of high resolution earthquake relocation, focal mechanism determination, and stress tensor inversion in rift environments. Integrating these seismic observations with geophysical and geochemical datasets can further constrain the influence of magmatic intrusions and heterogeneous crustal structures on local stress evolution and seismicity. Overall, the findings contribute to a more comprehensive understanding of the interplay between tectonic and magmatic processes in shaping Abu Dabbab’s seismic dynamics.

## Supplementary Information

Below is the link to the electronic supplementary material.

Supplementary Material 1

## Data Availability

The datasets used and/or analysed during the current study are available from the corresponding author upon reasonable request.
