# Supplementary material for: Updated seismotectonic framework of Abu Dabbab Egypt based on focal mechanisms and stress inversion
Source: Sci Rep. 2026 Feb 14;16:6527. doi: 10.1038/s41598-026-36922-3 (PMC12910059; doi:10.1038/s41598-026-36922-3)
Supplement: Supplementary file 1 — Supplementary Material 1 [file 41598_2026_36922_MOESM1_ESM.doc]

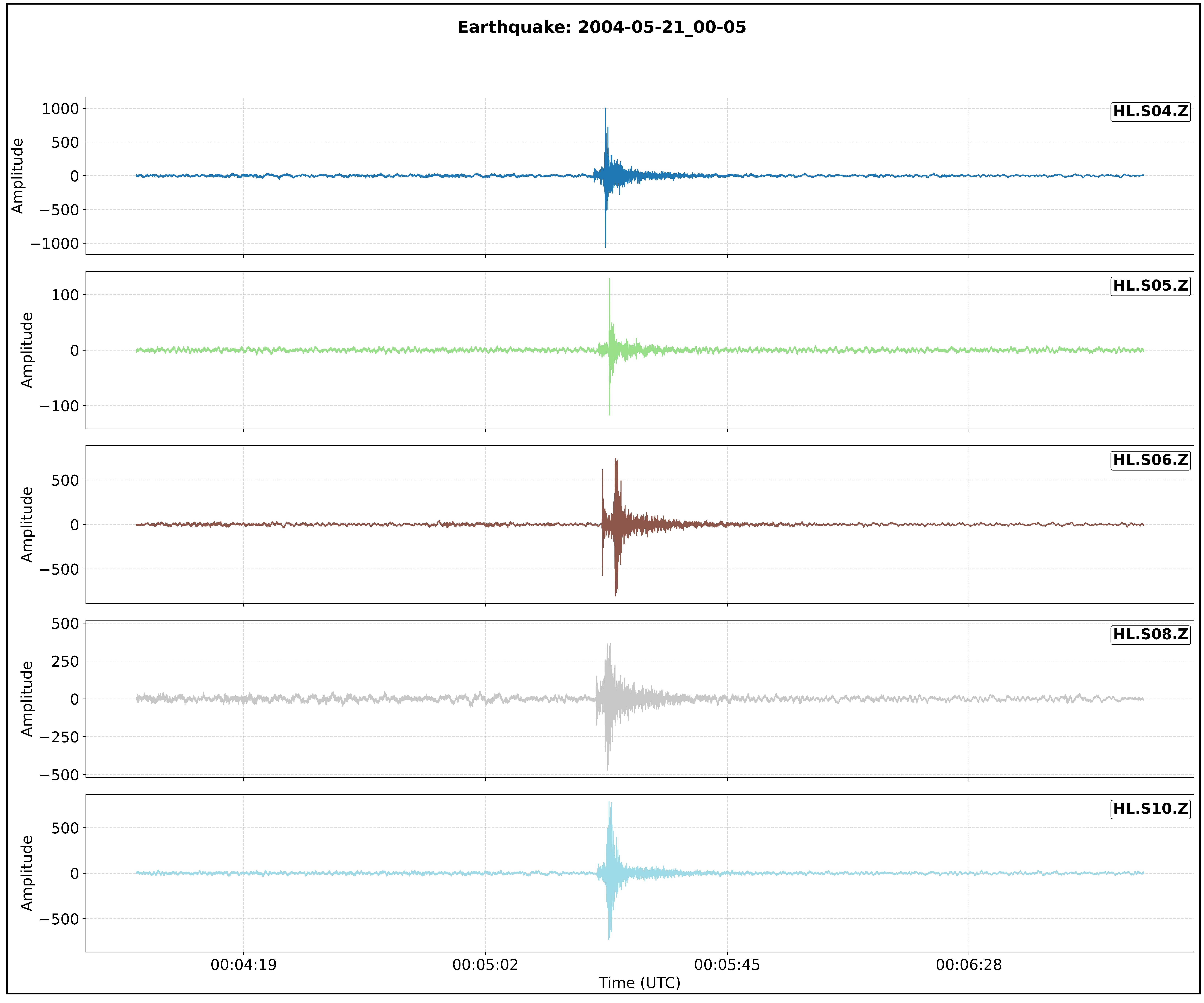


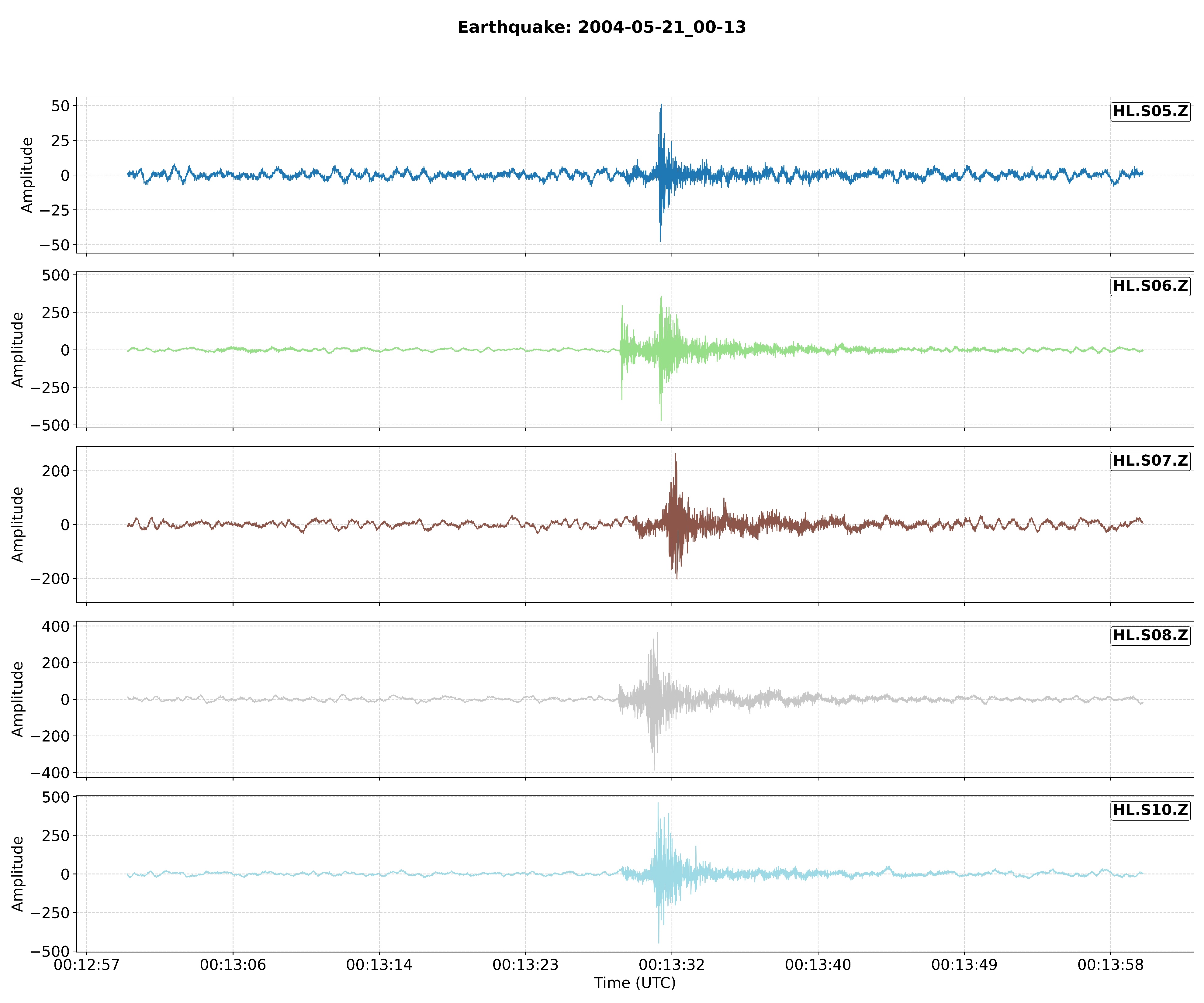


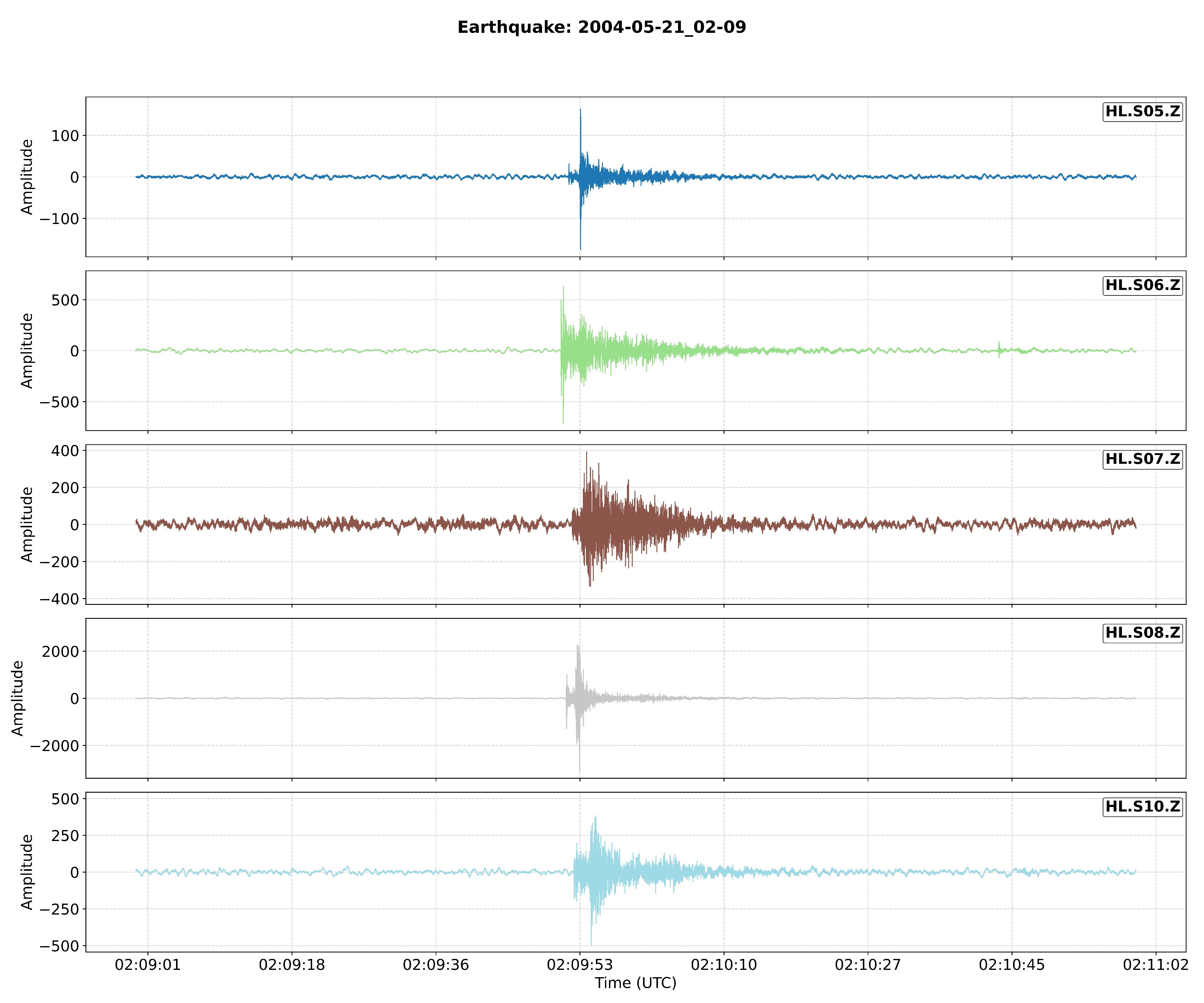


Figure 1: Waveform Samples of some earthquakes in Abu Dabbab region

Table1: hypocentral parameters for all earthquakes used in constructing focal mechanism solutions in Abu Dabbab area

| **Ev_no** | **year** | **mon** | **day** | **hour** | **min** | **sec** | **long** | **lat** | **depth** | **mag** |
| --- | --- | --- | --- | --- | --- | --- | --- | --- | --- | --- |
| **Ev01** | 2004 | 5 | 21 | 0 | 5 | 0 | 34.47 | 25.27 | 7.28 | 1 |
| **Ev02** | 2004 | 5 | 21 | 0 | 13 | 0 | 34.45 | 25.22 | 4.36 | 0.4 |
| **Ev03** | 2004 | 5 | 21 | 2 | 9 | 0 | 34.49 | 25.26 | 3.94 | 1 |
| **Ev04** | 2004 | 5 | 21 | 2 | 28 | 0 | 34.54 | 25.08 | 22 | 0.7 |
| **Ev05** | 2004 | 5 | 21 | 12 | 33 | 0 | 34.64 | 25.08 | 3.15 | 1 |
| **Ev06** | 2004 | 5 | 21 | 13 | 17 | 26.66 | 34.3915 | 25.266 | 8.17 | 0.3 |
| **Ev07** | 2004 | 5 | 21 | 16 | 28 | 0 | 34.5 | 25.24 | 12.07 | 0.8 |
| **Ev08** | 2004 | 5 | 21 | 17 | 15 | 0 | 34.51 | 25.29 | 12.76 | 1.3 |
| **Ev09** | 2004 | 5 | 21 | 18 | 43 | 0 | 34.47 | 25.26 | 5.62 | 0.9 |
| **Ev010** | 2004 | 5 | 21 | 20 | 13 | 0 | 34.48 | 25.27 | 6.73 | 0.7 |
| **Ev11** | 2004 | 5 | 21 | 20 | 54 | 0 | 34.5 | 25.25 | 12.31 | 0.7 |
| **Ev12** | 2004 | 5 | 22 | 2 | 40 | 0 | 34.47 | 25.24 | 8.94 | 0.2 |
| **Ev13** | 2004 | 5 | 22 | 3 | 4 | 0 | 34.47 | 25.24 | 9.9 | 1.3 |
| **Ev14** | 2004 | 5 | 22 | 16 | 34 | 0 | 34.48 | 25.26 | 6.81 | 1.1 |
| **Ev15** | 2004 | 5 | 22 | 23 | 6 | 0 | 34.46 | 25.23 | 9.86 | 0.6 |
| **Ev16** | 2004 | 5 | 22 | 23 | 39 | 0 | 34.57 | 25.14 | 3.77 | 1 |
| **Ev17** | 2004 | 5 | 23 | 18 | 27 | 0 | 34.49 | 25.28 | 10.46 | 0.8 |
| **Ev18** | 2004 | 5 | 23 | 19 | 6 | 0 | 34.488 | 25.27 | 6.85 | 0.9 |
| **Ev19** | 2004 | 5 | 23 | 20 | 37 | 0 | 34.4764 | 25.2646 | 3.8 | 0.4 |
| **Ev20** | 2004 | 5 | 23 | 21 | 2 | 0 | 34.4866 | 25.283 | 7.34 | 0.4 |
| **Ev21** | 2004 | 5 | 23 | 22 | 20 | 0 | 34.5183 | 25.2973 | 12.82 | 0.9 |
| **Ev22** | 2004 | 5 | 25 | 13 | 36 | 0 | 34.454 | 25.2152 | 6.6 | 0.7 |
| **Ev23** | 2004 | 5 | 25 | 13 | 52 | 0 | 34.5074 | 25.2785 | 8.84 | 0.5 |
| **Ev24** | 2004 | 5 | 25 | 15 | 52 | 0 | 34.4889 | 25.2736 | 9.11 | 0.9 |
| **Ev25** | 2004 | 5 | 25 | 15 | 56 | 0 | 34.4777 | 25.2741 | 6.58 | 0.5 |
| **Ev26** | 2004 | 5 | 25 | 17 | 14 | 0 | 34.5042 | 25.2559 | 8.95 | 1 |
| **Ev27** | 2004 | 5 | 25 | 17 | 16 | 0 | 34.502 | 25.2616 | 11.5 | 0.1 |
| **Ev28** | 2004 | 5 | 25 | 18 | 31 | 0 | 34.4816 | 25.2746 | 25.15 | 1 |
| **Ev29** | 2004 | 5 | 25 | 21 | 12 | 0 | 34.5187 | 25.2476 | 7 | 0.9 |
| **Ev30** | 2004 | 5 | 25 | 22 | 22 | 0 | 34.4885 | 25.2757 | 8.48 | 1 |
| **Ev31** | 2004 | 5 | 27 | 1 | 24 | 0 | 34.4895 | 25.2834 | 7.24 | 0.8 |
| **Ev32** | 2004 | 5 | 27 | 1 | 31 | 0 | 34.4814 | 25.2771 | 7.04 | 1 |
| **Ev33** | 2004 | 5 | 27 | 6 | 56 | 0 | 34.4839 | 25.2749 | 6.91 | 0.9 |
| **Ev34** | 2004 | 5 | 29 | 3 | 26 | 0 | 34.5362 | 25.1946 | 3.61 | 1 |
| **Ev35** | 2004 | 5 | 29 | 4 | 46 | 0 | 34.6723 | 25.1652 | 18.17 | 2.1 |
| **Ev36** | 2004 | 5 | 29 | 17 | 16 | 0 | 34.4596 | 25.2508 | 7.13 | 0.9 |
| **Ev37** | 2004 | 5 | 29 | 18 | 38 | 0 | 34.4849 | 25.2193 | 8.69 | 1 |
| **Ev38** | 2004 | 5 | 30 | 0 | 58 | 0 | 34.3871 | 25.202 | 3.81 | 1 |
| **Ev39** | 2004 | 5 | 30 | 1 | 36 | 0 | 34.4687 | 25.2436 | 3.5 | 1 |
| **Ev40** | 2004 | 5 | 30 | 18 | 11 | 0 | 34.4596 | 25.2312 | 4.63 |  |
| **Ev41** | 2004 | 5 | 30 | 18 | 13 | 0 | 34.4869 | 25.2671 | 3.36 | 1 |
| **Ev42** | 2004 | 5 | 30 | 18 | 26 | 0 | 34.3041 | 25.3237 | 14.4 | 1.3 |
| **Ev43** | 2004 | 5 | 30 | 20 | 8 | 0 | 34.4455 | 25.2527 | 1.92 | 0.4 |
| **Ev44** | 2004 | 5 | 30 | 21 | 59 | 0 | 34.4787 | 25.0747 | 4.29 | 1.1 |
| **Ev45** | 2004 | 5 | 30 | 23 | 7 | 0 | 34.4961 | 25.2674 | 3.92 | 0.5 |
| **Ev46** | 2004 | 5 | 30 | 23 | 8 | 0 | 34.491 | 25.2711 | 7.39 | 0.7 |
| **Ev47** | 2004 | 6 | 8 | 13 | 16 | 20.52 | 34.440 | 25.210 | 24.8 | 0.5 |
| **Ev48** | 2004 | 6 | 8 | 16 | 48 | 3.45 | 34.470 | 25.150 | 6.7 | 0.2 |
| **Ev49** | 2004 | 6 | 8 | 17 | 4 | 10.01 | 34.520 | 25.230 | 14.9 | 0.4 |
| **Ev50** | 2004 | 6 | 8 | 17 | 6 | 56.8 | 34.530 | 25.210 | 8.4 | 0.3 |
| **Ev51** | 2004 | 6 | 8 | 17 | 29 | 45.34 | 34.500 | 25.260 | 3.4 | 1 |
| **Ev52** | 2004 | 6 | 8 | 18 | 46 | 52.76 | 34.500 | 25.270 | 6.3 | 0.2 |
| **Ev53** | 2004 | 6 | 8 | 19 | 0 | 32.95 | 34.490 | 25.270 | 7.9 | 0.1 |
| **Ev54** | 2004 | 6 | 8 | 19 | 12 | 56.74 | 34.510 | 25.270 | 9.6 | 0.1 |
| **Ev55** | 2004 | 6 | 8 | 19 | 18 | 22.18 | 34.510 | 25.280 | 9.9 | 0.3 |
| **Ev56** | 2004 | 6 | 8 | 21 | 1 | 47.17 | 34.480 | 25.260 | 2.1 | 1 |
| **Ev57** | 2004 | 6 | 8 | 23 | 26 | 54.35 | 34.490 | 25.270 | 6.8 | 0.3 |
| **Ev58** | 2004 | 6 | 9 | 0 | 22 | 35.29 | 34.510 | 25.280 | 8.9 | 0.3 |
| **Ev59** | 2004 | 6 | 9 | 1 | 4 | 45.47 | 34.510 | 25.290 | 11.0 | 1 |
| **Ev60** | 2004 | 6 | 9 | 3 | 30 | 0 | 34.491 | 25.273 | 6.3 | 0.1 |
| **Ev61** | 2004 | 6 | 9 | 3 | 35 | 56.57 | 34.490 | 25.270 | 8.0 | 0.3 |
| **Ev62** | 2004 | 6 | 9 | 4 | 44 | 33.96 | 34.530 | 25.240 | 7.2 | 0.3 |
| **Ev63** | 2004 | 6 | 9 | 6 | 33 | 1.04 | 34.490 | 25.270 | 7.6 | 1 |
| **Ev64** | 2004 | 6 | 9 | 8 | 11 | 49.23 | 34.490 | 25.210 | 14.3 | 0.1 |
| **Ev65** | 2004 | 6 | 9 | 8 | 13 | 10.74 | 34.470 | 25.120 | 5.7 | 0.1 |
| **Ev66** | 2004 | 6 | 9 | 9 | 50 | 14.17 | 34.510 | 25.270 | 4.8 | 0.4 |
| **Ev67** | 2004 | 6 | 9 | 10 | 40 | 4.2 | 34.510 | 25.250 | 9.3 | 0.2 |
| **Ev68** | 2004 | 6 | 9 | 11 | 22 | 24.24 | 34.460 | 25.190 | 9.6 | 0.3 |
| **Ev69** | 2004 | 6 | 9 | 15 | 29 | 0.62 | 34.470 | 25.260 | 7.0 | 0.2 |
| **Ev70** | 2004 | 6 | 9 | 20 | 57 | 9.5 | 34.540 | 25.290 | 9.3 | 0.5 |
| **Ev71** | 2004 | 6 | 9 | 21 | 39 | 28.56 | 34.530 | 25.310 | 11.5 | 0.2 |
| **Ev72** | 2004 | 6 | 9 | 21 | 40 | 43.83 | 34.490 | 25.270 | 7.0 | 0.1 |
| **Ev73** | 2004 | 6 | 9 | 23 | 8 | 53.97 | 34.450 | 25.250 | 3.5 | 0.1 |
| **Ev74** | 2004 | 6 | 9 | 23 | 10 | 5.29 | 34.480 | 25.270 | 8.1 | 1 |
| **Ev75** | 2004 | 6 | 10 | 0 | 45 | 52.39 | 34.480 | 25.260 | 4.3 | 0.1 |
| **Ev76** | 2004 | 6 | 10 | 3 | 17 | 43.56 | 34.470 | 25.280 | 10.5 | 0.1 |
| **Ev77** | 2004 | 6 | 10 | 3 | 25 | 38.61 | 34.470 | 25.240 | 10.6 | 1 |
| **Ev78** | 2004 | 6 | 10 | 3 | 30 | 20.48 | 34.500 | 25.270 | 3.6 | 1 |
| **Ev79** | 2004 | 6 | 10 | 3 | 49 | 0.28 | 34.430 | 25.120 | 7.3 | 0.0 |
| **Ev80** | 2004 | 6 | 10 | 4 | 12 | 10.58 | 34.490 | 25.260 | 6.7 | 0.4 |
| **Ev81** | 2004 | 6 | 10 | 9 | 18 | 39.09 | 34.470 | 25.140 | 16.9 | 0.3 |
| **Ev82** | 2004 | 6 | 10 | 14 | 59 | 23.59 | 34.590 | 25.270 | 15.9 | 0.6 |
| **Ev83** | 2004 | 6 | 10 | 15 | 47 | 22.2 | 34.470 | 25.260 | 4.1 | 0.2 |
| **Ev84** | 2004 | 6 | 10 | 16 | 6 | 3.33 | 34.470 | 25.250 | 2.3 | 0.1 |
| **Ev85** | 2004 | 6 | 10 | 21 | 40 | 12.31 | 34.480 | 25.270 | 7.9 | 0.1 |
| **Ev86** | 2004 | 6 | 10 | 23 | 0 | 56.07 | 34.470 | 25.280 | 8.5 | 0.2 |
| **Ev87** | 2004 | 6 | 10 | 23 | 11 | 9.52 | 34.480 | 25.270 | 7.0 | 0.3 |
| **Ev88** | 2004 | 6 | 11 | 1 | 34 | 43.3 | 34.450 | 25.230 | 10.4 | 0.1 |
| **Ev89** | 2004 | 6 | 11 | 2 | 51 | 9.17 | 34.520 | 25.300 | 9.9 | 0.2 |
| **Ev90** | 2004 | 6 | 11 | 3 | 45 | 20.1 | 34.420 | 25.220 | 2.7 | 0.1 |
| **Ev91** | 2004 | 6 | 11 | 5 | 23 | 27.5 | 34.440 | 25.160 | 7.6 | 0.3 |
| **Ev92** | 2004 | 6 | 11 | 6 | 55 | 36.97 | 34.450 | 25.210 | 10.4 | 0.1 |
| **Ev93** | 2004 | 6 | 11 | 22 | 5 | 15.4 | 34.520 | 25.280 | 9.0 | 0.3 |
| **Ev94** | 2004 | 6 | 11 | 22 | 19 | 10.92 | 34.520 | 25.280 | 8.5 | 0.2 |
| **Ev95** | 2004 | 6 | 11 | 22 | 44 | 10.78 | 34.500 | 25.280 | 13.2 | 0.2 |
| **Ev96** | 2004 | 6 | 11 | 22 | 56 | 7.66 | 34.370 | 25.140 | 9.7 | 0.1 |
| **Ev97** | 2004 | 6 | 12 | 0 | 35 | 16.37 | 34.480 | 25.270 | 8.5 | 0.1 |
| **Ev98** | 2004 | 6 | 12 | 1 | 24 | 41.71 | 34.490 | 25.270 | 4.3 | 0.4 |
| **Ev99** | 2004 | 6 | 12 | 9 | 2 | 50.25 | 34.490 | 25.270 | 3.9 | 0.4 |
| **Ev100** | 2004 | 6 | 12 | 15 | 33 | 51.4 | 34.530 | 25.270 | 4.8 | 0.2 |
| **Ev101** | 2004 | 6 | 12 | 18 | 3 | 50.17 | 34.510 | 25.280 | 10.1 | 0.3 |
| **Ev102** | 2004 | 6 | 12 | 19 | 6 | 39.97 | 34.480 | 25.260 | 3.7 | 0.2 |
| **Ev103** | 2004 | 6 | 12 | 19 | 25 | 56.46 | 34.520 | 25.290 | 9.9 | 0.1 |
| **Ev104** | 2004 | 6 | 12 | 20 | 34 | 36.03 | 34.480 | 25.150 | 3.1 | 0.1 |
| **Ev105** | 2004 | 6 | 12 | 21 | 7 | 26.5 | 34.520 | 25.220 | 8.6 | 0.1 |
| **Ev106** | 2004 | 6 | 12 | 21 | 14 | 41.1 | 34.520 | 25.280 | 8.9 | 0.3 |
| **Ev107** | 2004 | 6 | 12 | 21 | 56 | 11.63 | 34.470 | 25.270 | 8.8 | 0.2 |
| **Ev108** | 2004 | 6 | 13 | 0 | 20 | 54.83 | 34.520 | 25.290 | 14.4 | 0.1 |
| **Ev109** | 2004 | 6 | 13 | 0 | 26 | 34.06 | 34.440 | 25.090 | 21.6 | 0.4 |
| **Ev110** | 2004 | 6 | 13 | 0 | 27 | 17.98 | 34.520 | 25.280 | 9.8 | 0.2 |
| **Ev111** | 2004 | 6 | 13 | 0 | 30 | 6.46 | 34.510 | 25.280 | 8.9 | 0.2 |
| **Ev112** | 2004 | 6 | 13 | 1 | 11 | 57.93 | 34.510 | 25.280 | 8.5 | 0.2 |
| **Ev113** | 2004 | 6 | 13 | 1 | 16 | 24.8 | 34.480 | 25.220 | 12.8 | 0.2 |
| **Ev114** | 2004 | 6 | 13 | 1 | 23 | 23.54 | 34.480 | 25.260 | 4.7 | 0.2 |
| **Ev115** | 2004 | 6 | 13 | 2 | 11 | 53.27 | 34.510 | 25.280 | 8.1 | 0.3 |
| **Ev116** | 2004 | 6 | 13 | 2 | 17 | 53.27 | 34.519 | 25.283 | 8.1 | 0.2 |
| **Ev117** | 2004 | 6 | 13 | 2 | 31 | 43.49 | 34.520 | 25.280 | 9.5 | 0.3 |
| **Ev118** | 2004 | 6 | 13 | 3 | 2 | 23.2 | 34.470 | 25.200 | 3.6 | 0.4 |
| **Ev119** | 2004 | 6 | 13 | 3 | 22 | 20.94 | 34.520 | 25.280 | 9.1 | 0.3 |
| **Ev120** | 2004 | 6 | 13 | 3 | 47 | 46.14 | 34.460 | 25.200 | 13.6 | 0.4 |
| **Ev121** | 2004 | 6 | 13 | 4 | 19 | 41.39 | 34.510 | 25.230 | 4.9 | 0.3 |
| **Ev122** | 2004 | 6 | 13 | 4 | 23 | 8.1 | 34.530 | 25.240 | 4.7 | 0.4 |
| **Ev123** | 2004 | 6 | 13 | 7 | 52 | 30.07 | 34.520 | 25.260 | 3.7 | 0.2 |
| **Ev124** | 2004 | 6 | 13 | 8 | 57 | 34.38 | 34.480 | 25.260 | 4.1 | 0.2 |
| **Ev125** | 2004 | 6 | 13 | 9 | 20 | 57.78 | 34.500 | 25.250 | 2.1 | 0.3 |
| **Ev126** | 2004 | 6 | 13 | 9 | 24 | 24.25 | 34.470 | 25.170 | 2.8 | 0.3 |
| **Ev127** | 2004 | 6 | 13 | 10 | 20 | 22.98 | 34.500 | 25.270 | 3.8 | 0.3 |
| **Ev128** | 2004 | 6 | 13 | 13 | 22 | 58.45 | 34.490 | 25.270 | 6.6 | 0.3 |
| **Ev129** | 2004 | 6 | 14 | 7 | 42 | 44.04 | 34.328 | 25.175 | 8.7 | 1.8 |
| **Ev130** | 2004 | 6 | 14 | 8 | 2 | 11.21 | 34.328 | 25.16.98 | 10.3 | 0.9 |
| **Ev131** | 2004 | 6 | 14 | 8 | 3 | 6.36 | 34.285 | 25.147 | 2.1 | 1.6 |
| **Ev132** | 2004 | 6 | 14 | 22 | 29 | 13.14 | 34.166 | 25.161 | 6.0 | 1.2 |
| **Ev133** | 2004 | 6 | 15 | 1 | 6 | 30.23 | 34.291 | 25.164 | 10.6 | 1.4 |
| **Ev134** | 2004 | 6 | 15 | 5 | 9 | 9.58 | 34.313 | 25.166 | 3.1 | 1.2 |
| **Ev135** | 2004 | 6 | 15 | 6 | 43 | 36.03 | 34.325 | 25.137 | 10.8 | 1.2 |
| **Ev136** | 2004 | 6 | 15 | 21 | 35 | 28.66 | 34.256 | 25.143 | 2.0 | 1.2 |
| **Ev137** | 2004 | 6 | 16 | 0 | 11 | 14.98 | 34.315 | 25.162 | 5.9 | 1.2 |
| **Ev138** | 2004 | 6 | 16 | 0 | 32 | 3.46 | 34.305 | 25.176 | 8.8 | 1.4 |
| **Ev139** | 2004 | 6 | 16 | 0 | 42 | 0.84 | 34.31.14 | 25.167 | 6.1 | 1.0 |
| **Ev140** | 2004 | 6 | 16 | 0 | 45 | 23.31 | 34.288 | 25.164 | 0.4 | 1.4 |
| **Ev141** | 2004 | 6 | 16 | 0 | 46 | 51.58 | 34.314 | 25.164 | 9.1 | 1.5 |
| **Ev142** | 2004 | 6 | 16 | 0 | 53 | 35.44 | 34.255 | 25.165 | 11.3 | 1.8 |
| **Ev143** | 2004 | 6 | 16 | 0 | 55 | 44.8 | 34.312 | 25.160 | 6.3 | 1.0 |
| **Ev144** | 2004 | 6 | 16 | 1 | 2 | 33 | 34.295 | 25.148 | 0.7 | 1.4 |
| **Ev145** | 2004 | 6 | 16 | 2 | 2 | 12.11 | 34.314 | 25.164 | 5.4 | 1.9 |
| **Ev146** | 2004 | 6 | 16 | 2 | 3 | 3.81 | 34.314 | 25.165 | 6.1 | 2.0 |
| **Ev147** | 2004 | 6 | 16 | 2 | 41 | 58.21 | 34.255 | 25.142 | 0.6 | 1.4 |
| **Ev148** | 2004 | 6 | 16 | 2 | 41 | 58.6 | 34.269 | 25.123 | 0.4 | 1.5 |
| **Ev149** | 2004 | 6 | 16 | 4 | 24 | 41.61 | 34.318 | 25.156 | 9.3 | 1.4 |
| **Ev150** | 2004 | 6 | 16 | 4 | 45 | 10.83 | 34.300 | 25.168 | 7.8 | 2.0 |
| **Ev151** | 2004 | 6 | 16 | 7 | 50 | 9.14 | 34.317 | 25.166 | 4.7 | 1.2 |
| **Ev152** | 2004 | 6 | 16 | 11 | 17 | 27.78 | 34.276 | 25.166 | 9.3 | 1.2 |
| **Ev153** | 2004 | 6 | 16 | 13 | 47 | 15.77 | 34.316 | 25.165 | 6.3 | 1.2 |
| **Ev154** | 2004 | 6 | 16 | 15 | 4 | 33.47 | 34.337 | 25.190 | 12.0 | 1.0 |
| **Ev155** | 2004 | 6 | 16 | 18 | 45 | 51.62 | 34.324 | 25.167 | 7.6 | 1.7 |
| **Ev156** | 2004 | 6 | 16 | 19 | 10 | 22.4 | 34.323 | 25.164 | 5.0 | 1.4 |
| **Ev157** | 2004 | 6 | 16 | 19 | 22 | 34.61 | 34.385 | 25.156 | 13.2 | 1.8 |
| **Ev158** | 2004 | 6 | 16 | 19 | 41 | 9.38 | 34.388 | 25.177 | 4.1 | 1.0 |
| **Ev159** | 2004 | 6 | 16 | 23 | 16 | 58.26 | 34.273 | 25.163 | 2.1 | 1.4 |
| **Ev160** | 2004 | 6 | 17 | 1 | 18 | 1.84 | 34.322 | 25.166 | 6.8 | 2.0 |
| **Ev161** | 2004 | 6 | 17 | 7 | 18 | 53.77 | 34.224 | 25.147 | 5.4 | 1.5 |
| **Ev162** | 2004 | 6 | 17 | 10 | 5 | 2.19 | 34.303 | 25.170 | 5.6 | 1.0 |
| **Ev163** | 2004 | 6 | 17 | 13 | 6 | 50.64 | 34.298 | 25.169 | 0.2 | 1.7 |
| **Ev164** | 2004 | 6 | 17 | 13 | 25 | 31.02 | 34.274 | 25.171 | 1.7 | 1.2 |
| **Ev165** | 2004 | 6 | 17 | 14 | 32 | 43.55 | 34.291 | 25.157 | 1.7 | 1.4 |
| **Ev166** | 2004 | 6 | 17 | 19 | 40 | 3.46 | 34.297 | 25.156 | 0.3 | 1.4 |
| **Ev167** | 2004 | 6 | 17 | 21 | 6 | 51.59 | 34.308 | 25.162 | 5.0 | 1.4 |
| **Ev168** | 2004 | 6 | 17 | 21 | 55 | 3.14 | 34.290 | 25.149 | 0.3 | 1.2 |
| **Ev169** | 2004 | 6 | 17 | 23 | 34 | 8.8 | 34.281 | 25.148 | 1.4 | 1.6 |
| **Ev170** | 2004 | 6 | 18 | 0 | 37 | 38.45 | 34.266 | 25.154 | 1.3 | 1.2 |
| **Ev171** | 2004 | 6 | 18 | 2 | 56 | 6.66 | 34.301 | 25.145 | 7.9 | 1.0 |
| **Ev172** | 2004 | 6 | 18 | 7 | 16 | 36.89 | 34.318 | 25.133 | 8.2 | 1.0 |
| **Ev173** | 2004 | 6 | 18 | 7 | 38 | 48.19 | 34.292 | 25.158 | 5.2 | 1.0 |
| **Ev174** | 2004 | 6 | 18 | 8 | 10 | 40.87 | 34.292 | 25.150 | 8.4 | 1.0 |
| **Ev175** | 2004 | 6 | 18 | 8 | 39 | 0.68 | 34.310 | 25.162 | 8.0 | 2.9 |
| **Ev176** | 2004 | 6 | 18 | 9 | 12 | 33.38 | 34.309 | 25.176 | 10.0 | 1.2 |
| **Ev177** | 2004 | 6 | 18 | 9 | 16 | 48.73 | 34.307 | 25.170 | 11.2 | 1.5 |
| **Ev178** | 2004 | 6 | 18 | 10 | 27 | 49.26 | 34.299 | 25.154 | 6.2 | 1.2 |
| **Ev179** | 2004 | 6 | 18 | 10 | 38 | 29.53 | 34.270 | 25.168 | 8.1 | 1.4 |
| **Ev180** | 2004 | 6 | 18 | 10 | 38 | 29.89 | 34.276 | 25.172 | 6.3 | 1.4 |
| **Ev181** | 2004 | 6 | 18 | 13 | 26 | 31.07 | 34.255 | 25.162 | 0.1 | 1.5 |
| **Ev182** | 2004 | 6 | 18 | 14 | 3 | 39.02 | 34.292 | 25.167 | 7.1 | 1.5 |
| **Ev183** | 2004 | 6 | 18 | 16 | 31 | 41.3 | 34.311 | 25.166 | 7.7 | 1.2 |
| **Ev184** | 2004 | 6 | 18 | 18 | 59 | 19.37 | 34.310 | 25.170 | 6.7 | 1.7 |
| **Ev185** | 2004 | 6 | 18 | 20 | 37 | 45.18 | 34.295 | 25.153 | 7.4 | 1.2 |
| **Ev186** | 2004 | 6 | 18 | 22 | 34 | 10.86 | 34.304 | 25.147 | 7.3 | 1.4 |
| **Ev187** | 2004 | 6 | 19 | 1 | 9 | 48.37 | 34.300 | 25.171 | 2.7 | 2.1 |
| **Ev188** | 2004 | 6 | 19 | 6 | 2 | 53.43 | 34.265 | 25.119 | 9.7 | 2.3 |
| **Ev189** | 2004 | 6 | 19 | 11 | 18 | 52.76 | 34.270 | 25.152 | 0.1 | 1.9 |
| **Ev190** | 2004 | 6 | 19 | 11 | 57 | 5.59 | 34.276 | 25.160 | 0.0 | 1.5 |
| **Ev191** | 2004 | 6 | 19 | 12 | 12 | 30.76 | 34.284 | 25.164 | 7.7 | 1.4 |
| **Ev192** | 2004 | 6 | 19 | 12 | 44 | 26.4 | 34.282 | 25.169 | 8.2 | 1.8 |
| **Ev193** | 2004 | 6 | 19 | 13 | 57 | 50.93 | 34.297 | 25.115 | 10.9 | 1.2 |
| **Ev194** | 2004 | 6 | 19 | 14 | 9 | 17.3 | 34.324 | 25.163 | 5.9 | 2.1 |
| **Ev195** | 2004 | 6 | 19 | 14 | 40 | 47.02 | 34.292 | 25.168 | 1.7 | 1.7 |
| **Ev196** | 2004 | 6 | 19 | 14 | 41 | 14.42 | 34.304 | 25.169 | 5.5 | 1.5 |
| **Ev197** | 2004 | 6 | 19 | 14 | 45 | 24.67 | 34.344 | 25.142 | 6.5 | 1.0 |
| **Ev198** | 2004 | 6 | 19 | 14 | 59 | 26.16 | 34.297 | 25.150 | 9.2 | 1.0 |
| **Ev199** | 2004 | 6 | 19 | 15 | 13 | 34.71 | 34.310 | 25.165 | 4.7 | 1.3 |
| **Ev200** | 2004 | 6 | 19 | 23 | 3 | 27.1 | 34.291 | 25.167 | 8.2 | 1.8 |
| **Ev201** | 2004 | 6 | 20 | 9 | 43 | 51.22 | 34.270 | 25.177 | 8.9 | 1.2 |
| **Ev202** | 2004 | 6 | 20 | 18 | 54 | 36.25 | 34.282 | 25.174 | 8.6 | 1.6 |
| **Ev203** | 2004 | 6 | 20 | 19 | 12 | 13.57 | 34.486 | 25.253 | 7.2 | 1.2 |
| **Ev204** | 2004 | 6 | 20 | 19 | 23 | 52.83 | 34.495 | 25.258 | 9.9 | 2.3 |
| **Ev205** | 2004 | 6 | 20 | 20 | 50 | 22.17 | 34.615 | 25.308 | 8.2 | 1.6 |
| **Ev206** | 2004 | 6 | 20 | 23 | 18 | 9.33 | 34.320 | 25.170 | 8.5 | 1.0 |
| **Ev207** | 2004 | 6 | 21 | 23 | 59 | 57.09 | 34.508 | 25.277 | 5.2 | 1.7 |
| **Ev208** | 2004 | 6 | 22 | 0 | 22 | 36.54 | 34.526 | 25.273 | 5.1 | 1.0 |
| **Ev209** | 2004 | 6 | 22 | 4 | 46 | 56.56 | 34.652 | 25.158 | 0.0 | 1.0 |
| **Ev210** | 2004 | 6 | 22 | 6 | 53 | 22.25 | 34.351 | 25.162 | 7.0 | 1.5 |
| **Ev211** | 2004 | 6 | 22 | 7 | 15 | 58.98 | 34.529 | 25.251 | 6.5 | 0.7 |
| **Ev212** | 2004 | 6 | 22 | 7 | 34 | 55.1 | 34.484 | 25.278 | 1.4 | 0.7 |
| **Ev213** | 2004 | 6 | 22 | 7 | 45 | 19.3 | 34.494 | 25.198 | 1.8 | 0.8 |
| **Ev214** | 2004 | 6 | 22 | 7 | 49 | 47.55 | 34.461 | 25.266 | 2.1 | 0.7 |
| **Ev215** | 2004 | 6 | 22 | 8 | 33 | 23.36 | 34.478 | 25.289 | 10.1 | 1.0 |
| **Ev216** | 2004 | 6 | 22 | 8 | 39 | 2.88 | 34.445 | 25.246 | 2.3 | 2.2 |
| **Ev217** | 2004 | 6 | 22 | 11 | 12 | 5.82 | 34.463 | 25.260 | 7.2 | 2.1 |
| **Ev218** | 2004 | 6 | 22 | 14 | 20 | 2.7 | 34.513 | 25.276 | 4.9 | 2.3 |
| **Ev219** | 2004 | 6 | 22 | 16 | 38 | 59.94 | 34.481 | 25.253 | 4.9 | 1.0 |
| **Ev220** | 2004 | 6 | 22 | 17 | 19 | 0.32 | 34.474 | 25.262 | 2.4 | 1.2 |
| **Ev221** | 2004 | 6 | 22 | 19 | 44 | 50 | 34.470 | 25.268 | 1.3 | 0.9 |
| **Ev222** | 2004 | 6 | 23 | 15 | 58 | 6.82 | 34.487 | 25.280 | 8.7 | 0.7 |
| **Ev223** | 2004 | 6 | 23 | 15 | 59 | 40.58 | 34.280 | 25.150 | 0.0 | 1.4 |
| **Ev224** | 2004 | 6 | 23 | 18 | 3 | 35.85 | 34.474 | 25.311 | 1.2 | 0.8 |
| **Ev225** | 2004 | 6 | 23 | 18 | 12 | 36.38 | 34.522 | 25.289 | 9.3 | 1.2 |
| **Ev226** | 2004 | 6 | 23 | 19 | 23 | 12.09 | 34.501 | 25.278 | 9.2 | 1.2 |
| **Ev227** | 2004 | 6 | 23 | 19 | 36 | 51.57 | 34.524 | 25.248 | 3.5 | 1.0 |
| **Ev228** | 2004 | 6 | 23 | 22 | 23 | 11.25 | 34.454 | 25.252 | 2.8 | 0.7 |
| **Ev229** | 2004 | 6 | 24 | 0 | 9 | 25.82 | 34.515 | 25.265 | 5.2 | 0.7 |
| **Ev230** | 2004 | 6 | 24 | 0 | 52 | 41.56 | 34.432 | 25.241 | 0.0 | 1.4 |
| **Ev231** | 2004 | 6 | 24 | 0 | 59 | 42.59 | 34.465 | 25.253 | 2.7 | 1.1 |
| **Ev232** | 2004 | 6 | 24 | 1 | 24 | 30.16 | 34.492 | 25.277 | 9.3 | 0.9 |
| **Ev233** | 2004 | 6 | 24 | 13 | 18 | 0.08 | 34.538 | 25.274 | 11.8 | 1.9 |
| **Ev234** | 2004 | 6 | 24 | 14 | 45 | 46.35 | 34.330 | 25.150 | 1.2 | 1.4 |
| **Ev235** | 2004 | 6 | 24 | 19 | 36 | 25.43 | 34.479 | 25.175 | 14.0 | 1.7 |
| **Ev236** | 2004 | 6 | 24 | 19 | 41 | 12.13 | 34.467 | 25.272 | 10.6 | 0.9 |
| **Ev237** | 2004 | 6 | 24 | 23 | 6 | 15.93 | 34.446 | 25.227 | 7.9 | 1.4 |
| **Ev238** | 2004 | 6 | 26 | 0 | 31 | 42.31 | 34.487 | 25.277 | 8.2 | 1.0 |
| **Ev239** | 2004 | 6 | 26 | 3 | 42 | 46.7 | 34.507 | 25.270 | 6.0 | 1.0 |
| **Ev240** | 2004 | 6 | 26 | 8 | 45 | 54.05 | 34.471 | 25.259 | 9.5 | 1.1 |
| **Ev241** | 2004 | 6 | 26 | 8 | 45 | 55.6 | 34.458 | 25.280 | 2.3 | 0.7 |
| **Ev242** | 2004 | 6 | 26 | 15 | 28 | 6.52 | 34.473 | 25.275 | 5.5 | 1.6 |
| **Ev243** | 2004 | 6 | 26 | 16 | 7 | 52.38 | 34.487 | 25.279 | 3.3 | 1.5 |
| **Ev244** | 2004 | 6 | 26 | 18 | 37 | 12.58 | 34.522 | 25.291 | 9.4 | 1.4 |
| **Ev245** | 2004 | 6 | 26 | 19 | 46 | 2.11 | 34.532 | 25.292 | 9.8 | 1.5 |
| **Ev246** | 2004 | 6 | 26 | 21 | 5 | 33.28 | 34.428 | 25.166 | 14.4 | 1.5 |
| **Ev247** | 2004 | 6 | 26 | 22 | 12 | 34.88 | 34.488 | 25.279 | 8.3 | 1.0 |
| **Ev248** | 2004 | 6 | 26 | 23 | 21 | 43.16 | 34.494 | 25.281 | 7.4 | 0.7 |
| **Ev249** | 2004 | 6 | 26 | 23 | 22 | 12.27 | 34.491 | 25.279 | 6.9 | 2.2 |
| **Ev250** | 2004 | 6 | 27 | 0 | 46 | 30.22 | 34.487 | 25.243 | 2.3 | 1.7 |
| **Ev251** | 2004 | 6 | 27 | 1 | 46 | 43.93 | 34.499 | 25.207 | 1.6 | 0.7 |
| **Ev252** | 2004 | 6 | 27 | 1 | 48 | 33.91 | 34.488 | 25.195 | 0.4 | 0.7 |
| **Ev253** | 2004 | 6 | 27 | 6 | 59 | 43.72 | 34.481 | 25.280 | 8.3 | 1.0 |
| **Ev254** | 2004 | 6 | 27 | 8 | 38 | 25.07 | 34.425 | 25.199 | 12.3 | 1.3 |
| **Ev255** | 2004 | 6 | 27 | 11 | 48 | 47.65 | 34.418 | 25.269 | 0.8 | 2.0 |
| **Ev256** | 2004 | 6 | 27 | 19 | 37 | 55.68 | 34.494 | 25.257 | 3.5 | 1.0 |
| **Ev257** | 2004 | 6 | 27 | 21 | 44 | 33.58 | 34.535 | 25.249 | 8.9 | 1.0 |
| **Ev258** | 2004 | 6 | 27 | 22 | 56 | 45.03 | 34.477 | 25.300 | 5.6 | 1.6 |
| **Ev259** | 2004 | 6 | 28 | 0 | 23 | 31.04 | 34.484 | 25.285 | 8.7 | 1.2 |
| **Ev260** | 2004 | 6 | 28 | 1 | 20 | 14.84 | 34.490 | 25.281 | 8.0 | 1.8 |
| **Ev261** | 2004 | 6 | 28 | 10 | 19 | 6.07 | 34.505 | 25.291 | 11.4 | 1.0 |
| **Ev262** | 2004 | 6 | 28 | 20 | 3 | 25.96 | 34.481 | 25.278 | 8.6 | 1.0 |
| **Ev263** | 2004 | 6 | 28 | 21 | 12 | 12.92 | 34.515 | 25.281 | 7.3 | 1.0 |
| **Ev264** | 2004 | 6 | 28 | 21 | 20 | 35.56 | 34.492 | 25.280 | 8.1 | 1.2 |
| **Ev265** | 2004 | 6 | 28 | 23 | 15 | 42.46 | 34.488 | 25.284 | 8.9 | 1.3 |
| **Ev266** | 2004 | 6 | 29 | 0 | 58 | 50.03 | 34.499 | 25.242 | 9.7 | 2.2 |
| **Ev267** | 2004 | 6 | 29 | 1 | 15 | 17.03 | 34.519 | 25.292 | 9.6 | 2.1 |
| **Ev268** | 2004 | 6 | 29 | 1 | 57 | 30.31 | 34.486 | 25.248 | 1.5 | 1.9 |
| **Ev269** | 2004 | 6 | 29 | 3 | 51 | 54.52 | 34.266 | 25.122 | 0.5 | 1.1 |
| **Ev270** | 2004 | 6 | 29 | 5 | 40 | 57.99 | 34.643 | 25.255 | 8.8 | 1.0 |
| **Ev271** | 2004 | 6 | 29 | 5 | 49 | 4.99 | 34.480 | 25.195 | 11.8 | 1.1 |
| **Ev272** | 2004 | 6 | 29 | 6 | 30 | 40.21 | 34.508 | 25.281 | 9.4 | 1.3 |
| **Ev273** | 2004 | 6 | 29 | 8 | 23 | 22.9 | 34.505 | 25.282 | 9.9 | 1.0 |
| **Ev274** | 2004 | 6 | 29 | 12 | 22 | 25.07 | 34.418 | 25.287 | 10.0 | 1.6 |
| **Ev275** | 2004 | 6 | 29 | 12 | 48 | 19.66 | 34.493 | 25.280 | 7.9 | 1.2 |
| **Ev276** | 2004 | 6 | 29 | 17 | 33 | 07.00 | 34.410 | 25.260 | 1.3 | 1.0 |
| **Ev277** | 2004 | 6 | 29 | 19 | 12 | 28.00 | 34.490 | 25.280 | 6.6 | 1.0 |
| **Ev278** | 2004 | 6 | 29 | 20 | 39 | 34.95 | 34.490 | 25.260 | 9.9 | 0.7 |
| **Ev279** | 2004 | 6 | 29 | 23 | 4 | 52.12 | 34.500 | 25.270 | 3.9 | 1.0 |
| **Ev280** | 2004 | 6 | 29 | 23 | 26 | 10.51 | 34.480 | 25.280 | 5.4 | 0.8 |
| **Ev281** | 2004 | 7 | 21 | 0 | 5 | 59.23 | 34.5 | 25.27 | 7.4 | 1 |
| **Ev282** | 2004 | 7 | 21 | 0 | 7 | 19.47 | 34.48 | 25.25 | 5.4 | 1.3 |
| **Ev283** | 2004 | 7 | 21 | 0 | 16 | 47.64 | 34.55 | 25.26 | 8.4 | 1.3 |
| **Ev284** | 2004 | 7 | 21 | 0 | 29 | 1.43 | 34.51 | 25.21 | 10.0 | 0.7 |
| **Ev285** | 2004 | 7 | 21 | 0 | 31 | 55.18 | 34.47 | 25.27 | 5.6 | 1 |
| **Ev286** | 2004 | 7 | 21 | 0 | 45 | 16.19 | 34.43 | 25.21 | 6.0 | 0.7 |
| **Ev287** | 2004 | 7 | 21 | 0 | 46 | 37.42 | 34.49 | 25.2 | 7.0 | 1 |
| **Ev288** | 2004 | 7 | 21 | 1 | 20 | 39.23 | 34.46 | 25.29 | 3.6 | 0.9 |
| **Ev289** | 2004 | 7 | 21 | 1 | 33 | 31.6 | 34.45 | 25.26 | 4.2 | 0.7 |
| **Ev290** | 2004 | 7 | 21 | 1 | 38 | 48.94 | 34.47 | 25.23 | 4.0 | 0.7 |
| **Ev291** | 2004 | 7 | 21 | 3 | 48 | 26.59 | 34.48 | 25.19 | 6.7 | 1 |
| **Ev292** | 2004 | 7 | 21 | 4 | 16 | 29.83 | 34.53 | 25.26 | 8.6 | 1 |
| **Ev293** | 2004 | 7 | 21 | 4 | 22 | 0.59 | 34.5 | 25.21 | 2.1 | 1 |
| **Ev294** | 2004 | 7 | 21 | 10 | 3 | 29.67 | 34.56 | 25.17 | 0.8 | 1.4 |
| **Ev295** | 2004 | 7 | 21 | 11 | 46 | 56.87 | 34.49 | 25.28 | 7.3 | 1 |
| **Ev296** | 2004 | 7 | 21 | 11 | 51 | 38.29 | 34.4 | 25.19 | 5.1 | 1 |
| **Ev297** | 2004 | 7 | 21 | 11 | 51 | 38.49 | 34.41 | 25.2 | 4.1 | 0.7 |
| **Ev298** | 2004 | 7 | 21 | 14 | 47 | 45.23 | 34.38 | 25.19 | 10.3 | 1 |
| **Ev299** | 2004 | 7 | 21 | 15 | 4 | 13.44 | 34.37 | 25.33 | 7.7 | 1.8 |
| **Ev300** | 2004 | 7 | 21 | 15 | 40 | 31.93 | 34.49 | 25.27 | 4.9 | 1 |
| **Ev301** | 2004 | 7 | 21 | 16 | 1 | 7.27 | 34.52 | 25.27 | 5.0 | 0.5 |
| **Ev302** | 2004 | 7 | 21 | 16 | 17 | 4.52 | 34.6 | 25.17 | 6.9 | 1 |
| **Ev303** | 2004 | 7 | 21 | 18 | 39 | 40.2 | 34.51 | 25.25 | 5.5 | 1 |
| **Ev304** | 2004 | 7 | 21 | 18 | 50 | 40.99 | 34.52 | 25.27 | 5.3 | 0.7 |
| **Ev305** | 2004 | 7 | 21 | 18 | 54 | 34.34 | 34.48 | 25.28 | 5.5 | 0.3 |
| **Ev306** | 2004 | 7 | 21 | 19 | 41 | 34 | 34.4 | 25.23 | 3.9 | 0.7 |
| **Ev307** | 2004 | 7 | 21 | 19 | 42 | 9.39 | 34.44 | 25.28 | 3.7 | 0.7 |
| **Ev308** | 2004 | 7 | 21 | 21 | 4 | 56.42 | 34.46 | 25.29 | 3.2 | 0.7 |
| **Ev309** | 2004 | 7 | 21 | 21 | 24 | 5.01 | 34.5 | 25.26 | 4.8 | 1 |
| **Ev310** | 2004 | 7 | 21 | 21 | 25 | 55.44 | 34.5 | 25.15 | 4.8 | 0.9 |
| **Ev311** | 2004 | 7 | 21 | 21 | 35 | 46.92 | 34.52 | 25.27 | 5.0 | 1 |
| **Ev312** | 2004 | 7 | 21 | 22 | 14 | 59.79 | 34.48 | 25.27 | 2.4 | 1 |
| **Ev313** | 2004 | 7 | 21 | 22 | 15 | 0.54 | 34.49 | 25.27 | 6.4 | 1 |
| **Ev314** | 2004 | 7 | 22 | 0 | 8 | 42.41 | 34.5 | 25.27 | 6.7 | 0.8 |
| **Ev315** | 2004 | 7 | 22 | 2 | 20 | 57.24 | 34.46 | 25.25 | 5.7 | 1 |
| **Ev316** | 2004 | 7 | 22 | 4 | 50 | 21.61 | 34.47 | 25.24 | 11.2 | 1.1 |
| **Ev317** | 2004 | 7 | 22 | 6 | 29 | 28.36 | 34.5 | 25.27 | 4.8 | 0.8 |
| **Ev318** | 2004 | 7 | 22 | 6 | 33 | 48.03 | 34.47 | 25.21 | 3.5 | 0.8 |
| **Ev319** | 2004 | 7 | 22 | 6 | 35 | 26.86 | 34.47 | 25.2 | 11.6 | 0.8 |
| **Ev320** | 2004 | 7 | 22 | 7 | 47 | 46.28 | 34.44 | 25.23 | 1.3 | 1 |
| **Ev321** | 2004 | 7 | 22 | 7 | 48 | 46.83 | 34.48 | 25.21 | 1.3 | 0.7 |
| **Ev322** | 2004 | 7 | 22 | 9 | 6 | 37.33 | 34.51 | 25.25 | 5.8 | 0.7 |
| **Ev323** | 2004 | 7 | 22 | 9 | 9 | 21.63 | 34.51 | 25.27 | 4.5 | 0.3 |
| **Ev324** | 2004 | 7 | 22 | 9 | 10 | 21.76 | 34.52 | 25.27 | 4.1 | 1 |
| **Ev325** | 2004 | 7 | 22 | 9 | 15 | 23.02 | 34.49 | 25.32 | 11.0 | 0.6 |
| **Ev326** | 2004 | 7 | 22 | 11 | 36 | 57.79 | 34.42 | 25.17 | 1.4 | 0.7 |
| **Ev327** | 2004 | 7 | 22 | 11 | 37 | 59.36 | 34.49 | 25.26 | 5.6 | 1 |
| **Ev328** | 2004 | 7 | 22 | 18 | 36 | 23.34 | 34.51 | 25.25 | 7.2 | 0.7 |
| **Ev329** | 2004 | 7 | 22 | 22 | 47 | 56.49 | 34.5 | 25.25 | 7.1 | 1 |
| **Ev330** | 2004 | 7 | 22 | 22 | 53 | 10.85 | 34.5 | 25.27 | 4.0 | 0.7 |
| **Ev331** | 2004 | 7 | 23 | 0 | 10 | 58.72 | 34.49 | 25.27 | 6.3 | 1 |
| **Ev332** | 2004 | 7 | 23 | 1 | 37 | 23.71 | 34.49 | 25.25 | 5.7 | 0.7 |
| **Ev333** | 2004 | 7 | 23 | 1 | 42 | 15.03 | 34.5 | 25.22 | 5.8 | 0.7 |
| **Ev334** | 2004 | 7 | 23 | 3 | 36 | 9.37 | 34.52 | 25.29 | 9.2 | 1 |
| **Ev335** | 2004 | 7 | 23 | 6 | 22 | 7.42 | 34.48 | 25.22 | 2.2 | 1 |
| **Ev336** | 2004 | 7 | 23 | 7 | 4 | 55.49 | 34.51 | 25.28 | 8.4 | 0.7 |
| **Ev337** | 2004 | 7 | 23 | 7 | 41 | 48.8 | 34.49 | 25.22 | 1.3 | 1 |
| **Ev338** | 2004 | 7 | 23 | 9 | 8 | 57.69 | 34.56 | 25.12 | 4.0 | 1.4 |
| **Ev339** | 2004 | 7 | 23 | 14 | 7 | 32.55 | 34.49 | 25.25 | 4.4 | 0.5 |
| **Ev340** | 2004 | 7 | 23 | 17 | 42 | 46.9 | 34.45 | 25.24 | 10.5 | 1 |
| **Ev341** | 2004 | 7 | 23 | 18 | 12 | 43.81 | 34.49 | 25.19 | 7.3 | 1 |
| **Ev342** | 2004 | 7 | 23 | 18 | 13 | 44.55 | 34.5 | 25.2 | 3.6 | 1 |
| **Ev343** | 2004 | 7 | 23 | 20 | 38 | 33.64 | 34.53 | 25.29 | 9.1 | 0.7 |
| **Ev344** | 2004 | 7 | 24 | 0 | 18 | 45.94 | 34.47 | 25.27 | 3.8 | 1 |
| **Ev345** | 2004 | 7 | 24 | 16 | 50 | 20.27 | 34.52 | 25.27 | 5.9 | 0.9 |
| **Ev346** | 2004 | 7 | 24 | 18 | 36 | 14.99 | 34.55 | 25.16 | 3.5 | 1.5 |
| **Ev347** | 2004 | 7 | 24 | 21 | 24 | 21.75 | 34.49 | 25.28 | 8.7 | 1.2 |
| **Ev348** | 2004 | 7 | 24 | 23 | 32 | 13.24 | 34.51 | 25.24 | 4.4 | 0.7 |
| **Ev349** | 2004 | 7 | 25 | 2 | 44 | 20.68 | 34.57 | 25.28 | 5.2 | 1.7 |
| **Ev350** | 2004 | 7 | 25 | 3 | 3 | 35.23 | 34.49 | 25.28 | 7.0 | 0.8 |
| **Ev351** | 2004 | 7 | 25 | 3 | 4 | 51.32 | 34.51 | 25.28 | 7.7 | 0.7 |
| **Ev352** | 2004 | 7 | 25 | 4 | 58 | 33.51 | 34.61 | 25.33 | 10.0 | 0.7 |
| **Ev353** | 2004 | 7 | 25 | 4 | 85 | 33.46 | 34.67 | 25.32 | 9.0 | 1 |
| **Ev354** | 2004 | 7 | 25 | 11 | 16 | 22.11 | 34.5 | 25.26 | 4.7 | 1 |
| **Ev355** | 2004 | 7 | 25 | 16 | 34 | 0.33 | 34.48 | 25.27 | 7.3 | 1 |
| **Ev356** | 2004 | 7 | 25 | 16 | 34 | 48.87 | 34.48 | 25.28 | 6.4 | 0.7 |
| **Ev357** | 2004 | 7 | 25 | 16 | 42 | 22.78 | 34.52 | 25.28 | 4.0 | 0.7 |
| **Ev358** | 2004 | 7 | 25 | 22 | 22 | 3.15 | 34.47 | 25.32 | 4.4 | 0.7 |
| **Ev359** | 2004 | 7 | 25 | 22 | 23 | 42.3 | 34.52 | 25.26 | 10.6 | 1 |
| **Ev360** | 2004 | 7 | 25 | 22 | 50 | 47.07 | 34.48 | 25.21 | 9.6 | 0.7 |
| **Ev361** | 2004 | 7 | 25 | 23 | 16 | 3.2 | 34.53 | 25.28 | 9.0 | 0.7 |
| **Ev362** | 2004 | 7 | 25 | 23 | 19 | 51.95 | 34.53 | 25.28 | 8.9 | 1 |
| **Ev363** | 2004 | 7 | 25 | 23 | 25 | 28.79 | 34.53 | 25.23 | 4.1 | 0.7 |
| **Ev364** | 2004 | 7 | 26 | 2 | 26 | 1.5 | 34.48 | 25.2 | 3.9 | 0.7 |
| **Ev365** | 2004 | 7 | 26 | 9 | 52 | 45.9 | 34.48 | 25.25 | 4.1 | 0.7 |
| **Ev366** | 2004 | 7 | 26 | 11 | 3 | 24.39 | 34.49 | 25.27 | 5.7 | 1 |
| **Ev367** | 2004 | 7 | 26 | 11 | 20 | 12.11 | 34.51 | 25.27 | 5.7 | 1 |
| **Ev368** | 2004 | 7 | 26 | 13 | 6 | 33.09 | 34.42 | 25.27 | 4.4 | 1 |
| **Ev369** | 2004 | 7 | 26 | 16 | 32 | 16.49 | 34.49 | 25.27 | 9.6 | 0.7 |
| **Ev370** | 2004 | 7 | 26 | 16 | 33 | 32.88 | 34.49 | 25.23 | 6.1 | 0.9 |
| **Ev371** | 2004 | 7 | 26 | 17 | 42 | 7.99 | 34.49 | 25.25 | 9.0 | 1 |
| **Ev372** | 2004 | 7 | 26 | 17 | 44 | 4.8 | 34.5 | 25.25 | 7.5 | 0.7 |
| **Ev373** | 2004 | 7 | 26 | 17 | 54 | 22.45 | 34.48 | 25.26 | 3.5 | 0.7 |
| **Ev374** | 2004 | 7 | 26 | 19 | 33 | 25.72 | 34.49 | 25.27 | 6.2 | 0.9 |
| **Ev375** | 2004 | 7 | 26 | 19 | 39 | 24.27 | 34.51 | 25.22 | 4.1 | 1 |
| **Ev376** | 2004 | 7 | 26 | 19 | 43 | 51.18 | 34.52 | 25.28 | 7.8 | 1 |
| **Ev377** | 2004 | 7 | 26 | 19 | 45 | 23.96 | 34.49 | 25.27 | 6.1 | 1 |
| **Ev378** | 2004 | 7 | 26 | 19 | 45 | 24.02 | 34.5 | 25.27 | 5.8 | 0.7 |
| **Ev379** | 2004 | 7 | 26 | 21 | 43 | 53.48 | 34.5 | 25.19 | 4.4 | 0.8 |
| **Ev380** | 2004 | 7 | 27 | 1 | 1 | 34.32 | 34.49 | 25.27 | 5.5 | 0.7 |
| **Ev381** | 2004 | 7 | 27 | 8 | 4 | 2.34 | 34.49 | 25.27 | 4.4 | 0.7 |
| **Ev382** | 2004 | 7 | 27 | 8 | 21 | 5.91 | 34.47 | 25.24 | 3.8 | 0.7 |
| **Ev383** | 2004 | 7 | 27 | 12 | 11 | 37.77 | 34.49 | 25.25 | 5.9 | 0.7 |
| **Ev384** | 2004 | 7 | 27 | 19 | 50 | 5.65 | 34.5 | 25.27 | 4.6 | 0.7 |
| **Ev385** | 2004 | 7 | 27 | 23 | 1 | 3.79 | 34.51 | 25.27 | 3.9 | 0.5 |
| **Ev386** | 2004 | 7 | 28 | 0 | 4 | 36.79 | 34.49 | 25.28 | 7.2 | 0.7 |
| **Ev387** | 2004 | 7 | 28 | 0 | 6 | 14.8 | 34.52 | 25.27 | 6.4 | 1 |
| **Ev388** | 2004 | 7 | 28 | 0 | 9 | 22.48 | 34.52 | 25.29 | 9.4 | 1 |
| **Ev389** | 2004 | 7 | 28 | 2 | 35 | 11.27 | 34.53 | 25.21 | 3.8 | 0.5 |
| **Ev390** | 2004 | 7 | 28 | 2 | 43 | 33.86 | 34.52 | 25.23 | 6.7 | 0.5 |
| **Ev391** | 2004 | 7 | 28 | 4 | 23 | 19.77 | 34.52 | 25.28 | 5.0 | 0.7 |
| **Ev392** | 2004 | 7 | 28 | 7 | 46 | 55.07 | 34.44 | 25.22 | 3.9 | 0.7 |
| **Ev393** | 2004 | 7 | 28 | 8 | 57 | 54.14 | 34.523 | 25.291 | 8.0 | 2.2 |
| **Ev394** | 2004 | 7 | 28 | 13 | 35 | 6.68 | 34.493 | 25.22 | 9.7 | 1.4 |
| **Ev395** | 2004 | 7 | 28 | 15 | 21 | 26.63 | 34.499 | 25.256 | 5.2 | 1.4 |
| **Ev396** | 2004 | 7 | 28 | 21 | 36 | 57.24 | 34.504 | 25.275 | 5.5 | 0.9 |
| **Ev397** | 2004 | 7 | 29 | 1 | 46 | 42 | 34.526 | 25.206 | 13.5 | 1.4 |
| **Ev398** | 2004 | 7 | 29 | 4 | 3 | 35.41 | 34.424 | 25.204 | 0.9 | 1.5 |
| **Ev399** | 2004 | 7 | 29 | 4 | 18 | 19.97 | 34.537 | 25.235 | 6.6 | 1.2 |
| **Ev400** | 2004 | 7 | 29 | 7 | 21 | 9.18 | 34.513 | 25.285 | 9.1 | 1 |
| **Ev401** | 2004 | 7 | 29 | 17 | 12 | 1.66 | 34.655 | 25.066 | 4.9 | 1.4 |
| **Ev402** | 2004 | 7 | 29 | 18 | 4 | 38.27 | 34.523 | 25.276 | 5.5 | 0.7 |
| **Ev403** | 2004 | 7 | 29 | 19 | 33 | 34.18 | 34.526 | 25.21 | 10.1 | 1 |
| **Ev404** | 2004 | 7 | 29 | 19 | 35 | 34.74 | 34.503 | 25.208 | 9.9 | 1 |
| **Ev405** | 2004 | 7 | 29 | 20 | 8 | 31.75 | 34.53 | 25.281 | 6.0 | 0.9 |
| **Ev406** | 2004 | 7 | 29 | 20 | 22 | 49.14 | 34.526 | 25.25 | 8.4 | 0.7 |
| **Ev407** | 2004 | 7 | 29 | 20 | 53 | 16.78 | 34.527 | 25.284 | 7.0 | 0.7 |
| **Ev408** | 2004 | 7 | 29 | 22 | 21 | 1.1 | 34.512 | 25.243 | 7.3 | 0.7 |

Table2: focal mechanism parameters for all earthquakes in Abu Dabbab area

| **Ev no.** | **strike** | **dip** | **rake** | **SD strike** | **SD dip** | **SD rake** | **QP** | **A Gap** |
| --- | --- | --- | --- | --- | --- | --- | --- | --- |
| **EV01** | 143 | 62 | -149 | 0.4 | 0.894427 | 4 | 6 | 109 |
| **EV02** | 344 | 33 | 36 | 0.489901 | 0.894427 | 0.447214 | 1.341641 | 124 |
| **EV03** | 23 | 40 | -61 | 0.4 | 2.408319 | 0 | 3.612479 | 104 |
| **EV04** | 16 | 52 | -51 | 2.79993 | 1.516575 | 4.97996 | 7.46994 | 119 |
| **EV05** | 13 | 49 | -84 | 2.039783 | 3.193744 | 0.894427 | 4.790616 | 190 |
| **EV06** | 352 | 28 | -42 | 0.748338 | 0.894427 | 0.894427 | 1.341641 | 186 |
| **EV07** | 31 | 48 | -29 | 0.4 | 0 | 2.302173 | 3.45326 | 89 |
| **EV08** | 63 | 75 | -133 | 2.79993 | 1.788854 | 1.30384 | 4.199895 | 116 |
| **EV09** | 331 | 49 | 61 | 1.999974 | 0.894427 | 3.962323 | 5.943485 | 90 |
| **EV10** | 22 | 46 | -61 | 0.4 | 0.547723 | 1.095445 | 1.643168 | 91 |
| **EV11** | 7 | 64 | -44 | 2.79993 | 0.894427 | 1.67332 | 4.199895 | 85 |
| **EV12** | 170 | 50 | -52 | 1.599987 | 0 | 1.923538 | 2.885307 | 135 |
| **EV13** | 153 | 35 | -110 | 2.399956 | 2.607681 | 4.219005 | 6.328508 | 114 |
| **EV14** | 122 | 43 | -13 | 1.599987 | 0.447214 | 2.302173 | 3.45326 | 95 |
| **EV15** | 36 | 73 | -60 | 1.999974 | 0.447214 | 2.302173 | 3.45326 | 88 |
| **EV16** | 151 | 49 | -119 | 2.79993 | 1.788854 | 2.701851 | 4.199895 | 163 |
| **EV17** | 212 | 72 | -43 | 2.79993 | 1.516575 | 4.97996 | 7.46994 | 214 |
| **EV18** | 164 | 45 | 136 | 3.199895 | 1.341641 | 1.581139 | 4.799843 | 106 |
| **EV19** | 81 | 36 | -98 | 3.322694 | 0.83666 | 1.224745 | 4.984041 | 155 |
| **EV20** | 177 | 48 | -134 | 2.79993 | 1.516575 | 4.97996 | 7.46994 | 134 |
| **EV21** | 356 | 55 | -115 | 1.999974 | 3.714835 | 4.147288 | 6.220932 | 118 |
| **EV22** | 158 | 45 | 140 | 2.399956 | 2.683282 | 2.701851 | 4.052777 | 102 |
| **EV23** | 210 | 59 | -47 | 2.79993 | 1.788854 | 2.701851 | 4.199895 | 162 |
| **EV24** | 5 | 64 | 143 | 2.79993 | 1.516575 | 4.97996 | 7.46994 | 102 |
| **EV25** | 156 | 27 | -46 | 2.399956 | 3.130495 | 1.30384 | 4.695743 | 106 |
| **EV26** | 243 | 38 | -48 | 0 | 0.447214 | 1.30384 | 1.95576 | 82 |
| **EV27** | 7 | 40 | -106 | 0.4 | 1.788854 | 1.30384 | 2.683281 | 123 |
| **EV28** | 222 | 63 | -31 | 2.399956 | 0 | 2.701851 | 4.052777 | 106 |
| **EV29** | 138 | 34 | -138 | 0.489901 | 2.828427 | 0 | 4.242641 | 91 |
| **EV30** | 139 | 47 | -169 | 8.54E-07 | 1.224745 | 0.547723 | 1.837118 | 105 |
| **EV31** | 143 | 62 | -155 | 0.4 | 0 | 1.30384 | 1.95576 | 113 |
| **EV32** | 346 | 36 | -126 | 0.979818 | 0.547723 | 0.707107 | 1.469727 | 109 |
| **EV33** | 8 | 44 | -24 | 0.799998 | 0.447214 | 3.535534 | 5.303301 | 106 |
| **EV34** | 320 | 48 | -155 | 0 | 0.547723 | 2.915476 | 4.373214 | 147 |
| **EV35** | 315 | 45 | -32 | 0.799998 | 0 | 3.535534 | 5.303301 | 204 |
| **EV36** | 17 | 52 | -152 | 2.399956 | 3.130495 | 2.302173 | 4.695743 | 150 |
| **EV37** | 349 | 43 | 114 | 3.919028 | 0.447214 | 0.447214 | 5.878542 | 94 |
| **EV38** | 37 | 65 | -25 | 0.4 | 0.547723 | 4.301163 | 6.451745 | 134 |
| **EV39** | 232 | 57 | -151 | 2.399956 | 2.683282 | 1.923538 | 4.024923 | 88 |
| **EV40** | 172 | 50 | -80 | 0.4 | 2.236068 | 1.581139 | 3.354102 | 130 |
| **EV41** | 29 | 36 | -139 | 1.999974 | 2.683282 | 2.701851 | 4.052777 | 97 |
| **EV42** | 199 | 43 | -22 | 2.79993 | 1.30384 | 4.219005 | 6.328508 | 160 |
| **EV43** | 316 | 48 | -144 | 0.489901 | 1.732051 | 0.547723 | 2.598077 | 113 |
| **EV44** | 18 | 64 | 127 | 0.4 | 0.447214 | 1.140175 | 1.710263 | 153 |
| **EV45** | 31 | 48 | -50 | 0.748338 | 0 | 0.894427 | 1.341641 | 112 |
| **EV46** | 195 | 74 | -52 | 1.600048 | 3.03315 | 0.83666 | 4.549725 | 100 |
| **EV47** | 114 | 60 | -166 | 0.894443 | 0 | 0.707107 | 1.341665 | 109 |
| **EV48** | 225 | 74 | 157 | 0.799998 | 0.83666 | 4.868265 | 7.302398 | 92 |
| **EV49** | 11 | 43 | -50 | 0 | 0.447214 | 1.923538 | 2.885307 | 105 |
| **EV50** | 213 | 68 | 137 | 2.399956 | 3.834058 | 4.207137 | 6.310706 | 139 |
| **EV51** | 298 | 42 | 44 | 0.489901 | 1.095445 | 0.83666 | 1.643168 | 82 |
| **EV52** | 133 | 45 | -153 | 3.999793 | 0 | 3.535534 | 5.99969 | 67 |
| **EV53** | 340 | 55 | 72 | 1.356522 | 0.547723 | 0.707107 | 2.034783 | 58 |
| **EV54** | 191 | 35 | 115 | 2.79993 | 1.341641 | 3.114482 | 4.671723 | 57 |
| **EV55** | 32 | 55 | -33 | 1.199995 | 0.447214 | 1.140175 | 1.799993 | 60 |
| **EV56** | 139 | 53 | -119 | 2.154291 | 1.643168 | 3.209361 | 4.814042 | 71 |
| **EV57** | 126 | 58 | -152 | 1.999974 | 0.894427 | 1.923538 | 2.999961 | 56 |
| **EV58** | 33 | 47 | -23 | 0.799998 | 2.966479 | 0.447214 | 4.449719 | 64 |
| **EV59** | 61 | 57 | -16 | 0.4 | 0.447214 | 1.30384 | 1.95576 | 59 |
| **EV60** | 36 | 44 | -60 | 0.799998 | 2.236068 | 1.30384 | 3.354102 | 98 |
| **EV61** | 125 | 50 | -124 | 0.489901 | 0.547723 | 0.547723 | 0.821585 | 98 |
| **EV62** | 150 | 53 | 112 | 0.4 | 3.130495 | 1.581139 | 4.695743 | 103 |
| **EV63** | 25 | 41 | -57 | 0 | 0.894427 | 1.30384 | 1.95576 | 66 |
| **EV64** | 29 | 59 | -64 | 2.561374 | 0.83666 | 0.83666 | 3.842061 | 105 |
| **EV65** | 166 | 72 | 60 | 0.748338 | 0.547723 | 1.30384 | 1.95576 | 130 |
| **EV66** | 135 | 30 | -141 | 3.59985 | 0 | 3.563706 | 5.399775 | 70 |
| **EV67** | 179 | 60 | 145 | 0.799998 | 1.341641 | 2.701851 | 4.052777 | 87 |
| **EV68** | 218 | 45 | -74 | 0.4 | 0.547723 | 1.30384 | 1.95576 | 143 |
| **EV69** | 12 | 48 | -89 | 3.577677 | 0 | 3.04959 | 5.366516 | 67 |
| **EV70** | 208 | 33 | -23 | 2.79993 | 1.516575 | 4.97996 | 7.46994 | 84 |
| **EV71** | 58 | 61 | 61 | 1.199995 | 1.140175 | 0.547723 | 1.799993 | 81 |
| **EV72** | 62 | 42 | 58 | 1.095453 | 4.037326 | 0.547723 | 6.055989 | 66 |
| **EV73** | 333 | 45 | 75 | 0.748338 | 0.547723 | 0.447214 | 1.122507 | 98 |
| **EV74** | 39 | 57 | 36 | 0 | 0 | 3.535534 | 5.303301 | 87 |
| **EV75** | 192 | 53 | -28 | 1.199995 | 4.472136 | 3.535534 | 6.708204 | 67 |
| **EV76** | 243 | 57 | -11 | 2.399956 | 2.236068 | 3.962323 | 5.943485 | 74 |
| **EV77** | 216 | 50 | -76 | 2.79993 | 1.516575 | 4.97996 | 7.46994 | 77 |
| **EV78** | 272 | 68 | -132 | 0.800006 | 1.81659 | 0.447214 | 2.724885 | 111 |
| **EV79** | 174 | 51 | -129 | 1.199995 | 0.447214 | 1.581139 | 2.371709 | 193 |
| **EV80** | 360 | 61 | -36 | 0 | 0 | 1.581139 | 2.371709 | 69 |
| **EV81** | 157 | 66 | 26 | 1.999974 | 0.447214 | 2.774887 | 4.162331 | 191 |
| **EV82** | 43 | 51 | -156 | 2.399956 | 0.447214 | 4.97996 | 7.46994 | 125 |
| **EV83** | 328 | 28 | -125 | 0.799998 | 0.894427 | 4.037326 | 6.055989 | 74 |
| **EV84** | 146 | 51 | -126 | 0.4 | 0 | 1.581139 | 2.371709 | 81 |
| **EV85** | 172 | 61 | 133 | 0.748338 | 0.447214 | 0.447214 | 1.122507 | 64 |
| **EV86** | 98 | 54 | 60 | 0.799998 | 0.447214 | 1.923538 | 2.885307 | 95 |
| **EV87** | 145 | 22 | 34 | 1.999974 | 2.387476 | 4.147288 | 6.220932 | 79 |
| **EV88** | 355 | 62 | -51 | 3.199895 | 0.447214 | 1.30384 | 4.799843 | 103 |
| **EV89** | 159 | 48 | -130 | 1.999974 | 2.607681 | 1 | 3.911522 | 65 |
| **EV90** | 329 | 41 | -107 | 1.496712 | 3.271085 | 0.894427 | 4.906628 | 146 |
| **EV91** | 44 | 66 | -61 | 1.599987 | 2.236068 | 1.923538 | 3.354102 | 181 |
| **EV92** | 40 | 86 | -43 | 0.4 | 3.577709 | 5.049752 | 7.574628 | 91 |
| **EV93** | 2 | 72 | -23 | 0.489901 | 2.167948 | 0.447214 | 3.251922 | 75 |
| **EV94** | 26 | 52 | -34 | 2.399956 | 2.683282 | 1.581139 | 4.024923 | 80 |
| **EV95** | 31 | 67 | -26 | 0.348799 | 2.738613 | 4.1833 | 6.27495 | 53 |
| **EV96** | 155 | 39 | 33 | 0.800006 | 4.266146 | 0.547723 | 6.399219 | 145 |
| **EV97** | 310 | 68 | -114 | 0.4 | 0.447214 | 1.923538 | 2.885307 | 85 |
| **EV98** | 348 | 46 | -29 | 2.79993 | 1.516575 | 4.97996 | 7.46994 | 61 |
| **EV99** | 350 | 67 | -33 | 2.79993 | 1.516575 | 4.979996 | 7.46994 | 61 |
| **EV100** | 241 | 70 | -51 | 1.999974 | 3.577709 | 1.30384 | 5.366564 | 75 |
| **EV101** | 86 | 79 | -149 | 1.356522 | 0.547723 | 1.643168 | 2.464752 | 79 |
| **EV102** | 34 | 42 | -32 | 0 | 0 | 3.114482 | 4.671723 | 91 |
| **EV103** | 76 | 68 | -21 | 0 | 0.447214 | 1.30384 | 1.95576 | 118 |
| **EV104** | 200 | 54 | -51 | 0.799998 | 0.547723 | 5.049752 | 7.574628 | 106 |
| **EV105** | 30 | 42 | -41 | 0.799998 | 0.547723 | 0.83666 | 1.25499 | 98 |
| **EV106** | 22 | 51 | -34 | 2.399956 | 1.788854 | 1.30384 | 3.599934 | 64 |
| **EV107** | 174 | 80 | -43 | 1.854726 | 3.361547 | 0.894427 | 5.042321 | 63 |
| **EV108** | 356 | 61 | 57 | 3.199895 | 0.447214 | 1.923538 | 4.799843 | 72 |
| **EV109** | 152 | 49 | 24 | 2.79993 | 0.447214 | 3.535534 | 5.303301 | 152 |
| **EV110** | 142 | 66 | -142 | 0 | 1.341641 | 2.701851 | 4.052777 | 60 |
| **EV111** | 257 | 20 | -115 | 2.79993 | 0.83666 | 5.049752 | 7.574628 | 80 |
| **EV112** | 160 | 57 | 71 | 1.67335 | 0 | 0.894427 | 2.510025 | 81 |
| **EV113** | 11 | 63 | 39 | 2.856915 | 1.414214 | 0.894427 | 4.285373 | 122 |
| **EV114** | 7 | 28 | -149 | 2.79993 | 1.516575 | 4.97996 | 7.46994 | 112 |
| **EV115** | 5 | 44 | -135 | 2.79993 | 2.50998 | 4.505552 | 6.758328 | 117 |
| **EV116** | 160 | 53 | 59 | 0.4 | 0 | 2.701851 | 4.052777 | 82 |
| **EV117** | 149 | 69 | -134 | 0.799998 | 0.894427 | 4.037326 | 6.055989 | 82 |
| **EV118** | 36 | 48 | -152 | 0.4 | 0.894427 | 3.535534 | 5.303301 | 80 |
| **EV119** | 12 | 45 | 67 | 0.799998 | 1.095445 | 3.974921 | 5.962382 | 79 |
| **EV120** | 25 | 73 | 164 | 2.399956 | 1.30384 | 4.219005 | 6.328508 | 141 |
| **EV121** | 191 | 44 | 166 | 0.4 | 1.723051 | 1.788854 | 2.683281 | 84 |
| **EV122** | 59 | 43 | -57 | 1.999974 | 3.130495 | 3.535534 | 5.303301 | 105 |
| **EV123** | 132 | 37 | -157 | 0.799998 | 0 | 1.923538 | 2.885307 | 99 |
| **EV124** | 315 | 64 | -139 | 2.79993 | 1.516575 | 4.97996 | 7.46994 | 71 |
| **EV125** | 210 | 65 | 157 | 2.79993 | 1.516575 | 4.97996 | 7.46994 | 80 |
| **EV126** | 151 | 51 | -131 | 0.799998 | 1.30384 | 3.646917 | 5.470376 | 120 |
| **EV127** | 147 | 41 | -106 | 1.095453 | 0.83666 | 0.447214 | 1.64318 | 91 |
| **EV128** | 322 | 53 | -68 | 1.199995 | 2.683282 | 1.923538 | 4.024923 | 102 |
| **EV129** | 301 | 58 | 124 | 0.489901 | 0.447214 | 1 | 1.5 | 82 |
| **EV130** | 27 | 35 | 84 | 0.799998 | 0.894427 | 1.923538 | 2.885307 | 79 |
| **EV131** | 266 | 50 | -111 | 2.79993 | 1.516575 | 4.97996 | 7.46994 | 86 |
| **EV132** | 166 | 40 | 116 | 2.79993 | 2.236068 | 1.923538 | 4.199895 | 85 |
| **EV133** | 142 | 71 | -152 | 2.399956 | 4.472136 | 3.114482 | 6.708204 | 93 |
| **EV134** | 51 | 38 | -71 | 2.79993 | 1.341641 | 3.962323 | 5.943485 | 82 |
| **EV135** | 92 | 46 | -64 | 0.800006 | 1.140175 | 2.345208 | 3.517812 | 116 |
| **EV136** | 285 | 45 | -113 | 3.999793 | 0 | 3.535534 | 5.99969 | 94 |
| **EV137** | 314 | 17 | 124 | 0.632457 | 0.894427 | 3.209361 | 4.814042 | 61 |
| **EV138** | 188 | 77 | 28 | 1.095453 | 2.792848 | 0.447214 | 4.189272 | 68 |
| **EV139** | 45 | 54 | -24 | 2.79993 | 1.516575 | 4.97996 | 7.46994 | 58 |
| **EV140** | 6 | 69 | -28 | 1.600021 | 0 | 0.894427 | 2.400032 | 64 |
| **EV141** | 36 | 78 | -130 | 1.019818 | 2.607681 | 1 | 3.911522 | 60 |
| **EV142** | 249 | 39 | 138 | 2.79993 | 0.447214 | 1.30384 | 4.199895 | 127 |
| **EV143** | 353 | 46 | -25 | 8.54E-07 | 0 | 3.962323 | 5.943485 | 57 |
| **EV144** | 299 | 64 | -155 | 0.748338 | 0.707107 | 0 | 1.122507 | 83 |
| **EV145** | 337 | 52 | -39 | 0.489901 | 2.19089 | 2.073644 | 3.286335 | 60 |
| **EV146** | 112 | 30 | 123 | 3.999793 | 0.547723 | 2.880972 | 5.99969 | 59 |
| **EV147** | 311 | 75 | 18 | 2.332364 | 1.341641 | 1 | 3.498546 | 92 |
| **EV148** | 350 | 72 | 7 | 2.79993 | 0.894427 | 1.581139 | 4.199895 | 95 |
| **EV149** | 1 | 70 | -52 | 1.199995 | 1.643168 | 5.049752 | 7.574628 | 82 |
| **EV150** | 353 | 71 | -38 | 2.399956 | 4.472136 | 1.30384 | 6.708204 | 65 |
| **EV151** | 174 | 36 | 147 | 2.399956 | 4.159327 | 4.97996 | 7.46994 | 98 |
| **EV152** | 64 | 68 | 146 | 2.79993 | 1.516575 | 4.97996 | 7.46994 | 128 |
| **EV153** | 143 | 69 | -138 | 2.399956 | 4.472136 | 2.701851 | 6.708204 | 90 |
| **EV154** | 9 | 71 | 8 | 3.999793 | 0 | 1.30384 | 5.99969 | 144 |
| **EV155** | 347 | 44 | -24 | 2.79993 | 1.516575 | 4.97996 | 7.46994 | 73 |
| **EV156** | 164 | 46 | -158 | 2.79993 | 1.516575 | 4.97996 | 7.46994 | 125 |
| **EV157** | 107 | 88 | 31 | 3.199895 | 2.683282 | 1.30384 | 4.799843 | 127 |
| **EV158** | 254 | 37 | 61 | 2.79993 | 1.341641 | 1.581139 | 4.199895 | 94 |
| **EV159** | 264 | 65 | -82 | 0.4 | 0 | 1.30384 | 1.95576 | 90 |
| **EV160** | 350 | 39 | 83 | 3.655361 | 3.420526 | 3.937004 | 5.905506 | 69 |
| **EV161** | 167 | 25 | -8 | 1.599987 | 0.894427 | 1.581139 | 2.399981 | 121 |
| **EV162** | 42 | 58 | -36 | 1.199995 | 2.167948 | 4.219005 | 6.328508 | 60 |
| **EV163** | 233 | 69 | -19 | 0 | 0.447214 | 3.962323 | 5.943484 | 96 |
| **EV164** | 336 | 48 | -60 | 0 | 0.447214 | 3.962323 | 5.943485 | 96 |
| **EV165** | 176 | 77 | -43 | 0.4 | 3.271085 | 2.828427 | 4.906628 | 73 |
| **EV166** | 307 | 69 | -141 | 8.54E-07 | 0.547723 | 2.915476 | 4.373214 | 93 |
| **EV167** | 316 | 58 | 145 | 2.79993 | 1.516575 | 4.97996 | 7.46994 | 72 |
| **EV168** | 11 | 74 | 174 | 0.894443 | 0 | 0.83666 | 1.341665 | 74 |
| **EV169** | 350 | 84 | 5 | 2.79993 | 1.949359 | 4.97996 | 7.46994 | 86 |
| **EV170** | 314 | 55 | -146 | 1.999974 | 2.683282 | 1.30384 | 4.024923 | 113 |
| **EV171** | 193 | 31 | -76 | 0.4 | 0 | 1.140175 | 1.710263 | 62 |
| **EV172** | 131 | 58 | -131 | 8.54E-07 | 0 | 3.768289 | 5.652434 | 93 |
| **EV173** | 292 | 51 | 67 | 1.999974 | 3.130495 | 3.962323 | 5.943485 | 66 |
| **EV174** | 335 | 58 | -92 | 8.54E-07 | 0 | 2.302173 | 3.45326 | 120 |
| **EV175** | 142 | 74 | -121 | 2.79993 | 1.341641 | 1.30384 | 4.199895 | 74 |
| **EV176** | 289 | 67 | 7 | 0 | 0 | 1.581139 | 2.371709 | 60 |
| **EV177** | 61 | 53 | -30 | 1.599987 | 0 | 3.535534 | 5.303301 | 62 |
| **EV178** | 142 | 45 | -139 | 2.24516 | 4.266146 | 5.310367 | 7.965551 | 71 |
| **EV179** | 22 | 82 | -13 | 8.54E-07 | 0.447214 | 3.768289 | 5.652434 | 79 |
| **EV180** | 118 | 39 | -147 | 0.4 | 0.547723 | 3.420526 | 5.130789 | 81 |
| **EV181** | 308 | 81 | 179 | 1.743557 | 1.516575 | 1.30384 | 2.615336 | 67 |
| **EV182** | 350 | 42 | -52 | 2.79993 | 0.894427 | 2.302173 | 4.199895 | 97 |
| **EV183** | 285 | 56 | 35 | 1.264948 | 2.302173 | 0.83666 | 3.45326 | 104 |
| **EV184** | 53 | 63 | -32 | 2.79993 | 1.516575 | 4.97996 | 7.46994 | 81 |
| **EV185** | 89 | 48 | -47 | 2.399956 | 1.095445 | 4.722288 | 7.083431 | 77 |
| **EV186** | 357 | 53 | -50 | 2.227169 | 3.130495 | 0.894427 | 4.695743 | 89 |
| **EV187** | 312 | 42 | -31 | 1.199995 | 2.683282 | 2.701851 | 4.052777 | 147 |
| **EV188** | 202 | 42 | -133 | 1.166197 | 1.140175 | 0.447214 | 1.749296 | 100 |
| **EV189** | 357 | 75 | -32 | 1.166218 | 1.095445 | 0 | 1.749327 | 107 |
| **EV190** | 320 | 67 | -146 | 1.41426 | 0.447214 | 0 | 2.12139 | 84 |
| **EV191** | 284 | 61 | -144 | 3.199895 | 2.828427 | 4.97996 | 7.46994 | 85 |
| **EV192** | 41 | 49 | 35 | 1.599987 | 2.236068 | 3.962323 | 5.943485 | 71 |
| **EV193** | 115 | 40 | -157 | 0.4 | 0.894427 | 1.140175 | 1.710263 | 121 |
| **EV194** | 64 | 49 | -60 | 0.799998 | 0.447214 | 1.140175 | 1.710263 | 132 |
| **EV195** | 332 | 50 | -123 | 0.4 | 1.341641 | 1.30384 | 2.012462 | 122 |
| **EV196** | 135 | 60 | -107 | 2.800505 | 4.219005 | 0.547723 | 6.328508 | 95 |
| **EV197** | 326 | 38 | 137 | 0.489901 | 1.81659 | 0.547723 | 2.724885 | 77 |
| **EV198** | 139 | 55 | -116 | 0.979799 | 0.894427 | 3.130495 | 4.695743 | 77 |
| **EV199** | 356 | 30 | -38 | 0.799998 | 1.788854 | 1.923538 | 2.885307 | 103 |
| **EV200** | 338 | 52 | -54 | 2.79993 | 1.516575 | 4.97996 | 7.46994 | 63 |
| **EV201** | 87 | 55 | 113 | 2.79993 | 0.447214 | 3.962323 | 5.943485 | 74 |
| **EV202** | 158 | 37 | 109 | 0.799998 | 3.834058 | 2.302173 | 5.751087 | 93 |
| **EV203** | 323 | 59 | 58 | 0.799998 | 0 | 3.535534 | 5.303301 | 124 |
| **EV204** | 68 | 47 | 137 | 1.199995 | 0.447214 | 1.30384 | 1.95576 | 107 |
| **EV205** | 320 | 44 | -122 | 2.244986 | 5.477226 | 5.049752 | 8.215839 | 99 |
| **EV206** | 129 | 64 | 106 | 3.577677 | 1.341641 | 0.894427 | 5.366516 | 68 |
| **EV207** | 137 | 61 | 101 | 2.871044 | 0.83666 | 1.140175 | 4.306566 | 90 |
| **EV208** | 143 | 53 | 125 | 2.79993 | 1.732051 | 4.219005 | 6.328508 | 62 |
| **EV209** | 255 | 74 | 15 | 2.79993 | 1.516575 | 4.97996 | 7.46994 | 105 |
| **EV210** | 247 | 70 | -44 | 0.489901 | 0.547723 | 0.447214 | 0.821585 | 85 |
| **EV211** | 96 | 42 | 145 | 0.799998 | 0.707107 | 0.894427 | 1.341641 | 81 |
| **EV212** | 23 | 54 | -15 | 1.199995 | 0 | 3.962323 | 5.943485 | 78 |
| **EV213** | 25 | 62 | 117 | 0.4 | 0.894427 | 1.30384 | 1.95576 | 74 |
| **EV214** | 144 | 57 | 36 | 0 | 0.894427 | 1.140175 | 1.710263 | 63 |
| **EV215** | 239 | 67 | 160 | 0.4 | 0.447214 | 1.788854 | 2.683281 | 88 |
| **EV216** | 268 | 43 | -41 | 1.549197 | 2.280351 | 3.847077 | 5.770616 | 78 |
| **EV217** | 351 | 27 | 75 | 2.939979 | 0.447214 | 0.894427 | 4.409969 | 59 |
| **EV218** | 360 | 49 | -34 | 2.79993 | 1.224745 | 5.049752 | 7.574628 | 67 |
| **EV219** | 4 | 44 | 70 | 0 | 0.447214 | 1.30384 | 1.95576 | 89 |
| **EV220** | 255 | 62 | -29 | 0 | 0.447214 | 1.30384 | 1.95576 | 89 |
| **EV221** | 316 | 45 | -126 | 0.748338 | 0 | 0.547723 | 1.122507 | 74 |
| **EV222** | 348 | 69 | -132 | 2.449832 | 2.683282 | 0.83666 | 4.024923 | 91 |
| **EV223** | 281 | 69 | 173 | 2.154291 | 3.391165 | 0.547723 | 5.086748 | 105 |
| **EV224** | 326 | 52 | -127 | 4.309932 | 0.447214 | 1.30384 | 6.464898 | 110 |
| **EV225** | 166 | 47 | -137 | 2.79993 | 1.341641 | 2.302173 | 4.199895 | 76 |
| **EV226** | 37 | 38 | 66 | 1.166197 | 0.447214 | 0.547723 | 1.749296 | 89 |
| **EV227** | 290 | 66 | 27 | 0.799998 | 0.894427 | 3.535534 | 5.303301 | 106 |
| **EV228** | 314 | 34 | -113 | 0.799998 | 0.447214 | 1.923538 | 2.885307 | 77 |
| **EV229** | 214 | 37 | 25 | 0.799998 | 0.894427 | 2.302173 | 3.45326 | 54 |
| **EV230** | 2 | 61 | -138 | 1.999974 | 0 | 1.923538 | 2.999961 | 71 |
| **EV231** | 334 | 53 | -121 | 0.4 | 1.095445 | 1.30384 | 1.95576 | 55 |
| **EV232** | 135 | 38 | 110 | 0.979799 | 0.547723 | 0.447214 | 1.469699 | 58 |
| **EV233** | 315 | 43 | 36 | 0 | 3.962323 | 0.447214 | 5.943485 | 70 |
| **EV234** | 224 | 54 | -123 | 2.939979 | 0.547723 | 1.581139 | 4.409969 | 63 |
| **EV235** | 100 | 45 | -164 | 3.59985 | 0.447214 | 2.701851 | 5.399775 | 108 |
| **EV236** | 309 | 54 | -109 | 0.799998 | 0.447214 | 4.086563 | 6.129845 | 83 |
| **EV237** | 347 | 61 | -139 | 1.549197 | 0.547723 | 0.547723 | 2.323796 | 101 |
| **EV238** | 82 | 43 | 130 | 0.979818 | 1.095445 | 0.83666 | 1.643168 | 98 |
| **EV239** | 319 | 25 | 97 | 2.79993 | 1.341641 | 3.962323 | 5.943485 | 68 |
| **EV240** | 176 | 55 | 173 | 0.4 | 0 | 1.140175 | 1.710263 | 79 |
| **EV241** | 212 | 45 | 31 | 2.79993 | 1.516575 | 4.97996 | 7.46994 | 76 |
| **EV242** | 354 | 58 | 129 | 2.79993 | 1.516575 | 4.97996 | 7.46994 | 67 |
| **EV243** | 102 | 53 | -67 | 1.599987 | 1.341641 | 4.147288 | 6.220932 | 71 |
| **EV244** | 72 | 52 | 103 | 0.4 | 0.894427 | 4.037326 | 6.055989 | 74 |
| **EV245** | 158 | 74 | -161 | 0 | 0.547723 | 0.83666 | 1.25499 | 67 |
| **EV246** | 91 | 38 | -130 | 3.033099 | 4.086563 | 5.049752 | 7.574628 | 95 |
| **EV247** | 87 | 44 | 129 | 1.199995 | 0 | 2.302173 | 3.45326 | 61 |
| **EV248** | 83 | 65 | -22 | 0.799998 | 3.391165 | 4.527693 | 6.79154 | 91 |
| **EV249** | 355 | 39 | -34 | 0.4 | 0.447214 | 1.67332 | 2.50998 | 79 |
| **EV250** | 23 | 65 | -102 | 0.748338 | 1.224745 | 1.30384 | 1.95576 | 73 |
| **EV251** | 289 | 51 | -134 | 0.489901 | 0.547723 | 0.547723 | 0.734852 | 77 |
| **EV252** | 334 | 52 | 36 | 1.999974 | 4.38178 | 3.646917 | 5.470376 | 78 |
| **EV253** | 325 | 46 | 62 | 2.75712 | 1.949359 | 2.302173 | 4.13568 | 112 |
| **EV254** | 66 | 33 | 118 | 1.264948 | 1.643168 | 0.83666 | 2.464752 | 122 |
| **EV255** | 23 | 56 | 145 | 2.399956 | 4.159327 | 2.915476 | 6.238991 | 85 |
| **EV256** | 270 | 49 | 47 | 0.799998 | 0.447214 | 1.923538 | 2.885307 | 74 |
| **EV257** | 163 | 62 | 143 | 0.489901 | 0 | 1.140175 | 1.710263 | 92 |
| **EV258** | 316 | 31 | 45 | 0.4 | 1 | 0.547723 | 1.5 | 81 |
| **EV259** | 175 | 87 | 171 | 0 | 2.236068 | 1.581139 | 3.354102 | 111 |
| **EV260** | 313 | 40 | 115 | 2.79993 | 1.516575 | 4.97996 | 7.46994 | 111 |
| **EV261** | 175 | 43 | 38 | 0.632457 | 1.095445 | 4.97996 | 7.46994 | 97 |
| **EV262** | 137 | 63 | 132 | 0.4 | 0 | 1.923538 | 2.885307 | 117 |
| **EV263** | 13 | 83 | -20 | 2.79993 | 1.516575 | 4.97996 | 7.46994 | 96 |
| **EV264** | 349 | 40 | -35 | 1.199995 | 0.547723 | 5.049752 | 7.574628 | 109 |
| **EV265** | 344 | 44 | -37 | 1.199995 | 0 | 2.302173 | 3.45326 | 109 |
| **EV266** | 281 | 69 | 48 | 0.489901 | 3.674235 | 0.83666 | 5.511353 | 64 |
| **EV267** | 108 | 78 | -148 | 0.4 | 0.894427 | 2.302173 | 3.45326 | 64 |
| **EV268** | 53 | 77 | 146 | 1.999974 | 1.788854 | 3.535534 | 5.303301 | 71 |
| **EV269** | 15 | 67 | 44 | 2.399956 | 4.024922 | 2.701851 | 4.052777 | 90 |
| **EV270** | 13 | 57 | -37 | 0.4 | 0.547723 | 5.049752 | 7.574628 | 88 |
| **EV271** | 16 | 52 | 26 | 0.489901 | 0 | 3.114482 | 4.671723 | 90 |
| **EV272** | 25 | 46 | -11 | 1.549197 | 0.894427 | 1.140175 | 2.323796 | 52 |
| **EV273** | 24 | 50 | 12 | 0.4 | 0.447214 | 1.30384 | 1.95576 | 52 |
| **EV274** | 308 | 82 | -9 | 0.799998 | 0 | 1.581139 | 2.371709 | 85 |
| **EV275** | 169 | 50 | -24 | 0.800006 | 0.894427 | 0.83666 | 1.341641 | 58 |
| **EV276** | 330 | 51 | -91 | 2.399956 | 0 | 1.923538 | 3.599934 | 103 |
| **EV277** | 310 | 29 | -102 | 1.999974 | 0.707107 | 4.494441 | 6.741662 | 60 |
| **EV278** | 26 | 53 | -29 | 2.79993 | 1.516575 | 4.97996 | 7.46994 | 65 |
| **EV279** | 357 | 64 | 58 | 0.799998 | 2.387467 | 2.302173 | 3.581201 | 62 |
| **EV280** | 113 | 75 | 15 | 2.79993 | 1.516575 | 4.97996 | 7.46994 | 68 |
| **EV281** | 67 | 41 | 95 | 2.712949 | 3.193744 | 0.83666 | 4.790616 | 56 |
| **EV282** | 124 | 47 | 26 | 2.79993 | 1.516575 | 4.97996 | 7.46994 | 84 |
| **EV283** | 0 | 52 | -63 | 2.399956 | 4.159327 | 4.97996 | 7.46994 | 87 |
| **EV284** | 40 | 45 | -62 | 2.79993 | 1.516575 | 4.97996 | 7.46994 | 106 |
| **EV285** | 310 | 50 | 61 | 2.399956 | 2.50998 | 5.049752 | 7.574628 | 86 |
| **EV286** | 202 | 47 | -101 | 2.79993 | 1.516575 | 4.97996 | 7.46994 | 74 |
| **EV287** | 111 | 55 | 43 | 0.800006 | 0.83666 | 0.447214 | 1.25499 | 82 |
| **EV288** | 5 | 46 | 98 | 0.894443 | 1.67332 | 1.30384 | 2.50998 | 202 |
| **EV289** | 149 | 36 | 126 | 3.430225 | 1.870829 | 5.310367 | 7.965551 | 154 |
| **EV290** | 176 | 28 | -98 | 8.54E-07 | 1.732051 | 4.037326 | 6.055989 | 77 |
| **EV291** | 229 | 40 | -104 | 0 | 2.167948 | 2.073644 | 3.251922 | 80 |
| **EV292** | 3 | 38 | -105 | 1.326673 | 2.48998 | 1.30384 | 3.73497 | 62 |
| **EV293** | 166 | 65 | -71 | 2.79993 | 0.83666 | 5.049752 | 7.574628 | 64 |
| **EV294** | 211 | 52 | -41 | 0.799998 | 0 | 1.30384 | 1.95576 | 128 |
| **EV295** | 184 | 49 | -176 | 0.4 | 0.447214 | 2.701851 | 4.052777 | 57 |
| **EV296** | 259 | 30 | 135 | 0.800006 | 0 | 0.83666 | 1.25499 | 90 |
| **EV297** | 11 | 49 | -134 | 2.856915 | 0.447214 | 1.923538 | 4.285373 | 103 |
| **EV298** | 192 | 28 | -103 | 0.799998 | 0 | 3.114482 | 4.671723 | 126 |
| **EV299** | 294 | 78 | -159 | 2.399956 | 0.894427 | 1.923538 | 3.599934 | 94 |
| **EV300** | 191 | 27 | -159 | 0.799998 | 0.447214 | 1.923538 | 2.885307 | 61 |
| **EV301** | 136 | 62 | 127 | 0 | 0.447214 | 1.140175 | 1.710263 | 88 |
| **EV302** | 192 | 73 | -20 | 0.489901 | 3.781534 | 1.30384 | 5.672301 | 104 |
| **EV303** | 144 | 53 | -133 | 0.4 | 0.894427 | 1.30384 | 1.95576 | 81 |
| **EV304** | 44 | 39 | -47 | 3.999793 | 1.224745 | 1.81659 | 5.99969 | 58 |
| **EV305** | 18 | 44 | -83 | 2.756987 | 0 | 0.894427 | 4.135481 | 86 |
| **EV306** | 264 | 45 | -157 | 0.489901 | 3.646917 | 0.447214 | 5.470376 | 87 |
| **EV307** | 53 | 43 | 49 | 2.79993 | 0.894427 | 1.581139 | 4.199895 | 84 |
| **EV308** | 48 | 43 | -146 | 2.79993 | 1.516575 | 4.97996 | 7.46994 | 117 |
| **EV309** | 43 | 32 | -53 | 2.79993 | 1.732051 | 4.219005 | 6.328508 | 92 |
| **EV310** | 169 | 41 | 81 | 3.999793 | 0 | 2.701851 | 5.99969 | 85 |
| **EV311** | 56 | 44 | -37 | 0.800006 | 3.082207 | 0.83666 | 4.623311 | 93 |
| **EV312** | 3 | 60 | -30 | 0 | 0.894427 | 2.302173 | 3.45326 | 98 |
| **EV313** | 266 | 43 | -68 | 1.599987 | 0 | 1.30384 | 2.399981 | 110 |
| **EV314** | 44 | 27 | 104 | 0.489901 | 0.447214 | 0.894427 | 1.341641 | 96 |
| **EV315** | 341 | 49 | -66 | 2.79993 | 1.516575 | 4.97996 | 7.46994 | 101 |
| **EV316** | 128 | 68 | 139 | 2.79993 | 2.073644 | 1.30384 | 4.199895 | 101 |
| **EV317** | 132 | 44 | -100 | 1.199995 | 2.236068 | 3.962323 | 5.943485 | 56 |
| **EV318** | 248 | 69 | 141 | 0.4 | 3.271085 | 3.962323 | 5.943485 | 63 |
| **EV319** | 183 | 50 | 129 | 2.993719 | 2.04939 | 1.923538 | 4.490579 | 103 |
| **EV320** | 302 | 79 | -172 | 1.999974 | 0.447214 | 2.701851 | 4.052777 | 116 |
| **EV321** | 288 | 63 | 165 | 0.799998 | 3.577709 | 1.30384 | 5.366564 | 74 |
| **EV322** | 115 | 29 | -117 | 1.599987 | 0.707107 | 3.114482 | 4.671723 | 76 |
| **EV323** | 116 | 69 | -113 | 0.799998 | 0.83666 | 1.140175 | 1.710263 | 97 |
| **EV324** | 45 | 38 | -129 | 2.712978 | 2.387467 | 3.130495 | 4.695743 | 84 |
| **EV325** | 297 | 72 | -128 | 2.79993 | 1.516575 | 4.97996 | 7.46994 | 96 |
| **EV326** | 310 | 80 | -1 | 0.748338 | 5.310367 | 2.701851 | 7.965551 | 114 |
| **EV327** | 354 | 31 | -51 | 2.399956 | 0 | 3.535534 | 5.303301 | 104 |
| **EV328** | 205 | 39 | -116 | 2.713374 | 3.49285 | 0.894427 | 5.239275 | 74 |
| **EV329** | 123 | 41 | -151 | 1.599987 | 3.391165 | 5.049752 | 7.574628 | 62 |
| **EV330** | 353 | 53 | -29 | 0.799998 | 0.894427 | 4.037326 | 6.055989 | 98 |
| **EV331** | 105 | 34 | -57 | 0.748338 | 1.224745 | 0.83666 | 1.837118 | 61 |
| **EV332** | 44 | 45 | -35 | 8.54E-07 | 0.447214 | 1.923538 | 2.885307 | 102 |
| **EV333** | 33 | 27 | -60 | 0 | 0.447214 | 1.30384 | 1.95576 | 67 |
| **EV334** | 45 | 38 | 143 | 1.199995 | 0.447214 | 1.30384 | 1.95576 | 134 |
| **EV335** | 315 | 42 | -107 | 3.137472 | 4.27785 | 1.923538 | 4.706208 | 79 |
| **EV336** | 192 | 28 | -89 | 0.748338 | 4.27785 | 1 | 6.416775 | 80 |
| **EV337** | 300 | 65 | -154 | 0.799998 | 0.707107 | 3.962323 | 5.943485 | 75 |
| **EV338** | 325 | 44 | 96 | 0.894443 | 0.894427 | 2.073644 | 3.110466 | 97 |
| **EV339** | 304 | 36 | 47 | 1.199995 | 1.643168 | 0.83666 | 2.464752 | 159 |
| **EV340** | 123 | 61 | -137 | 2.79993 | 1.516575 | 4.97996 | 7.46994 | 89 |
| **EV341** | 292 | 51 | 38 | 0.799998 | 1.341641 | 1.140175 | 2.012462 | 140 |
| **EV342** | 33 | 26 | 123 | 0.4 | 0 | 1.923538 | 2.885307 | 128 |
| **EV343** | 69 | 60 | -144 | 0.799998 | 0.894427 | 4.037326 | 6.055989 | 126 |
| **EV344** | 163 | 64 | -30 | 0 | 1.30384 | 4.147288 | 6.220932 | 110 |
| **EV345** | 47 | 56 | -37 | 0 | 2.19089 | 1.581139 | 3.286335 | 55 |
| **EV346** | 176 | 63 | 107 | 2.529792 | 5.310367 | 0.894427 | 7.965551 | 111 |
| **EV347** | 343 | 53 | -45 | 2.79993 | 1.341641 | 3.114482 | 4.671723 | 88 |
| **EV348** | 193 | 34 | -34 | 0 | 1.581139 | 0 | 2.371709 | 73 |
| **EV349** | 337 | 41 | 142 | 2.399956 | 4.024922 | 1.923538 | 6.037383 | 81 |
| **EV350** | 135 | 45 | 61 | 0.979799 | 1.516575 | 1 | 2.274863 | 80 |
| **EV351** | 58 | 54 | -159 | 0.979818 | 2.167948 | 1.581139 | 3.251922 | 80 |
| **EV352** | 259 | 90 | -172 | 1.199995 | 1.788854 | 3.535534 | 5.303301 | 115 |
| **EV353** | 273 | 74 | 137 | 2.79993 | 0.447214 | 3.361547 | 5.042321 | 120 |
| **EV354** | 207 | 58 | 160 | 1.199995 | 0 | 1.923538 | 2.885307 | 107 |
| **EV355** | 274 | 37 | -86 | 2.399956 | 2.236068 | 3.962323 | 5.943485 | 124 |
| **EV356** | 124 | 64 | 52 | 0.489901 | 0.447214 | 0.707107 | 1.060661 | 112 |
| **EV357** | 325 | 54 | -145 | 2.79993 | 1.516575 | 4.97996 | 7.46994 | 83 |
| **EV358** | 84 | 49 | 60 | 0.800006 | 3.03315 | 0.447214 | 4.549725 | 81 |
| **EV359** | 138 | 63 | -149 | 3.187473 | 4.219005 | 4.472136 | 6.708204 | 90 |
| **EV360** | 143 | 46 | 81 | 0 | 0 | 2.302173 | 3.45326 | 73 |
| **EV361** | 51 | 64 | -116 | 0.4 | 1.788854 | 1.923538 | 2.885307 | 83 |
| **EV362** | 316 | 44 | -46 | 0.4 | 0.447214 | 3.535534 | 5.303301 | 77 |
| **EV363** | 126 | 32 | -124 | 0 | 0 | 1.923538 | 2.885307 | 82 |
| **EV364** | 328 | 46 | -44 | 0 | 0 | 3.962323 | 5.943485 | 75 |
| **EV365** | 308 | 61 | -140 | 2.245247 | 1.516575 | 4.505552 | 6.758328 | 66 |
| **EV366** | 196 | 43 | -29 | 4.89901 | 0.894427 | 1.30384 | 7.348515 | 66 |
| **EV367** | 99 | 26 | -97 | 0.4 | 2.44949 | 1.643168 | 3.674235 | 99 |
| **EV368** | 21 | 53 | 129 | 2.399956 | 0.894427 | 2.302173 | 3.599934 | 81 |
| **EV369** | 52 | 61 | 117 | 0.800006 | 0.707107 | 1.140175 | 1.710263 | 101 |
| **EV370** | 126 | 37 | -132 | 2.79993 | 1.516575 | 4.97996 | 7.46994 | 101 |
| **EV371** | 112 | 60 | -150 | 0 | 0.894427 | 1.30384 | 1.95576 | 72 |
| **EV372** | 152 | 32 | 135 | 1.599987 | 0 | 3.535534 | 5.303301 | 80 |
| **EV373** | 8 | 39 | -57 | 3.199895 | 1.095445 | 4.438468 | 6.657702 | 95 |
| **EV374** | 144 | 25 | 120 | 2.399956 | 2.607681 | 4.219005 | 6.328508 | 100 |
| **EV375** | 304 | 56 | 44 | 2.79993 | 1.516575 | 4.97996 | 7.46994 | 58 |
| **EV376** | 357 | 60 | 120 | 2.79993 | 1.516575 | 4.97996 | 7.46994 | 72 |
| **EV377** | 2 | 42 | 48 | 2.399956 | 0 | 3.962323 | 5.943485 | 101 |
| **EV378** | 133 | 50 | -102 | 0 | 1.341641 | 1.30384 | 2.012462 | 95 |
| **EV379** | 163 | 37 | -106 | 1.200035 | 2.167948 | 1.224745 | 3.251922 | 53 |
| **EV380** | 197 | 41 | -82 | 0.799998 | 0 | 1.30384 | 1.95576 | 62 |
| **EV381** | 180 | 70 | -117 | 2.79993 | 1.516575 | 4.97996 | 7.46994 | 104 |
| **EV382** | 12 | 51 | 87 | 0.632457 | 0.447214 | 0.83666 | 1.25499 | 88 |
| **EV383** | 359 | 50 | -55 | 1.999974 | 3.130495 | 3.114482 | 4.695743 | 63 |
| **EV384** | 199 | 47 | -19 | 1.199995 | 0.894427 | 1.30384 | 1.95576 | 93 |
| **EV385** | 148 | 35 | -154 | 0.489901 | 2.701851 | 0.83666 | 4.052777 | 89 |
| **EV386** | 35 | 62 | -26 | 0.4 | 0 | 1.30384 | 1.95576 | 57 |
| **EV387** | 90 | 44 | -102 | 1.673423 | 1.788854 | 0.547723 | 2.683281 | 87 |
| **EV388** | 68 | 53 | 138 | 2.79993 | 3.130495 | 1.923538 | 4.695743 | 75 |
| **EV389** | 243 | 26 | -94 | 0 | 0.447214 | 1.140175 | 1.710263 | 110 |
| **EV390** | 183 | 48 | -27 | 0.489901 | 2.302173 | 0 | 3.45326 | 61 |
| **EV391** | 149 | 25 | -131 | 0 | 0 | 4.549725 | 6.824588 | 57 |
| **EV392** | 346 | 79 | 5 | 8.54E-07 | 4.086563 | 0 | 6.129845 | 125 |
| **EV393** | 294 | 51 | 63 | 2.79993 | 0.83666 | 5.049752 | 7.574628 | 59 |
| **EV394** | 106 | 32 | -137 | 2.399956 | 1.341641 | 3.535534 | 5.303301 | 67 |
| **EV395** | 27 | 36 | -73 | 0.4 | 0 | 3.114482 | 4.671723 | 62 |
| **EV396** | 39 | 40 | 102 | 0.632457 | 0 | 4.669047 | 7.003571 | 123 |
| **EV397** | 16 | 40 | -37 | 2.79993 | 1.516575 | 4.97996 | 7.46994 | 86 |
| **EV398** | 346 | 63 | 39 | 8.54E-07 | 1.30384 | 2.683282 | 4.024923 | 75 |
| **EV399** | 56 | 34 | -48 | 2.79993 | 1.516575 | 4.97996 | 7.46994 | 68 |
| **EV400** | 69 | 54 | 59 | 0.799998 | 0.447214 | 1.140175 | 1.710263 | 55 |
| **EV401** | 94 | 55 | -92 | 1.599987 | 1.516575 | 4.97996 | 7.46994 | 90 |
| **EV402** | 141 | 53 | -159 | 0.800006 | 3.193744 | 0.447214 | 4.790616 | 60 |
| **EV403** | 142 | 46 | -149 | 0.799998 | 0.894427 | 4.037326 | 6.055989 | 83 |
| **EV404** | 143 | 43 | 147 | 0.799998 | 0.894427 | 1.923538 | 2.885307 | 72 |
| **EV405** | 149 | 62 | -126 | 2.79993 | 1.516575 | 4.97996 | 7.46994 | 65 |
| **EV406** | 69 | 33 | 55 | 2.79993 | 1.516575 | 4.97996 | 7.46994 | 69 |
| **EV407** | 144 | 79 | -168 | 2.79993 | 1.516575 | 4.97996 | 7.46994 | 80 |
| **EV408** | 29 | 49 | -32 | 1.199995 | 1.341641 | 3.962323 | 5.943485 | 60 |

Figure 2: Beachballs and station distribution of earthquakes in Abu Dabbab region

| 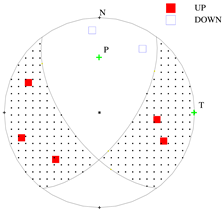 Focal mechanism of event no.1 | 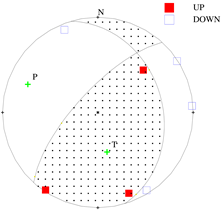 Focal mechanism of event no.2 |
| --- | --- |
| 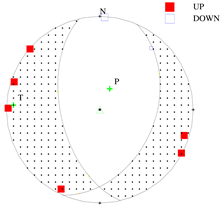 Focal mechanism of event no.3 | 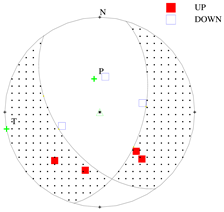 Focal mechanism of event no.4 |
| 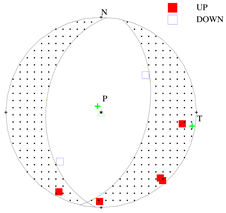 Focal mechanism of event no.5 | 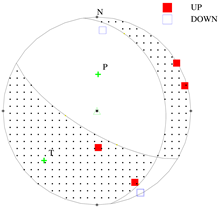 Focal mechanism of event no.6 |

| Focal mechanism of event no.7 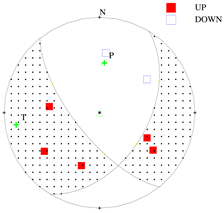 | Focal mechanism of event no.8 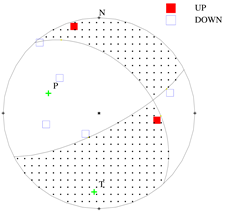 |
| --- | --- |
| Focal mechanism of event no.9 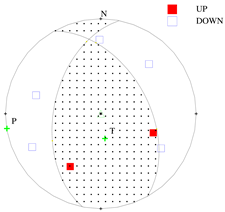 | Focal mechanism of event no.10 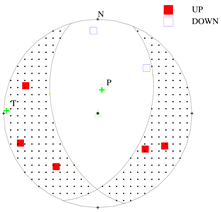 |
| Focal mechanism of event no.11 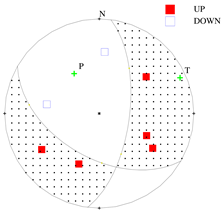 | Focal mechanism of event no.12 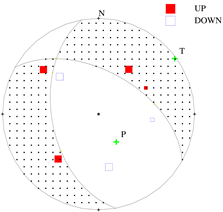 |

| Focal mechanism of event no.13 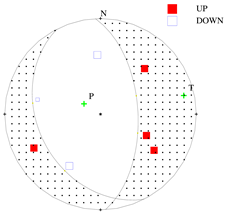 | Focal mechanism of event no.14 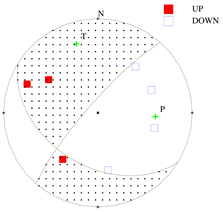 |
| --- | --- |
| Focal mechanism of event no. 15 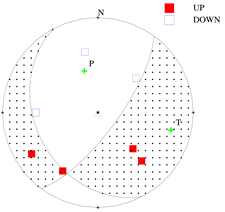 | Focal mechanism of event no.16 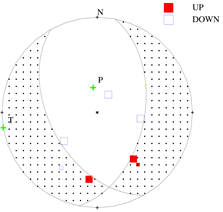 |
| Focal mechanism of event no.17 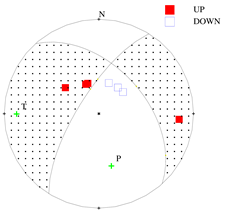 | Focal mechanism of event no.18 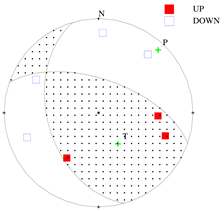 |

| Focal mechanism of event no.19 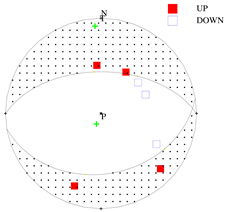 | Focal mechanism of event no.20 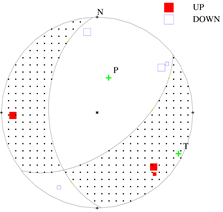 |
| --- | --- |
| Focal mechanism of event no. 21 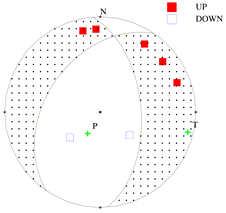 | Focal mechanism of event no.22 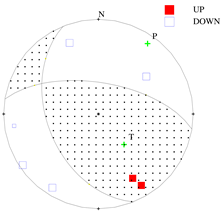 |
| Focal mechanism of event no.23 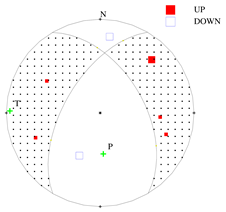 | Focal mechanism of event no.24 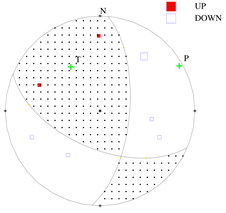 |

| Focal mechanism of event no.25 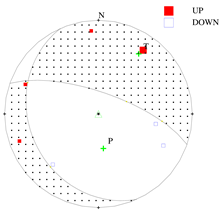 | Focal mechanism of event no.26 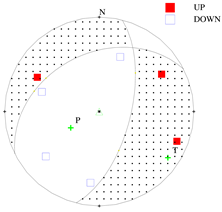 |
| --- | --- |
| Focal mechanism of event no. 27 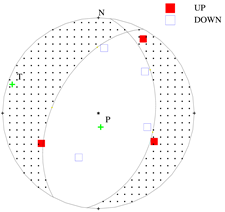 | Focal mechanism of event no.28 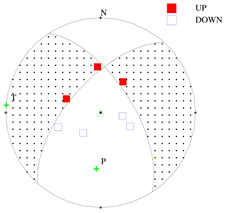 |
| Focal mechanism of event no.29 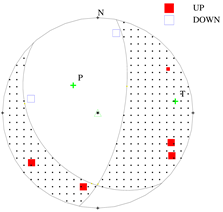 | Focal mechanism of event no.30 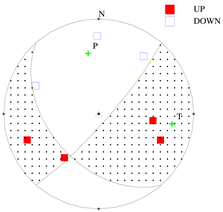 |

| Focal mechanism of event no.31 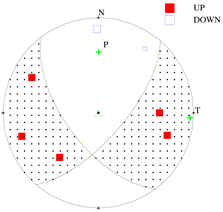 | Focal mechanism of event no.32 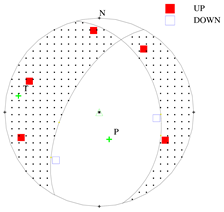 |
| --- | --- |
| Focal mechanism of event no. 33 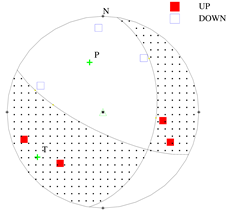 | Focal mechanism of event no.34 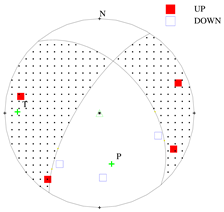 |
| Focal mechanism of event no.35 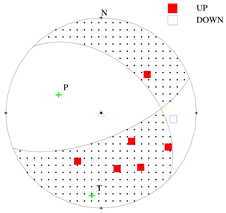 | Focal mechanism of event no.36 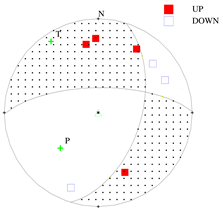 |

| Focal mechanism of event no.37 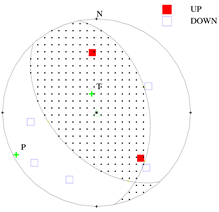 | Focal mechanism of event no.38 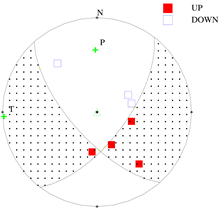 |
| --- | --- |
| Focal mechanism of event no. 39 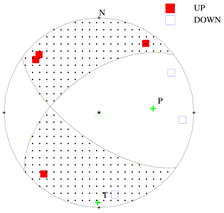 | Focal mechanism of event no.40 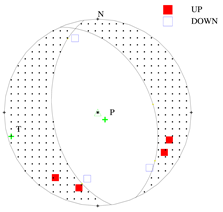 |
| Focal mechanism of event no.41 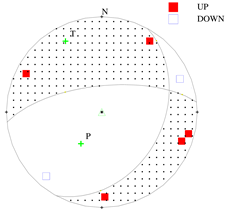 | Focal mechanism of event no.42 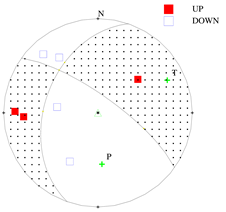 |

| Focal mechanism of event no.43 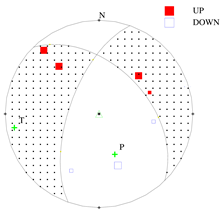 | Focal mechanism of event no.44 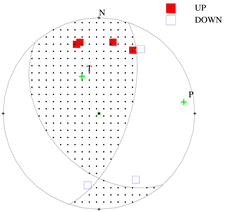 |
| --- | --- |
| Focal mechanism of event no.45 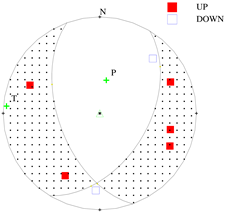 | Focal mechanism of event no.46 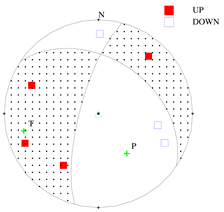 |
| Focal mechanism of event no.47 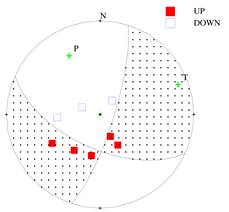 | Focal mechanism of event no.48 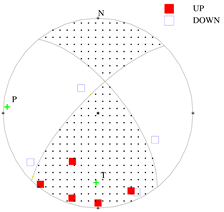 |

| Focal mechanism of event no.49 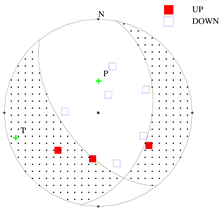 | Focal mechanism of event no.50 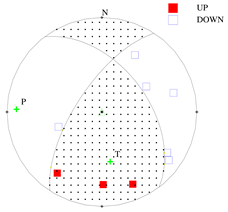 |
| --- | --- |
| Focal mechanism of event no.51 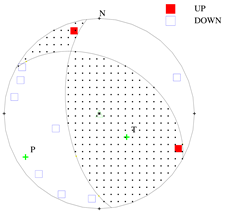 | Focal mechanism of event no.52 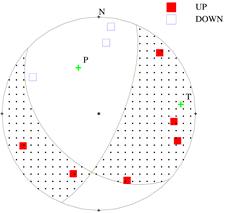 |
| Focal mechanism of event no.53 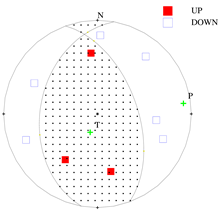 | Focal mechanism of event no.54 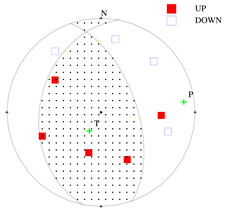 |

| Focal mechanism of event no.55 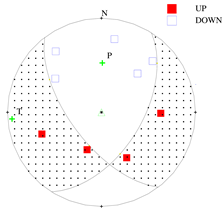 | Focal mechanism of event no.56 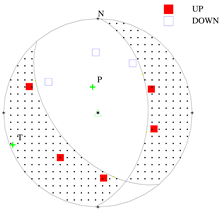 |
| --- | --- |
| Focal mechanism of event no.57 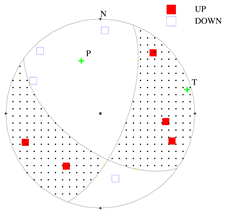 | Focal mechanism of event no.58 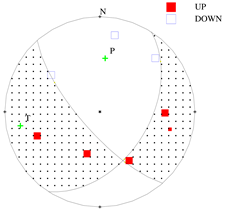 |
| Focal mechanism of event no.59 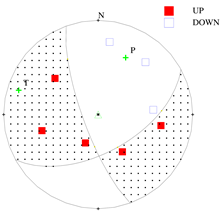 | Focal mechanism of event no.60 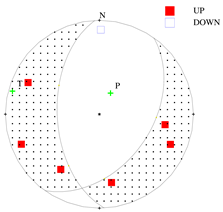 |

| Focal mechanism of event no.61 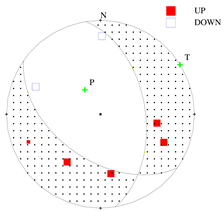 | Focal mechanism of event no.62 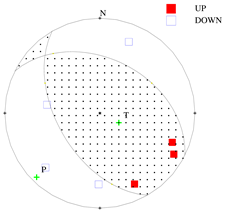 |
| --- | --- |
| Focal mechanism of event no.63 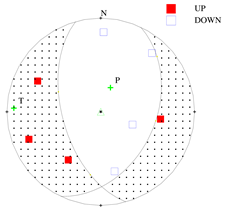 | Focal mechanism of event no.64 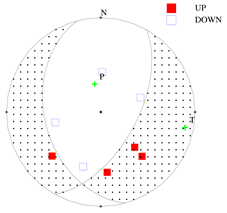 |
| Focal mechanism of event no.65 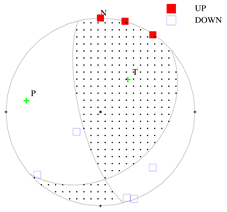 | Focal mechanism of event no.66 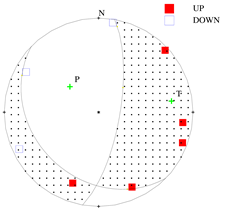 |

| Focal mechanism of event no.67 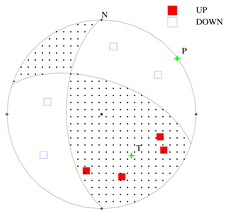 | Focal mechanism of event no.68 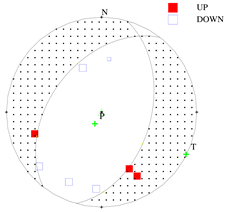 |
| --- | --- |
| Focal mechanism of event no.69 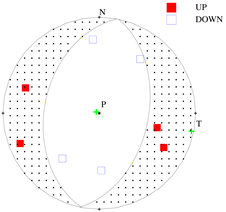 | Focal mechanism of event no.70 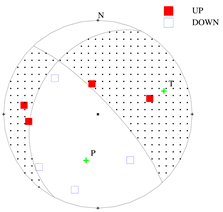 |
| Focal mechanism of event no.71 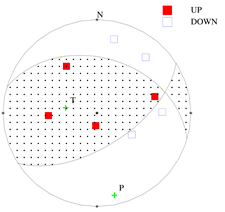 | Focal mechanism of event no.72 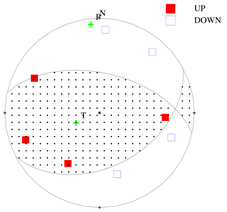 |

| Focal mechanism of event no.73 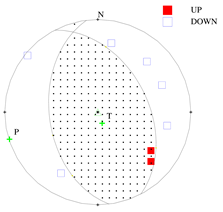 | Focal mechanism of event no.74 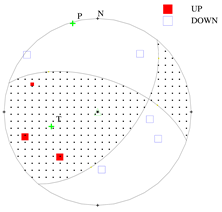 |
| --- | --- |
| Focal mechanism of event no.75 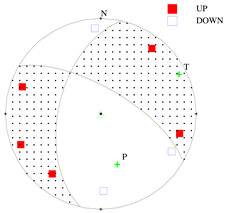 | Focal mechanism of event no.76 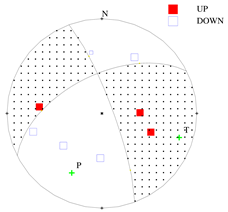 |
| Focal mechanism of event no.77 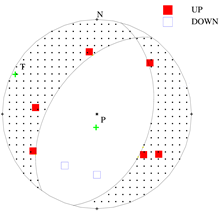 | Focal mechanism of event no.78 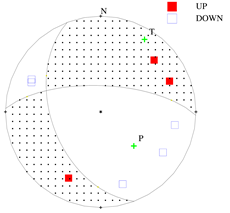 |

| Focal mechanism of event no.79 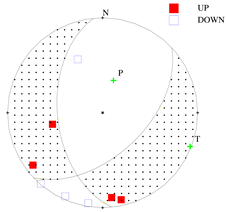 | Focal mechanism of event no.80 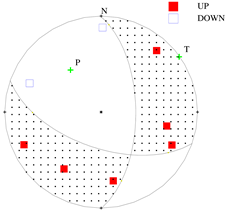 |
| --- | --- |
| Focal mechanism of event no.81 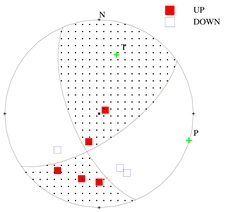 | Focal mechanism of event no.82 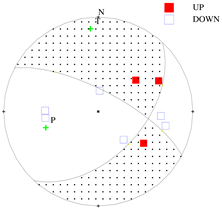 |
| Focal mechanism of event no.83 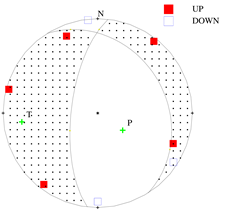 | Focal mechanism of event no.84 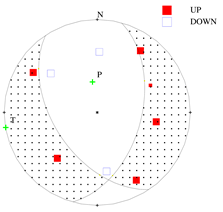 |

| Focal mechanism of event no.85 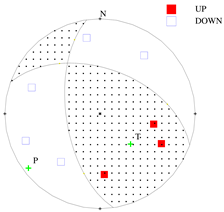 | Focal mechanism of event no.86 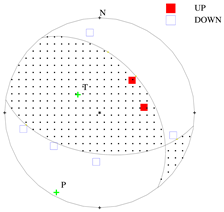 |
| --- | --- |
| Focal mechanism of event no.87 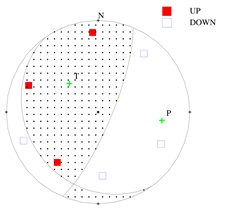 | Focal mechanism of event no.88 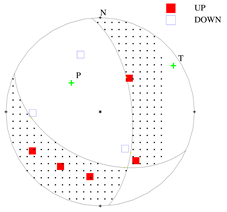 |
| Focal mechanism of event no.89 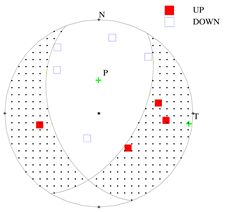 | Focal mechanism of event no.90 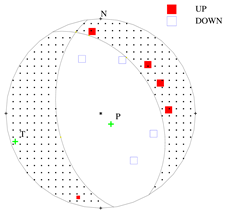 |

| Focal mechanism of event no.91 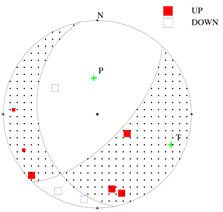 | Focal mechanism of event no.92 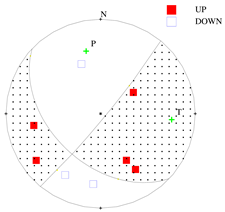 |
| --- | --- |
| Focal mechanism of event no.93 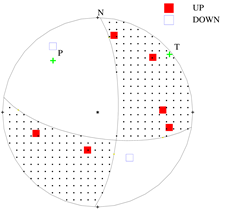 | Focal mechanism of event no.94 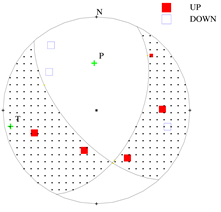 |
| Focal mechanism of event no.95 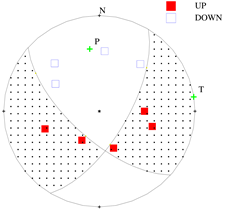 | Focal mechanism of event no.96 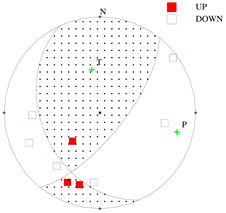 |

| Focal mechanism of event no.97 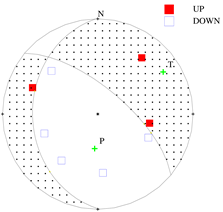 | Focal mechanism of event no.98 |
| --- | --- |
| Focal mechanism of event no.99 | Focal mechanism of event no.100 |
| Focal mechanism of event no.101 | Focal mechanism of event no.102 |

| Focal mechanism of event no.103 | Focal mechanism of event no.104 |
| --- | --- |
| Focal mechanism of event no.105 | Focal mechanism of event no.106 |
| Focal mechanism of event no.107 | Focal mechanism of event no.108 |

| Focal mechanism of event no.109 | Focal mechanism of event no.110 |
| --- | --- |
| Focal mechanism of event no.111 | Focal mechanism of event no.112 |
| Focal mechanism of event no.113 | Focal mechanism of event no.114 |

| Focal mechanism of event no.115 | Focal mechanism of event no.116 |
| --- | --- |
| Focal mechanism of event no.117 | Focal mechanism of event no.118 |
| Focal mechanism of event no.119 | Focal mechanism of event no.120 |

| Focal mechanism of event no.121 | Focal mechanism of event no.122 |
| --- | --- |
| Focal mechanism of event no.123 | Focal mechanism of event no.124 |
| Focal mechanism of event no.125 | Focal mechanism of event no.126 |

| Focal mechanism of event no.127 | Focal mechanism of event no.128 |
| --- | --- |
| Focal mechanism of event no.129 | Focal mechanism of event no.130 |
| Focal mechanism of event no.131 | Focal mechanism of event no.132 |
| Focal mechanism of event no.133 | Focal mechanism of event no.134 |
| Focal mechanism of event no.135 | Focal mechanism of event no.136 |
| Focal mechanism of event no.137 | Focal mechanism of event no.138 |

| Focal mechanism of event no.139 | Focal mechanism of event no.140 |
| --- | --- |
| Focal mechanism of event no.141 | Focal mechanism of event no.142 |
| Focal mechanism of event no.143 | Focal mechanism of event no.144 |

| Focal mechanism of event no.145 | Focal mechanism of event no.146 |
| --- | --- |
| Focal mechanism of event no.147 | Focal mechanism of event no.148 |
| Focal mechanism of event no.149 | Focal mechanism of event no.150 |

| Focal mechanism of event no.151 | Focal mechanism of event no.152 |
| --- | --- |
| Focal mechanism of event no.153 | Focal mechanism of event no.154 |
| Focal mechanism of event no.155 | Focal mechanism of event no.156 |

| Focal mechanism of event no.157 | Focal mechanism of event no.158 |
| --- | --- |
| Focal mechanism of event no.159 | Focal mechanism of event no.160 |
| Focal mechanism of event no.161 | Focal mechanism of event no.162 |

| Focal mechanism of event no.163 | Focal mechanism of event no.164 |
| --- | --- |
| Focal mechanism of event no.165 | Focal mechanism of event no.166 |
| Focal mechanism of event no.167 | Focal mechanism of event no.168 |

| Focal mechanism of event no.169 | Focal mechanism of event no.170 |
| --- | --- |
| Focal mechanism of event no.171 | Focal mechanism of event no.172 |
| Focal mechanism of event no.173 | Focal mechanism of event no.174 |

| Focal mechanism of event no.175 | Focal mechanism of event no.176 |
| --- | --- |
| Focal mechanism of event no.177 | Focal mechanism of event no.178 |
| Focal mechanism of event no.179 | Focal mechanism of event no.180 |

| Focal mechanism of event no.181 | Focal mechanism of event no.182 |
| --- | --- |
| Focal mechanism of event no.183 | Focal mechanism of event no.184 |
| Focal mechanism of event no.185 | Focal mechanism of event no.186 |

| Focal mechanism of event no.187 | Focal mechanism of event no.188 |
| --- | --- |
| Focal mechanism of event no.189 | Focal mechanism of event no.190 |
| Focal mechanism of event no.191 | Focal mechanism of event no.192 |

| Focal mechanism of event no.193 | Focal mechanism of event no.194 |
| --- | --- |
| Focal mechanism of event no.195 | Focal mechanism of event no.196 |
| Focal mechanism of event no.197 | Focal mechanism of event no.198 |

| Focal mechanism of event no.199 | Focal mechanism of event no.200 |
| --- | --- |
| Focal mechanism of event no.201 | Focal mechanism of event no.202 |
| Focal mechanism of event no.203 | Focal mechanism of event no.204 |

| Focal mechanism of event no.205 | Focal mechanism of event no.206 |
| --- | --- |
| Focal mechanism of event no.207 | Focal mechanism of event no.208 |
| Focal mechanism of event no.209 | Focal mechanism of event no.210 |

| Focal mechanism of event no.211 | Focal mechanism of event no.212 |
| --- | --- |
| Focal mechanism of event no.213 | Focal mechanism of event no.214 |
| Focal mechanism of event no.215 | Focal mechanism of event no.216 |

| Focal mechanism of event no.217 | Focal mechanism of event no.218 |
| --- | --- |
| Focal mechanism of event no.219 | Focal mechanism of event no.220 |
| Focal mechanism of event no.221 | Focal mechanism of event no.222 |

| Focal mechanism of event no.223 | Focal mechanism of event no.224 |
| --- | --- |
| Focal mechanism of event no.225 | Focal mechanism of event no.226 |
| Focal mechanism of event no.227 | Focal mechanism of event no.228 |

| Focal mechanism of event no.229 | Focal mechanism of event no.230 |
| --- | --- |
| Focal mechanism of event no.231 | Focal mechanism of event no.232 |
| Focal mechanism of event no.233 | Focal mechanism of event no.234 |

| Focal mechanism of event no.235 | Focal mechanism of event no.236 |
| --- | --- |
| Focal mechanism of event no.237 | Focal mechanism of event no.238 |
| Focal mechanism of event no.239 | Focal mechanism of event no.240 |

| Focal mechanism of event no.241 | Focal mechanism of event no.242 |
| --- | --- |
| Focal mechanism of event no.243 | Focal mechanism of event no.244 |
| Focal mechanism of event no.245 | Focal mechanism of event no.246 |

| Focal mechanism of event no.247 | Focal mechanism of event no.248 |
| --- | --- |
| Focal mechanism of event no.249 | Focal mechanism of event no.250 |
| Focal mechanism of event no.251 | Focal mechanism of event no.252 |

| Focal mechanism of event no.253 | Focal mechanism of event no.254 |
| --- | --- |
| Focal mechanism of event no.255 | Focal mechanism of event no.256 |
| Focal mechanism of event no.257 | Focal mechanism of event no.258 |

| Focal mechanism of event no.259 | Focal mechanism of event no.260 |
| --- | --- |
| Focal mechanism of event no.261 | Focal mechanism of event no.262 |
| Focal mechanism of event no.263 | Focal mechanism of event no.264 |

| Focal mechanism of event no.265 | Focal mechanism of event no.266 |
| --- | --- |
| Focal mechanism of event no.267 | Focal mechanism of event no.268 |
| Focal mechanism of event no.269 | Focal mechanism of event no.270 |

| Focal mechanism of event no.271 | Focal mechanism of event no.272 |
| --- | --- |
| Focal mechanism of event no.273 | Focal mechanism of event no.274 |
| Focal mechanism of event no.275 | Focal mechanism of event no.276 |

| Focal mechanism of event no.277 | Focal mechanism of event no.278 |
| --- | --- |
| Focal mechanism of event no.279 | Focal mechanism of event no.280 |
| Focal mechanism of event no.281 | Focal mechanism of event no.282 |

| Focal mechanism of event no.283 | Focal mechanism of event no.284 |
| --- | --- |
| Focal mechanism of event no.285 | Focal mechanism of event no.286 |
| Focal mechanism of event no.287 | Focal mechanism of event no.288 |

| Focal mechanism of event no.289 | Focal mechanism of event no.290 |
| --- | --- |
| Focal mechanism of event no.291 | Focal mechanism of event no.292 |
| Focal mechanism of event no.293 | Focal mechanism of event no.294 |

| Focal mechanism of event no.295 | Focal mechanism of event no.296 |
| --- | --- |
| Focal mechanism of event no.297 | Focal mechanism of event no.298 |
| Focal mechanism of event no.299 | Focal mechanism of event no.300 |

| Focal mechanism of event no.301 | Focal mechanism of event no.302 |
| --- | --- |
| Focal mechanism of event no.303 | Focal mechanism of event no.304 |
| Focal mechanism of event no.305 | Focal mechanism of event no.306 |

| Focal mechanism of event no.307 | Focal mechanism of event no.308 |
| --- | --- |
| Focal mechanism of event no.309 | Focal mechanism of event no.310 |
| Focal mechanism of event no.311 | Focal mechanism of event no.312 |

| Focal mechanism of event no.313 | Focal mechanism of event no.314 |
| --- | --- |
| Focal mechanism of event no.315 | Focal mechanism of event no.316 |
| Focal mechanism of event no.317 | Focal mechanism of event no.318 |

| Focal mechanism of event no.319 | Focal mechanism of event no.320 |
| --- | --- |
| Focal mechanism of event no.321 | Focal mechanism of event no.322 |
| Focal mechanism of event no.323 | Focal mechanism of event no.324 |

| Focal mechanism of event no.325 | Focal mechanism of event no.326 |
| --- | --- |
| Focal mechanism of event no.327 | Focal mechanism of event no.328 |
| Focal mechanism of event no.329 | Focal mechanism of event no.330 |

| Focal mechanism of event no.331 | Focal mechanism of event no.332 |
| --- | --- |
| Focal mechanism of event no.333 | Focal mechanism of event no.334 |
| Focal mechanism of event no.335 | Focal mechanism of event no.336 |

| Focal mechanism of event no.337 | Focal mechanism of event no.338 |
| --- | --- |
| Focal mechanism of event no.339 | Focal mechanism of event no.340 |
| Focal mechanism of event no.341 | Focal mechanism of event no.342 |

| Focal mechanism of event no.343 | Focal mechanism of event no.344 |
| --- | --- |
| Focal mechanism of event no.345 | Focal mechanism of event no.346 |
| Focal mechanism of event no.347 | Focal mechanism of event no.348 |

| Focal mechanism of event no.349 | Focal mechanism of event no.350 |
| --- | --- |
| Focal mechanism of event no.351 | Focal mechanism of event no.352 |
| Focal mechanism of event no.353 |  |

|  | Focal mechanism of event no.356 |
| --- | --- |
| Focal mechanism of event no.357 | Focal mechanism of event no.358 |
| Focal mechanism of event no.359 | Focal mechanism of event no.360 |

| Focal mechanism of event no.361 | Focal mechanism of event no.362 |
| --- | --- |
| Focal mechanism of event no.363 | Focal mechanism of event no.364 |
| Focal mechanism of event no.365 | Focal mechanism of event no.366 |

| Focal mechanism of event no.367 | Focal mechanism of event no.368 |
| --- | --- |
| Focal mechanism of event no.369 | Focal mechanism of event no.370 |
| Focal mechanism of event no.371 | Focal mechanism of event no.372 |

| Focal mechanism of event no.373 | Focal mechanism of event no.374 |
| --- | --- |
| Focal mechanism of event no.375 | Focal mechanism of event no.376 |
| Focal mechanism of event no.377 | Focal mechanism of event no.378 |

| Focal mechanism of event no.379 | Focal mechanism of event no.380 |
| --- | --- |
| Focal mechanism of event no.381 | Focal mechanism of event no.382 |
| Focal mechanism of event no.383 | Focal mechanism of event no.384 |

| Focal mechanism of event no.385 | Focal mechanism of event no.386 |
| --- | --- |
| Focal mechanism of event no.387 | Focal mechanism of event no.388 |
| Focal mechanism of event no.389 | Focal mechanism of event no.390 |
| Focal mechanism of event no.391 | Focal mechanism of event no.392 |
| Focal mechanism of event no.393 | Focal mechanism of event no.394 |
| Focal mechanism of event no.395 | Focal mechanism of event no.396 |

| Focal mechanism of event no.397 | Focal mechanism of event no.398 |
| --- | --- |
| Focal mechanism of event no.399 | Focal mechanism of event no.400 |
| Focal mechanism of event no.401 | Focal mechanism of event no.402 |

| Focal mechanism of event no.403 | Focal mechanism of event no.404 |
| --- | --- |
| Focal mechanism of event no.405 | Focal mechanism of event no.406 |
| Focal mechanism of event no.407 | Focal mechanism of event no.408 |
